# Supplementary material for: Charting the Biosynthetic Landscape of Hybrid Polyketide-Nonribosomal Peptide-Specialized Lipids
Source: JACS Au. 2026 Jun 8;6(6):3363–75. doi: 10.1021/jacsau.6c00386 (PMC13291983; doi:10.1021/jacsau.6c00386)
Supplement: Supplementary file 1 [file au6c00386_si_001.pdf]

**Supplementary Information**

# Charting the Biosynthetic Landscape of Hybrid Polyketide-Nonribosomal Peptide-Specialized Lipids

Fatima El Arnouki Belhaji[a,b], Dries De Ruysscher[a,b], Giel Vanreppelen[a,b,c], Laura-Lynn Huybrechts[a,b], Mohammad M. Alanjary[d], Mitja M. Zdouc[d,e], Emmanuel L. C. de los Santos[f], Joachim Demaerel[g], Ewoud Vaneeckhaute[h], Odessa Van Goethem[c], Hans Gerstmans[a,b,i], Eric Breynaert[h], Patrick Van Dijck[c], Marnix H. Medema[d], Angus N. M. Weir[a,b,j], Eveline Lescrinier[k], Joleen Masschelein[a,b]*

[a] Laboratory for Biomolecular Discovery and Engineering, Department of Biology, KU Leuven, 3001 Leuven (Belgium)

[b] VIB Center for Microbiology, VIB, 3001 Leuven (Belgium)

[c] Laboratory of Molecular Cell Biology, Department of Biology, KU Leuven, 3001 Leuven (Belgium)

[d] Bioinformatics Group, Wageningen University & Research, 6700 AP Wageningen (The Netherlands)

[e] Department of Pharmaceutical Sciences, Division of Pharmacognosy, University of Vienna, 1090 Vienna (Austria)

[f] Department of Chemistry, University of Warwick, CV4 7AL Coventry (UK)

[g] Laboratory of Organic Synthesis, Department of Chemistry, KU Leuven, 3001 Leuven (Belgium)

[h] NMRCoRe, NMR/X-Ray platform for Convergence Research, KU Leuven, 3001 Leuven (Belgium)

[i] Biosensors Group, Department of Biosystems, KU Leuven, 3001 Leuven (Belgium)

[j] The Rosalind Franklin Institute, Harwell Science & Innovation Campus, OX11 0FA Harwell (UK)

[k] Laboratory for Medicinal Chemistry, Department of Pharmaceutical and Pharmacological Sciences, Rega Institute for Medical Research, KU Leuven, 3000 Leuven (Belgium)

*joleen.masschelein@kuleuven.be

## Table of Contents

**Experimental Procedures** ........ **3**

Bacterial strains, plasmids, media and growth conditions ........ 3

Genome mining for novel hybrid polyketide-nonribosomal peptide-specialized lipid biosynthetic pathways ........ 3

Insertional mutagenesis of chitinimine biosynthetic gene *chtnA* ........ 3

Comparative metabolic profiling and UHPL-ESI-Q-TOF-MS(/MS) analyses ........ 4

Isolation and structure elucidation of the chitinimines ........ 5

Determination of absolute stereochemical configurations using Marfey's method ........ 5

Acid hydrolysis of the chitinimines ........ 6

DFT calculations ........ 6

Phylogenetic and bioinformatic analysis of the chitinimine biosynthetic pathway ........ 7

Bioactivity assays ........ 8

**Results and Discussion** ........ **10**

Supplementary Figures ........ 10

Supplementary Tables ........ 48

**References** ........ **76**

## Experimental Procedures

### Bacterial strains, plasmids, media and growth conditions

*Chitinimonas koreensis* DSM 17726, first described by Kim *et al*. (2006), was obtained from the Deutsche Sammlung von Mikroorganismen und Zellkulturen (DSMZ, Leibniz-Institut, Germany, DSM no. 17726) and used for chitinimine production.[1] The strain was cultured at 28°C in Reasoner's 2A (R2A) medium for liquid cultivation and on R2A agar (R2A supplemented with 15 g/L agar) for solid cultivation. Standard cloning procedures were performed using *Escherichia coli* SY327.[2] The auxotrophic strain *E. coli* RHO3 was used for conjugative transfer of DNA into wildtype (WT) *C. koreensis*.[3] *E. coli* RHO3 was grown in Lysogeny Broth (LB) supplemented with 200 µg/mL 2,6-diaminopimelic acid (DAP) at 37°C. The suicide vector pSF100 was used for insertional mutagenesis in *C. koreensis*.[4] For plasmid selection, kanamycin at 50 µg/mL was used.

### Genome mining for novel hybrid polyketide-nonribosomal peptide-specialized lipid biosynthetic pathways

Genome mining for novel hybrid PKS-NRPS-PUFA synthase-like clusters was carried out using antiSMASH (version 7.1.0), which was installed locally as a conda package.[5] A dataset comprising 62,706 NRPS-containing bacterial genomic regions in Genbank format, obtained from the antiSMASH database (version 4), was analyzed via a custom-designed search rule, which we termed 'zeamine-like'.[6] This rule integrates pre-existing profile Hidden Markov Models (pHMMs) targeting PKS, NRPS and PUFA synthase-like biosynthetic machinery (cds(Condensation and (AMP-binding or A-OX)) and cds(PKS_AT and (PKS_KS or ene_KS or mod_KS or hyb_KS or itr_KS or tra_KS)) and (hglE or hglD or PUFA_KS)). A 'relaxed' level of strictness was applied, allowing for the detection of incomplete clusters lacking one or more functional components. The maximum allowed distance between core genes was set at 20 kbp, and an additional 20 kbp was included beyond the core genes to define the protocluster boundaries.

All positive hits were manually curated and further analysed via antiSMASH with all optional parameters enabled. Particular attention was given to the *KnownClusterBlast* tool, which compares query clusters against the MIBiG database to identify similarities with experimentally characterized biosynthetic gene clusters (BGCs).[7] Initial hits were excluded if (i) the predicted PKS gene corresponded to a misannotated *pfaBC* homolog, (ii) the co-localization and synteny of PKS-, NRPS-, and PUFA synthase-like genes were not conserved among closely related strains, as determined using the *ClusterBlast* tool in antiSMASH, (iii) the *pfa*-like and PKS-NRPS genes were separated by numerous intervening genes lacking operon-like organization, and/or (iv) more than 71% of the biosynthetic genes matched a known MIBiG reference cluster, indicating the likely detection of a known BGC.

Sequences of either ketosynthase (KS) domains encoded in *pfaA* homologs (**Table S12**) or KS-CLF heterodimers encoded in *pfaC*-like genes (**Table S13**) were manually extracted and aligned using Clustal Omega via Geneious Prime (version 2024.0.5). Maximum likelihood phylogenetic trees were constructed using the IQ-TREE web server with default parameters and 10,000 bootstrap alignments.[8] The resulting trees were visualized with interactive Tree Of Life (iTOL) (version 7.1).[9]

To explore the diversity of zeamine and fabclavine BGCs from both known producers (e.g., *Dickeya* and *Serratia* spp.) and newly identified ones (e.g., *Chitinivorax tropicus*, *Paludibacterium paludis* and *Bowmanella denitrifican*s), the Biosynthetic Gene Similarity Clustering And Prospecting Engine (BiG-SCAPE) (version 2.0.0-beta.5) tool was used.[10] The Affinity Propagation's internal preference parameter was set at a negative value and the cutoff was maximized (value of 0.99) to minimize the formation of multiple Gene Cluster Families (GCFs).

Comparative analyses of BGCs and/or genomic regions were visualized with the clinker tool from the CompArative GEne Cluster Analysis Toolbox (CAGECAT).[11]

### Insertional mutagenesis of chitinimine biosynthetic gene *chtnA*

Insertional mutagenesis was performed using the *pir* replication-dependent pSF100 suicide plasmid. Primers amplifying a ~1000 bp region of the gene *chtnA* (Fw: 5'-AGGTCTCATATCGCCATCATCGGCGCCGCCTGCC-3'; Rev: 5'-TGGTCTCAGCTCCCGCTTCGAGGTGGCCGATATTGGTCTTGATCGAGCC-3') were designed with *Bsa*I restriction sites at the 5'-end to enable directional cloning of the PCR products into pSF100. Analogous primers were designed to amplify the pSF100 plasmid (Fw: 5'-TGGTCTCAGATATCGCATGCGGTACCTCTAGAAG-3'; Rev: 5'-AGGTCTCAGAGCTCTCCCGGGAATTCGATC-3'). The PCR products were digested with *Bsa*I and ligated using T4 DNA ligase. All enzymes and kits were purchased from Thermo Fisher Scientific and used according to the manufacturer's instructions unless otherwise stated. Next, chemically competent *E. coli* SY327 cells were transformed with the ligation

mixtures and transformants were selected on LB agar plates supplemented with 50 µg/mL kanamycin. Plasmids were isolated from kanamycin-resistant colonies using the GeneJET plasmid miniprep kit and their sequence was confirmed by Sanger sequencing (Eurofins genomics) using vector primers (Fw: 5'-GCGATTCAGGCCTGGTATG-3'; Rev: 5'-CGCACTGAGAAGCCCTTAG-3').The validated construct was introduced into chemically competent *E. coli* RHO3 cells and transformants were selected on LB agar plates supplemented with 200 µg/mL DAP and 50 µg/mL kanamycin. For conjugative transfer of the construct into WT *C. koreensis,* a biparental mating protocol from Garcia (2018) was adapted.[12] Specifically, liquid cultures of *C. koreensis* and *E. coli* RHO3 were cultured overnight and subsequently diluted to a final optical density at 600 nm ($OD_{600}$) of 0.5 – 0.7. *E. coli* cells were subsequently harvested via centrifugation (10 min, 4600 rpm) and resuspended in 5 mL of liquid R2A medium. *C. koreensis* acceptor cells were spread with a sterile swap on half of an R2A agar plate supplemented with 200 µg/mL DAP and 10 mM $MgCl_2$. *E. coli* RHO3 donor cells were spread on top of the acceptor cells. One fourth of the plate was streaked with *C. koreensis* control cells and the other fourth with *E. coli* RHO3 control cells. The bacteria were incubated at 28°C for 48 hours. Bacteria from each section were collected with a swap and restreaked onto selective R2A agar plates supplemented with 50 µg/mL kanamycin, 10 mM $MgCl_2$ and no DAP. Plates were incubated at 28°C for five days to observe *C. koreensis* cells in the conjugation section, with no growth in the control areas. The correct integration of pSF100 into the genomic DNA of *C. koreensis* was verified via junction PCR and Sanger sequencing. The primers used for junction PCR were the pair 5'-CCGAATTGCCGTATTCACCGTTC-3' (complementary to a region upstream of the *chtnA* gene) and 5'-CGCACTGAGAAGCCCTTAG-3' (complementary to a region downstream of the MCS site in pSF100); and the pair 5'-GATTGGGCCGGCTGAAGTTC-3' (complementary to a region downstream of the homology region from the *chtnA* gene) and 5'-GCGATTCAGGCCTGGTATG-3' (complementary to a region upstream of the MCS site in pSF100).

**Comparative metabolic profiling and UHPL-ESI-Q-TOF-MS(/MS) analyses**

To investigate the effect of the insertional mutagenesis on the metabolite profile of *C. koreensis*, WT and mutant *C. koreensis* cultures were streaked on Basal Salts Medium (BSM) ($K_2HPO_4.3H_2O$ 4.25 g/L, $NaH_2PO4.H_2O$ 1 g/L, $NH_4Cl$ 2 g/L, $MgSO_4.7H_2O$ 0.2 g/L, $FeSO_4.7H_2O$ 0.012 g/L, $MnSO_4.H_2O$ 0.003 g/L, $ZnSO_4.7H_2O$ 0.003 g/L, $CoSO_4.7H_2O$ 0.001 g/L, nitrilotriacetic acid 0.1 g/L, casamino acids 0.5 g/L, yeast extract 0.5 g/L) agar plates supplemented with 4 g/L glucose.[13] Following incubation for four days at 28°C, the agar-grown cultures were extracted with ethyl acetate for 1 hour under minimal light exposure. The resulting extracts were dried by rotary evaporation *in vacuo*. The dried extracts were then resuspended in methanol, centrifuged for 1 min at 13,200 rpm and analyzed via UHPLC-ESI-Q-TOF-MS.

UHPLC-ESI-Q-TOF-MS analyses were performed using a Dionex UltiMate 3000 UHPLC coupled to a Zorbax RRHP Eclipse Plus C18 column (2.1x100 mm, 1.8 µL) connected to a Bruker Impact II mass spectrometer. Mobile phases consisted of water (A) and acetonitrile (B), each supplemented with 0.1% formic acid. The following gradient was used at a flow rate of 0.200 mL/min: 0-2.5 min 5% B, 2.5-14 min 5-100% B, 14-19 min 100% B, 19-20.4 min 100-5% B, 20.4-25 min 5% B. The mass spectrometer was operated in positive ion mode with a scan range of 50-3000 m/z. Source conditions were: end plate offset at −500 V; capillary at −4500 V; nebulizer gas ($N_2$) at 1.6 bar; dry gas ($N_2$) at 8 L min−1; dry temperature at 180 °C. Ion transfer conditions were: ion funnel RF at 200 Vpp; multiple RF at 200 Vpp; quadrupole low mass at 55 m/z; collision energy at 5.0 eV; collision RF at 600 Vpp; ion cooler RF at 50–350 Vpp; transfer time at 121 µs; pre-pulse storage time at 1 µs. Calibration was performed with 1 mM sodium formate through a loop injection of 20 µL at the start of each run. Mass spectra were analysed using the Compass Data Analysis software (Bruker).

High-resolution LC-ESI-MS/MS analyses were performed using a Dionex UltiMate 3000 UHPLC coupled to a Zorbax RRHP Eclipse Plus C18 column (2.1x100 mm, 1.8 µL) connected to a Bruker Impact II mass spectrometer. Mobile phases consisted of water (A) and acetonitrile (B), each supplemented with 0.1% formic acid. The following gradient was used at a flow rate of 0.200 mL/min: 0-2.5 min 5% B, 2.5-14 min 5-100% B, 14-19 min 100% B, 19-20.4 min 100-5% B, 20.4-25 min 5% B. The mass spectrometer was operated in positive ion mode using the following parameters: scan range: 50-1500 m/z, nanospray voltage: 3.5 kV, source temperature: 200°C, normalized collision energy: 10 eV-20-30-40, isolation window: ± 8 Da. The lock mass 150.0000 was used as an internal calibrant. The source conditions were: end plate offset at −500 V; capillary at −3500 V; nebulizer gas ($N_2$) at 40 psi; dry gas ($N_2$) at 8 L min−1; dry temperature at 200 °C. Ion transfer conditions were: ion funnel RF at 350 Vpp; multiple RF at 350 Vpp; quadrupole low mass at 150 m/z; collision energy at 5.0 eV; collision RF at 1500 Vpp; ion cooler RF at 50–350 Vpp; transfer time at 80 µs; pre-pulse storage time at 10 µs. Calibration was performed with 1 mM sodium formate through a loop injection of 20 µL at the start of each run. Mass spectra were analysed using the Compass HyStar software (Bruker).

## Isolation and structure elucidation of the chitinimines

For chitinimine production, *C. koreensis* DSM 17726 was grown on BSM agar plates supplemented with 4 g/L glucose. Following incubation for four days at 28°C, the cells and the agar were extracted with ethyl acetate under minimal light exposure. Extracts were dried by rotary evaporation *in vacuo*, and the resulting solids were resuspended in 50% acetonitrile in water. The chitinimine-containing extract was then fractionated by preparative HPLC on a Shimadzu Nexera Prep instrument equipped with a Shimadzu Shim pack GIS column (5 µm, C18, 100 Å, 250 × 10 mm), monitoring absorbance at 190 nm. Mobile phases consisted of water (A) and acetonitrile (B), each supplemented with 0.1% formic acid. The following gradient was used at a flow rate of 5 mL/min: 0–2.5 min 5% B, 2.5–30 min 5-100% B, 30-35 min 100% B, 35-36 min 100-5% B, 36-38 min 5% B. Chitinimine-containing fractions were identified via UHPLC-ESI-Q-TOF analysis. These fractions were concentrated and lyophilized prior to further characterization. The structure of the chitinimines was elucidated using a combination of UHPLC-ESI-Q-TOF-MS and 1- and 2-D NMR experiments.

For NMR spectroscopic analyses, each sample was separately dissolved in 550 µL of DMSO-d6. All spectra were measured on a Bruker Neo spectrometer operating at 600Mhz with quadruple cryoprobe ($^{1}$H,$^{31}$P,$^{15}$N,$^{13}$C) and processed with Topspin software. The 2D DQF-COSY[14],TOCSY[15], and NOESY[16] spectra were recorded with a sweep width of 6600 Hz in both dimensions. The total TOCSY mixing time was set to 62 ms. Zero-quantum interference in TOCSY spectra was eliminated by gradients.[14] Homonuclear spectra were acquired with 8 to 72 scans depending on the sample concentration, 4096 data points in ($t_2$) and 512 to 1024 FIDs in ($t_1$). The data were apodized with a shifted sine-bell square function in both dimensions and processed to a 4K × 1K matrix. The NOESY experiments were acquired with mixing time 200 ms. Natural abundance [$^{1}$H, $^{13}$C]-HSQC[17] were recorded with sensitivity enhancement and gradient coherence selection optimized for multiplicity editing with negative signals for $CH_2$ moieties and positive signals for CH and $CH_3$ groups ($^{1}J_{CH}$ = 145 Hz) using 8 to 32 scans (depending on sample concentration) and 1K/4K complex data points and 150/11 ppm spectral widths in $t_1$ and $t_2$, respectively. For the most concentrated sample (chitinimine I/III) a 2D HSQC-TOCSY was also obtained using an HSQC building block followed by a clean MLEV17 TOCSY transfer step with 80 ms mixing time.[18] The spectrum was recorded using 40 scans and 512/4K complex data points and 150/11 ppm spectral widths in $t_1$ and $t_2$, respectively. Natural abundance [$^{1}$H, $^{13}$C]-HMBC were measured with 64 scans and 4096/512 complex data points and 11/230 ppm spectral widths in $t_2$ and $t_1$, respectively ($^{1}J_{CH}$ = 145 Hz and $^{3}J_{CH}$ = 4 Hz).

Coupling constants (*J* in Hz) for the peptide ring were reported based on the 1- and 2-D NMR spectra where possible. Mostly, cross-peaks of the phase-sensitive DQF-COSY experiments were used to distinguish the strength of the active and passive couplings in the F2 dimension. This is reasonable since the spectral resolution of the COSY experiments (1.6 Hz with 4k points in F2) was high enough compared to most of the *J*-couplings between nascent protons in the chitinimines (> 5 Hz), therefore separating the antiphase Lorentzians in the cross peaks. The multiplicity of the carbons in the chitinimine molecules was reported based on the multiplicity-edited HSQC recorded on all samples. Cross peaks with a positive phase correspond to CH/$CH_3$ moieties, while negatively phased cross peaks show $CH_2$ moieties. Extractions of the coupling constants for the multiplets in the peptide ring was unsuccessful.

## Determination of absolute stereochemical configurations using Marfey's method

The absolute stereochemistry of the amino acid constituents of the chitinimines was determined using Marfey's method. The chitinimines were subjected to hydrolysis with 1 M HCl for 24 hours at 100°C, and subsequently derivatized with 1-fluoro-2-4-dinitrophenyl-5-L-alanine amide (FDAA) as described by Tanino *et al.* (2010). The resulting diastereomers were compared to Marfey's derivatives of the appropriate D- and L-amino acid standards by UHPLC-ESI-Q-TOF-MS.[19] All amino acid standards were commercially available, except for (3*S*,4*S*)-4-amino-3-hydroxypentanoic acid and (3*R*,4*R*)-4-amino-3-hydroxypentanoic acid, which were acquired with an FMOC protecting group. For deprotection, 50 mg of FMOC-(3*S*,4*S*)-4-amino-3-hydroxy-pentanoic acid or (3*R*,4*R*)-4-amino-3-hydroxypentanoic acid were dissolved in a water-DMF mixture to a final concentration of 0.1 M, followed by reaction with excess piperidine (5.0 equivalents) in the presence of DCM. This reaction was allowed to proceed overnight at room temperature. The mixture was then dried *in vacuo*, washed three times with water and dissolved in methanol for UHPLC-ESI-Q-TOF-MS analysis to confirm successful deprotection.

The authentic standards were prepared by mixing 1.0 equivalent of each amino acid, dissolved in 1 M $NaHCO_3$, with 2.0 equivalents of FDAA dissolved in acetone. The mixtures were incubated for 2 hours at 40°C, followed by quenching with 1 M HCl. The solvents were removed under reduced pressure, and the resulting residues were dissolved in DMSO and diluted 10 times with methanol. The UHPLC-ESI-Q-TOF-MS analyses were performed using a Pursuit XRs 100Å Diphenyl HPLC column (2.0 x 150 mm, 3 µm) connected to a Bruker Impact II mass spectrometer (with the same parameters as described above). The following gradient at a flow rate of 0.200 mL/min was applied: 0–2.5 min 5% B, 2.5–7 min 5-35% B, 7-40 min 35% B, 40-44.5 min 35-100% B, 44.5-45.5 min 100-5% B, 45.5–50 min 5% B.

## Acid hydrolysis of the chitinimines

For acid hydrolysis, 4 mg of sample containing a mixture of chitinimine I/III and chitinimine II at a ratio of 8:1 was dissolved in 400 µL of DCM and 100 µL of water. 100 µL of TFA was added dropwise, and the resulting mixture stirred at room temperature for 24 hours. Solvents were removed under reduced pressure and the resulting product was washed twice with 100 µL of DCM. The hydrolysed chitinimines were dissolved in 500 µL of methanol and analyzed with UHPLC-ESI-Q-TOF-MS.

## DFT calculations

Quantum chemical calculations were carried out on the DFT level using the ORCA package (version 6.0.1).[20–32] CREST (3.0.2)[33] was used for ensemble generation and CENSO (2.1.3)[34] was used for finetuning of the conformational ensembles. GFN2-xTB (xTB version 6.7.1)[35,36] was used within the CREST and CENSO packages for fast DFT calculations, each time with ALPB(DMSO) solvation.[37] D4 corrections[27,38] and CPCM(DMSO) implicit solvation[39] were used in ORCA for geometry optimizations and shielding calculations. Frequency analyses confirmed the presence of an energetic minimum. Molecular 3D renderings were made with the Chemcraft software.[40]

The goal of the DFT calculations was to assign the stereoconfiguration of the lipid's C3 carbon by finding the best-fit approximation to the NMR chemical shifts. From the Marfey's analysis, it was found that both D-Ile and L-Ile were present in chitinimine I, and therefore, the $\alpha$-carbon stereocenter is assumed to exist as a racemic 50:50 mixture for the DFT calculations. Four stereoisomers are thus possible (named (*S*,*S*), (*R*,*S*), (*S*,*R*) and (*R*,*R*)), and all four were submitted to the calculations (**Figure S31A**). The target values to compare experimental vs. predicted are the chemical shifts of the C3 carbon and the H atom attached to it (45.7 and 3.80 ppm experimentally in DMSO-d6 at 600 MHz).

**Conformational search**: OpenBabel (v3.1.0) was used to generate the initial geometries. CREST was then used to generate a conformational ensemble (several hundred conformers were typically found). This ensemble was then submitted to CENSO and sorted in the four-part workflow, using the default settings for CENSO 2.1.3: [prescreening]: threshold=4.0 kcal/mol, PBE-D4/def2-SVP [screening]: threshold=3.5 kcal/mol, r²SCAN-3c [optimization]: threshold=3.0 kcal/mol, r²SCAN-3c [refinement]: threshold 0.9 (Boltzmann population cutoff), ωB97X-V/def2-TZVP [general]: solvent=dmso. This sorting resulted in the following lowest-energy conformers within the 90% cutoff (Boltzmann population in parentheses):

(*S*,*S*): CONF4 (81%), CONF5 (19%)

(*R*,*S*): single conformer

(*S*,*R*): CONF24 (90%), CONF26 (10%)

(*R*,*R*): single conformer

**$^1$H NMR**: Conformers obtained after the CENSO run were re-optimized on the ωB97X-D4/def2-TZVP level. Subsequently, the NMR shielding constants were calculated on the WP04/6-311+G(3df) level.[41,42] Linear regression was then applied to obtain the fitted chemical shift values, and the linear scaling factors for this level of theory were determined prior by fitting experimental data from the DELTA50 benchmark.[43] The equation used was

$$\delta = -0.9938\times\sigma + 32.147$$

with δ the fitted chemical shift and σ the calculated NMR shielding constant. Below is the compound script used for the combined opt+NMR calculation:

```
# -------------------------------------------------------------------
New_Step
! wB97X-D4 def2-TZVP def2/J TightSCF TightOpt Freq CPCM(DMSO) miniprint
Step_end
# -------------------------------------------------------------------

#read in .xyz and .gbw from previous job
Read_geom(1);
ReadMOs(1);

# -------------------------------------------------------------------
New_Step
```

```
! RIJCOSX 6-311+G(3df) AutoAux defgrid3 TightSCF CPCM(DMSO) NoFrozenCore NMR
        %method
        method dft
        functional hyb_gga_xc_wp04
        end
Step_end
# -------------------------------------------------------------------------
End
```

**$^{13}$C NMR**: Conformers obtained after the CENSO run were re-optimized on the PBE0/def2-TZVP level. Subsequently, the NMR shielding constants were calculated on the ωB97X-D4rev/6-31G(d,p) level. Linear regression was then applied to obtain the fitted chemical shift values, and the linear scaling factors for this level of theory were determined prior by fitting experimental data from a subset of the DELTA50 benchmark.[43] The equation used was

$$\delta = -1.0343\times\sigma + 206.16$$

with δ the fitted chemical shift and σ the calculated NMR shielding constant. Below is the compound script used for the combined opt+NMR calculation:

```
# -------------------------------------------------------------------------
New_Step
! PBE0 D4 def2-TZVP def2/J TightSCF TightOpt Freq CPCM(DMSO) smallprint
Step_end
# -------------------------------------------------------------------------

#read in .xyz and .gbw from previous job
Read_geom(1);
ReadMOs(1);

# -------------------------------------------------------------------------
New_Step
! wB97X-D4rev 6-31G(d,p) defgrid3 TightSCF CPCM(DMSO) NoFrozenCore NMR
Step_end
# -------------------------------------------------------------------------
End
```

**Analysis**: The calculated chemical shift values were then compared to the experimental values (**Table S8**). Stereoisomers *RS* and *RR* each produced a single value, stereoisomers *SS* and *RS* each consisted of two conformers, so the weighted average was used keeping into account the Boltzmann population mentioned above. Lastly, since the Ile α-carbon is assumed to be racemic, we use the arithmetic mean of *SS* + *SR* on the one hand, and *RS* + *RR* on the other, as the final values to compare with the experimental shifts. From this, the *S* stereocenter appears to be closer to reality, both on the $^{1}$H NMR level as well as on the $^{13}$C NMR level (**Table S8**, **Figure S31B**). For this reason, the stereochemistry of the C3 of the specialized lipid is tentatively assigned as *S*.

### Phylogenetic and bioinformatic analysis of the chitinimine biosynthetic pathway

The hybrid PKS-NRPS-PUFA synthase-like biosynthetic gene cluster from *C. koreensis* was analyzed in-depth using antiSMASH version 7.1.0, with all optional parameters for extended analysis enabled. The domain organization of the FAS, PKS and NRPS subunits was determined through InterPro protein classification and NCBI's Conserved Domain Database (CDD) searches. Putative functions were assigned to the proteins encoded by each gene using a combination of NCBI BlastP and UniProt BLAST. Proteins belonging to the same enzyme class were subjected to Clustal Omega (EMBL-EBI) and the resulting multiple sequence alignment were examined for conserved residues indicative of catalytic activity and stereochemical or substrate specificity. The amino acid substrate selectivity of the adenylation domains was predicted using antiSMASH and PARAS.[44] Condensation domain sequences were extracted and analyzed via the Natural Product Domain Seeker (version 2) (NaPDoS2).[45] Specifically, these sequences were aligned against 172 reference condensation domains from the NaPDoS2 database (**Table S10**), and the resulting neighbor-joining phylogenetic tree was visualized with iTOL.

## Bioactivity assays

The antimicrobial activity of the chitinimines was tested against the bacterial and fungal strains listed in **Table S14**.

Soft agar halo assays were carried out to compare the antibacterial activity of WT and mutant *C. koreensis* strains. 10 µL of stationary-phase cultures of *C. koreensis* WT and mutant strains were spotted onto BSM agar plates supplemented with glucose (4 g/L). Following incubation for four days at 28°C, stationary-phase cultures of the target strains were diluted 1:1000 in 15 mL of soft agar prepared in their preferred growth medium and poured over the surface of the BSM plates. After incubation for 24 hours at the optimal growth temperature of the target strains (**Table S14**), the presence and size of the inhibition zones was evaluated. If growth-promoting effects were observed instead of inhibition, the assay was repeated with the addition of iodonitrotetrazolium chloride (0.2 mg/mL) to the soft agar for improved visualization of bacterial growth.

To chemically complement the Δ*chtnA* strain and therefore recover the growth-promotion effect observed against *Salmonella enterica* serovar Typhimurium ATCC 14028, *Salmonella enterica* serovar Enteritidis ATCC 13046 and *Salmonella enterica* serovar Newport C487, the soft agar halo assays were carried out as indicated above with the sole difference being the supplement of 5 µl of chitinimine I/III and 5 µl of chitinimine II (dissolved in DMSO) at final concentrations of 5 mg/ml to spots containing either the Δ*chtnA* or WT strains. Furthermore, 10 µl of a concentrated ethyl acetate extract from WT *C. koreensis* (obtained as indicated in the above section 'Isolation and structure elucidation of the chitinimines') dissolved in a 1:1 milli-Q acetonitrile mixture were also supplemented in the same fashion. Appropiate solvent controls, with either DMSO or milli-Q acetronitrile, were added to the cells.

To further verify the growth promotion effect, WT and KO *C. koreensis* cultures were spotted onto BSM agar plates supplemented with glucose as a sole carbon source. Following incubation for four days at 28°C, cultures of *Salmonella enterica* serovar Typhimurium ATCC 14028, *Salmonella enterica* serovar Enteritidis ATCC 13046, or *Salmonella enterica* serovar Newport C487 were spotted on the same plates at variable distances. After 48 hours of incubation at 37°C, the growth of *Salmonella* species at different distances from WT and KO *C. koreensis* strains was evaluated.

The bioactivity of the purified chitinimines was also determined via plate lawn assays. A mixed sample containing both chitinimine I/III and II at a ratio of 10:1, respectively, and at a final concentration of 1 mg/mL (dissolved in DMSO) was tested. Agar plates were overlaid with lawns of stationary-phase cultures of target bacterial strains, diluted to an $OD_{600}$ of 0.03. 10 µL of the chitinimine mixture was spotted onto sterile filter paper placed on top of the lawns. DMSO was used as a negative control. Plates were incubated for 24 hours under the preferred growth conditions for each target strain. Presence and size of inhibition halos were evaluated.

To assess the growth-promoting effect of purified chitinimines, *Salmonella enterica* serovar Typhimurium ATCC 14028, *Salmonella enterica* serovar Enteritidis ATCC 13046 and *Salmonella enterica* serovar Newport C487 were cultured in the presence of the compounds and growth was monitored at $OD_{600}$. In a sterile 96-well microtiter plate, 2.5 µL of chitinimine I/III or chitinimine II (dissolved in DMSO) was added at a final concentration of 125 µg/mL to 97.5 µL of bacterial suspension ($10^6$ CFU/mL in LB broth). In control experiments, DMSO was added instead of the chitinimines, and non-inoculated LB medium was used to subtract background signal. The $OD_{600}$ was monitored every 15 minutes for 13 hours at 37°C in a CLARIOstar Plus (BMG LABTECH), and in between reads, cells were shaken at 300 rpm. The resulting growth curves were visualized with QurvE.[46]

Minimal inhibitory concentration (MIC) values for the chitinimines were determined by the broth microdilution method, following the guidelines from the Clinical Laboratory Standards Institute (CLSI) (document M07, 12th edition).[47] In a 96-well microtiter plate, 50 µL of serial twofold dilutions of the metabolites in Mueller Hinton (M-H) broth were mixed with 50 µL of bacterial suspension ($10^6$ CFU/mL in M-H broth). After incubation 18 hours at the preferred growth temperature of the target bacteria, MIC values were defined as the lowest concentrations that visibly inhibited bacterial growth.

For antifungal assays, pre-warmed RPMI-MOPS medium, supplemented with 0.2% glucose, was inoculated with fungal pathogen cultures (see **Table S14**) that had been washed twice with Phosphate Buffered Saline (PBS). The final inoculum was adjusted to an optical density at 600 nm ($OD_{600}$) of 0.001. In a sterile 96-well microtiter plate, wells were filled with 196 µL of the inoculated medium and 4 µL of either chitinimine I/III or chitinimine II at a concentration of 5 mg/mL (dissolved in DMSO). Plates were incubated for 24 hours at 37 °C for *Candidozyma auris* (formerly *Candida auris*) and *Nakaseomyces glabratus* (formerly *Candida glabrata*) strains, or at 30 °C for *Candida albicans* strains. Following incubation, the $OD_{600}$ was measured using a Synergy H1 plate reader (BioTek). To establish the background signal, a blank consisting of non-inoculated RPMI-MOPS was included. Wells supplemented with DMSO instead of compound served as growth controls for each pathogen. The percentage of growth inhibition relative to the control was calculated using the following formula:

$$\text{Inhibition (\%)} = \left(1 - \frac{ODsample - ODblank}{ODcontrol - ODblank}\right) * 100$$

The cytotoxicity of the chitinimines on mammalian cells was assessed using the CyQUANT™ LDH Cytotoxicity Assay Kit (Invitrogen, Thermo Fisher Scientific; Cat. No. C20300) according to the manufacturer's instructions. HeLa (ATCC CCL-2) and CaCo-2 (ATCC HTB-37) cells were used for this assay. Cultures were maintained and incubated during cytotoxicity determination at 37 °C in a humidified 5% $CO_2$ atmosphere. One day prior to compound exposure, cells were seeded in Nunclon Delta Surface 96-well plates (Thermo Fisher Scientific) at a density of 1 × $10^5$ cells/mL, with 100 µL of cell suspension per well. Cytotoxicity measurements followed the CyQUANT™ LDH protocol. 2 µL of chitinimine I/III or chitinimine II at an initial concentration of 5 mg/mL (dissolved in DMSO) and 8 µL of MilliQ-water were added to the wells. The compounds were substituted with DMSO for cell growth control. Wells containing untreated cells served as background controls, while maximum LDH release controls were obtained by lysing cells with the supplied lysis buffer. Exposure of the cells to the compound lasted 45 min until addition of reaction substrate. Absorbance of the resulting formazan product was measured at 490 nm and 680 nm spectrophotometrically, and percent cytotoxicity was calculated following the manufacturer's formula:

$$\text{Cytotoxicity (\%)} = \frac{\text{(Experimental LDH release - Spontaneous LDH release)}}{\text{(Maximum LDH release - Spontaneous LDH release)}} * 100$$

For surfactant activity testing, a microtiter assay adapted from the method by Vaux[48], and described by Walter *et al.*[49], was employed. In a 96-well microtiter plate, 25 µL of either chitinimine I/III or chitinimine II (dissolved in DMSO) were added to 75 µL of milli-Q water to obtain a final concentration of 1.25 mg/mL. 100 µL of either milli-Q water, 1% sodium dodecyl sulphate (SDS) solution or 25% DMSO were used as negative, positive and solvent effect controls, respectively. The plate was placed on top of millimeter graph paper sheet, and distortion of the grid was interpreted as an indication of surfactant activity. In addition, the drop-collapse assay from Dose *et al.*[50], and originally described by Jain *et al.*[51], was applied for further confirmation. 10 µL droplets of 25% chitinimine I/III or chitinimine II (dissolved in DMSO) at a final concentration of 1.25 mg/mL and 75% milli-Q water were spotted on top of Parafilm 'M'. 10 µL of either milli-Q water, 1% SDS solution or 25% DMSO were spotted as negative, positive and solvent effect controls, respectively. Collapse of the droplets was interpreted as a positive indication of surfactant activity.

## Results and Discussion

### Supplementary Figures

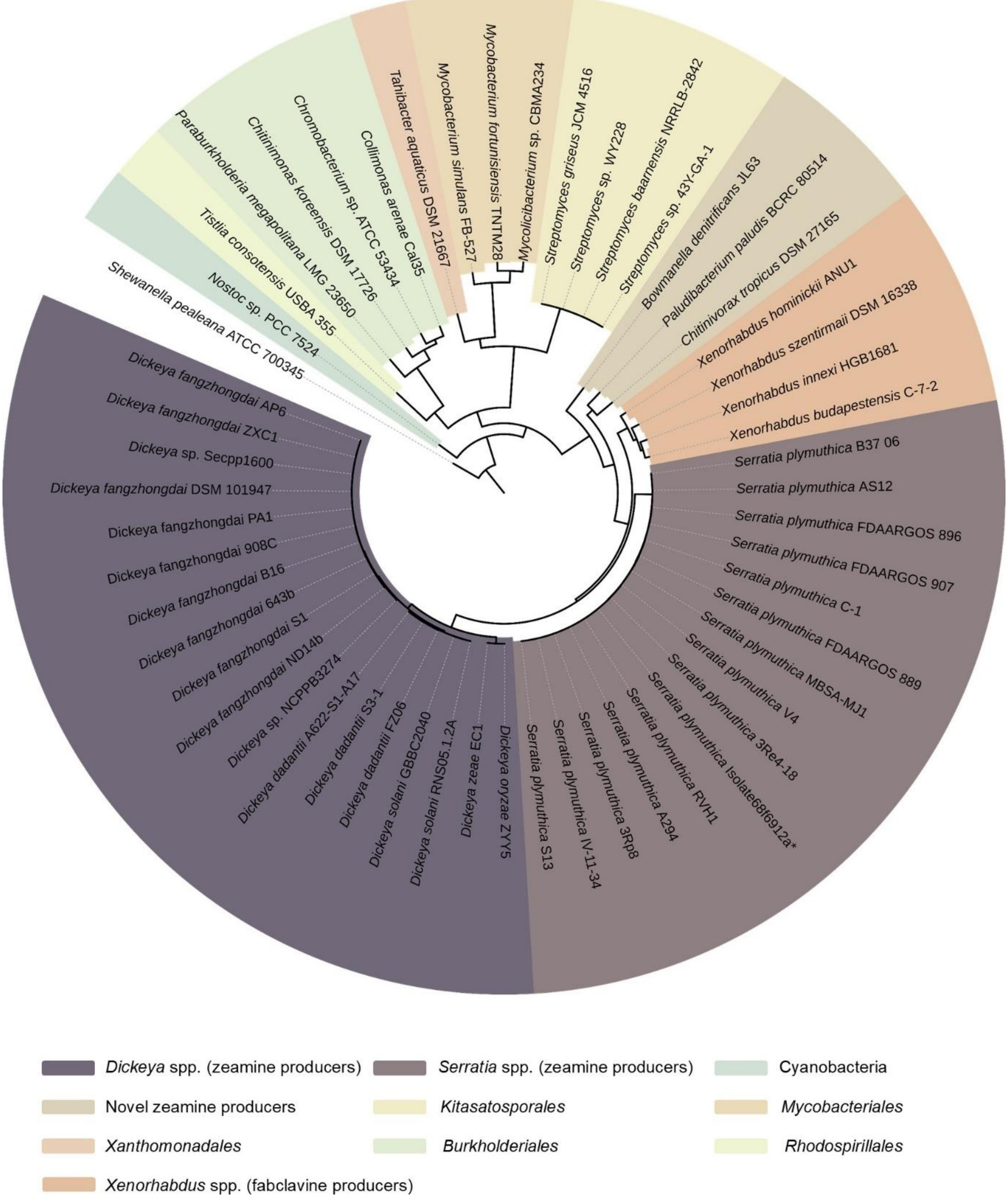


**Figure S1. Phylogenetic distribution of KS-CLF heterodimers from hybrid PKS-NRPS-PUFA synthase-like clusters.** A neighbor-joining tree was constructed from the KS-CLF domain sequences, with the PfaC KS-CLF didomain from the *S. pealeana* PUFA synthase used as the outgroup. Colored clades correspond to related pathways and producing organisms.

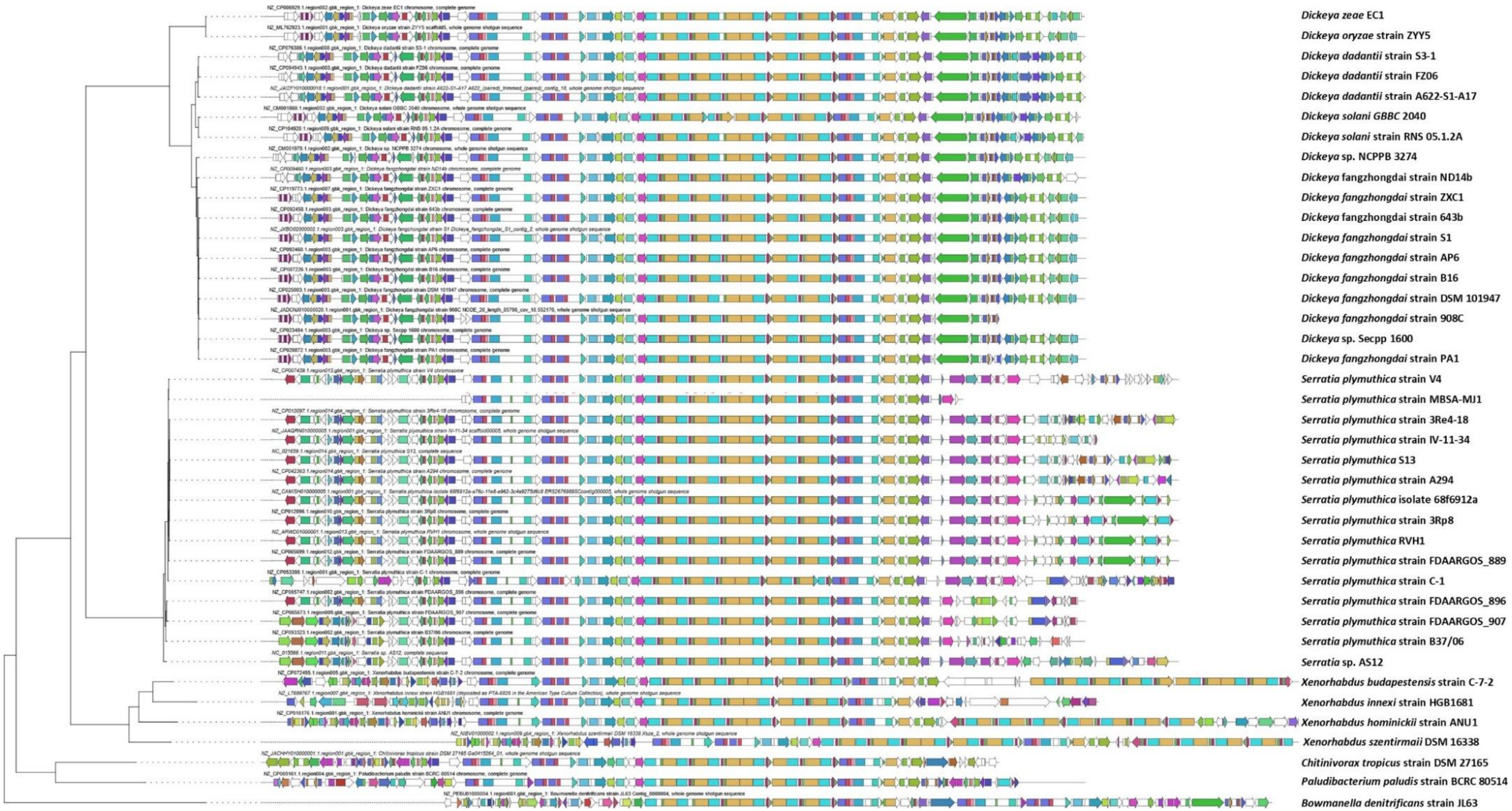


**Figure S2. Phylogenetic distribution and comparative analysis of the zeamine and fabclavine BGCs (clade I) detected using the customized antiSMASH search.** The evolutionary relationships, synteny, and architectural diversity of the BGCs across multiple *Dickeya*, *Serratia* and *Xenorhabdus* species are illustrated. The newly identified zeamine-producing strains *Chitinivorax tropicus* strain DSM 27165, *Paludibacterium paludis* strain BCRC 80514 and *Bowmanella denitrificans* strain JL63 are also included in the analysis. Figure adapted from BiG-SCAPE.[10]

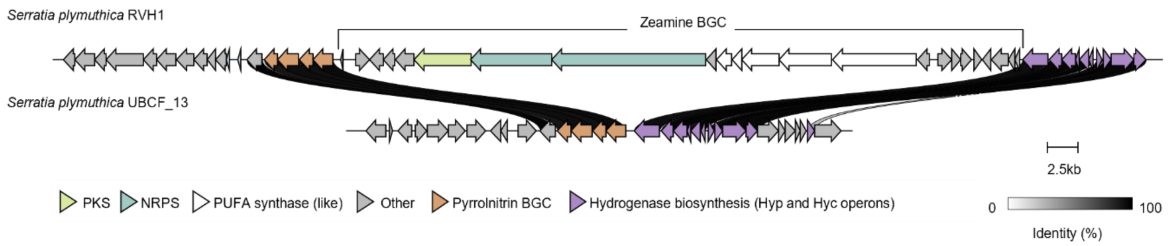


**Figure S3. Genomic comparison of the region surrounding the zeamine BGC in two closely-related *Serratia plymuthica* strains, revealing the genomic integration site of the cluster.** In strain RVH1, the zeamine BGC is located between the pyrrolnitrin BGC (colored in orange) and the hyp and hyc operons involved in hydrogenase biosynthesis (colored in purple). In contrast, strain UBCF_13 contains only these flanking regions, with the zeamine cluster absent, suggesting that this locus serves as the insertion site for the zeamine BGC. Figure adapted from clinker.[11]

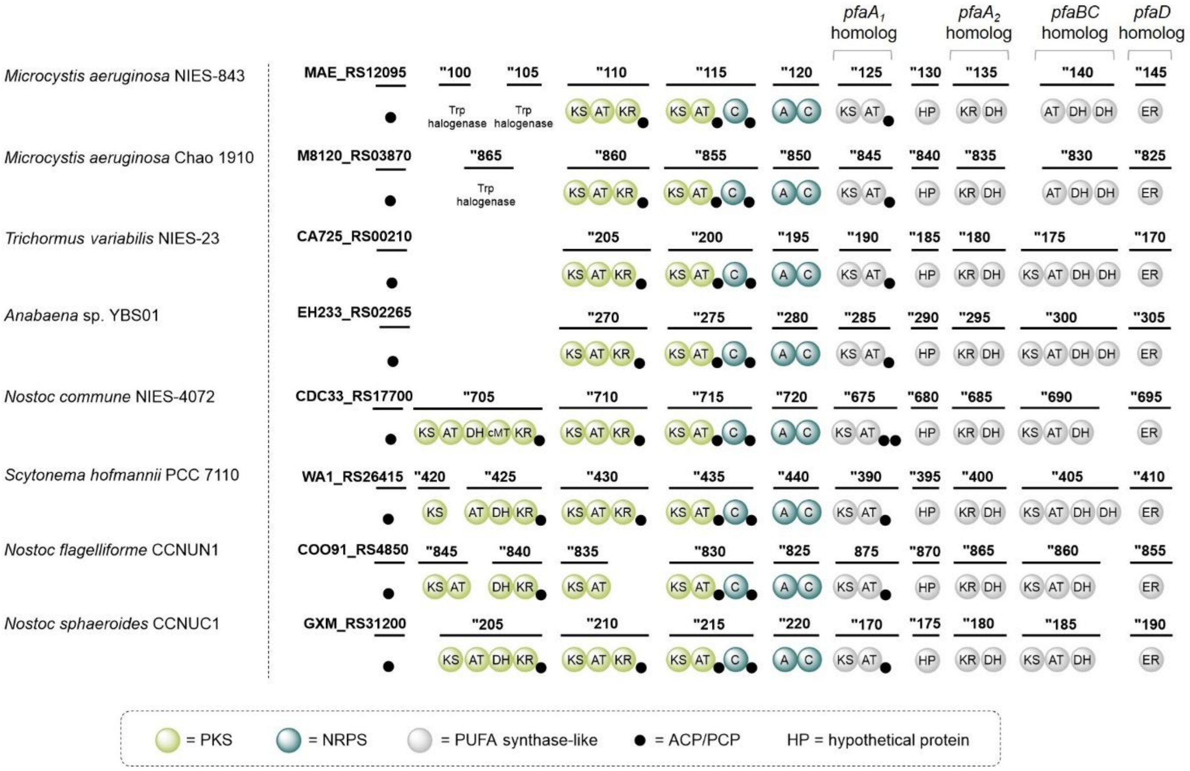


**Figure S4. Comparison of the domain architecture of the hybrid PKS-NRPS-PUFA synthase-like pathways in clade II, comprised of *M. aeruginosa, T. variabilis, Anabaena* sp., *N commune, S. hofmannii, N. flagelliforme* and *N. sphaeroides*.** PKS domains are colored in light green, NRPS domain in dark green, PUFA synthase-like biosynthetic machinery in grey, acyl and peptidyl carrier proteins in black. Putative tryptophan halogenases are indicated and conserved hypothetical proteins with a predicted Rossmann-fold NAD(P)-binding domain are labelled as HP.

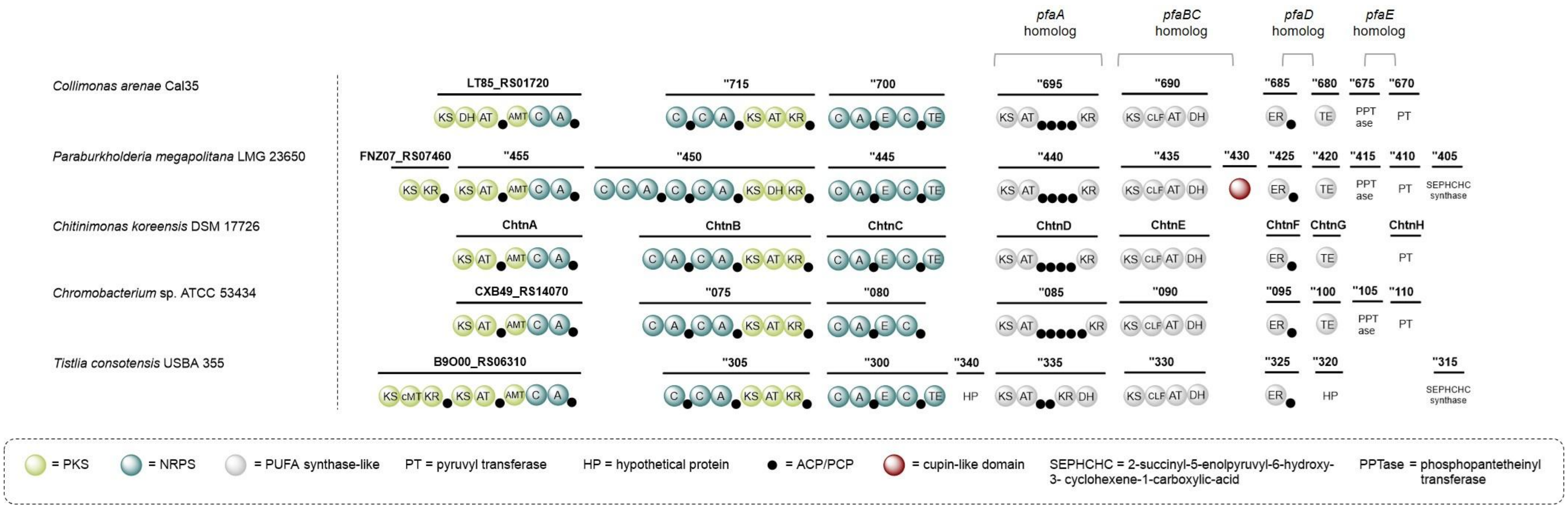


**Figure S5. Comparison of the domain architecture of the hybrid PKS-NRPS-PUFA synthase-like pathways in clade III, found in *C. arenae, P. megapolitana, C. koreensis, Chromobacterium* sp. and *T. consotensis.*** PKS domains are colored in light green, NRPS domains in dark green, PUFA synthase-like biosynthetic machinery in grey, acyl and peptidyl carrier proteins in black and cupin-like domains in brown. Putative tailoring enzymes and other proteins that are conserved across (a subset of) these pathways are shown with abbreviated labels.

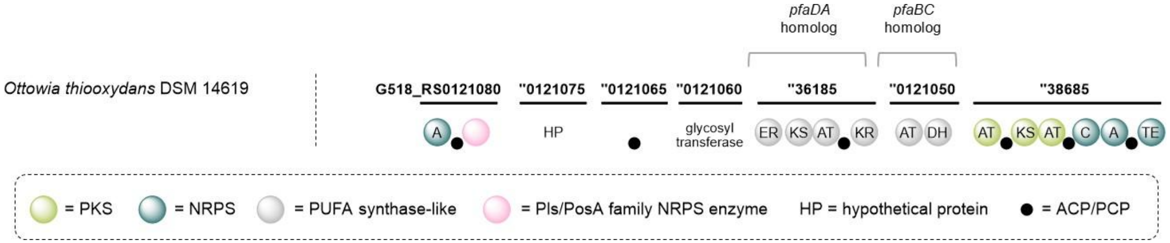


**Figure S6. Domain architecture of the hybrid PKS-NRPS-PUFA synthase-like pathway in *O. thiooxydans.*** PKS domains are colored in light green, NRPS domains in dark green, PUFA synthase-like biosynthetic machinery in grey, acyl and peptidyl carrier proteins in black, putative Pls/PosA family NRPS enzymes in pink, glycosyltransferases in orange and hypothetical proteins in blue-grey.

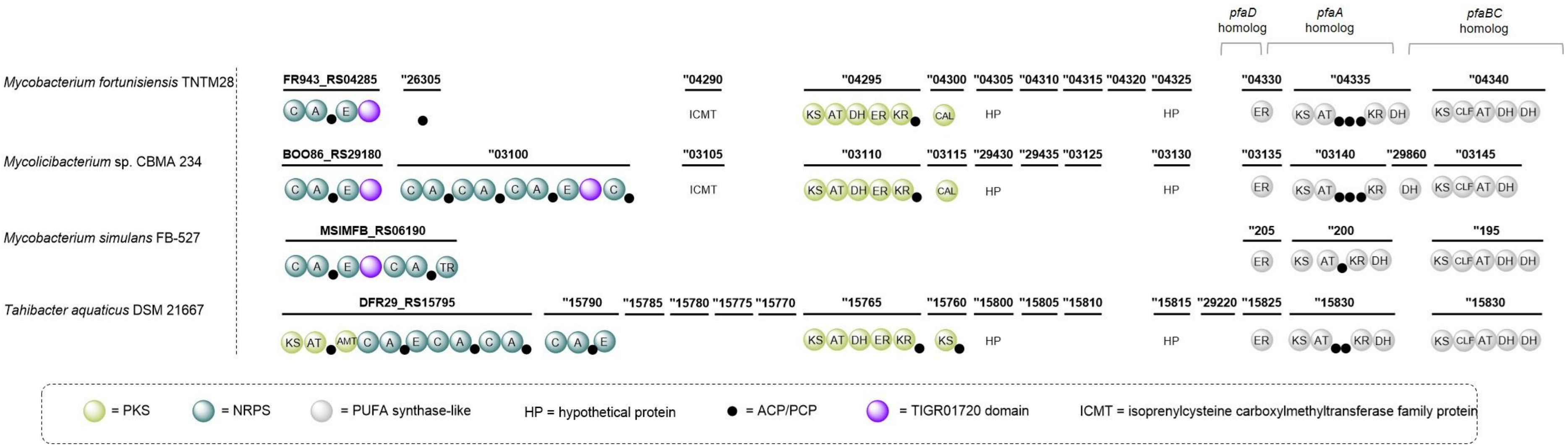


**Figure S7. Comparison of the domain architecture of the hybrid PKS-NRPS-PUFA synthase-like pathways in clade IV, composed of *M. fortunisiensis*, *Mycolicibacterium* sp., *M. simulans* and *T. aquaticus*.** PKS domains are colored in light green, NRPS domains in dark green, PUFA synthase-like biosynthetic machinery in grey, acyl and peptidyl carrier proteins in black and TIGR01720 domains in bright purple. Putative tailoring enzymes and other proteins that are conserved across (a subset of) these pathways are shown with abbreviated labels.

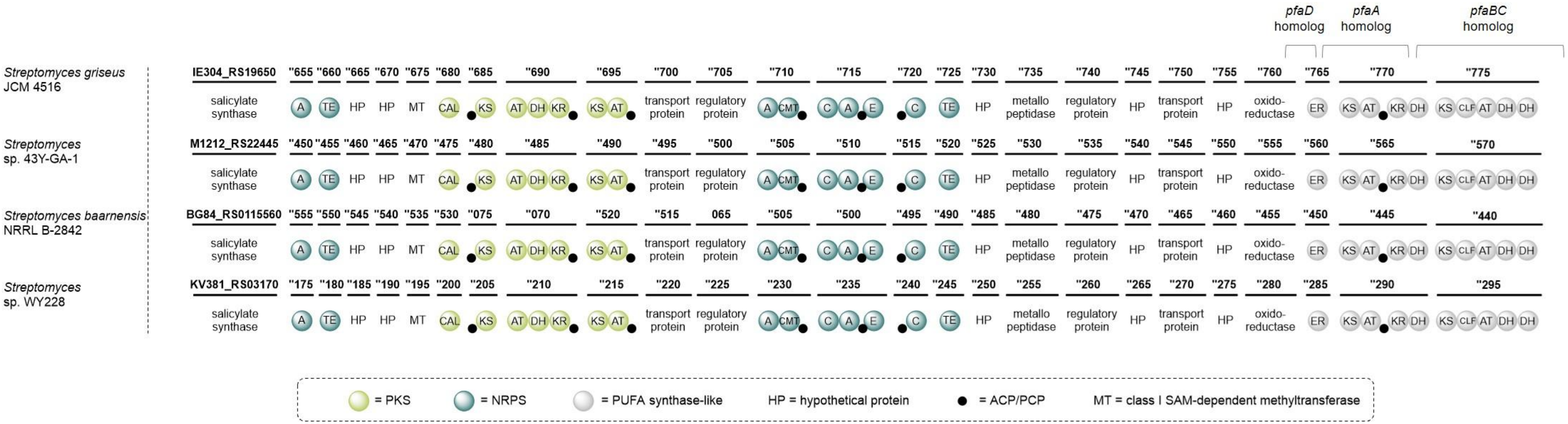


**Figure S8. Comparison of the domain architecture of the hybrid PKS-NRPS-PUFA synthase-like pathway in clade V, which includes *S. griseus*, *Streptomyces*. sp. 43Y-GA-1, *S. baarnensis* and *Streptomyces*. sp. WY228.** PKS domains are colored in light green, NRPS domains in dark green, PUFA synthase-like biosynthetic machinery in grey, and acyl and peptidyl carrier proteins in black. Putative tailoring enzymes and other proteins that are conserved across (a subset of) these pathways are shown with abbreviated labels.

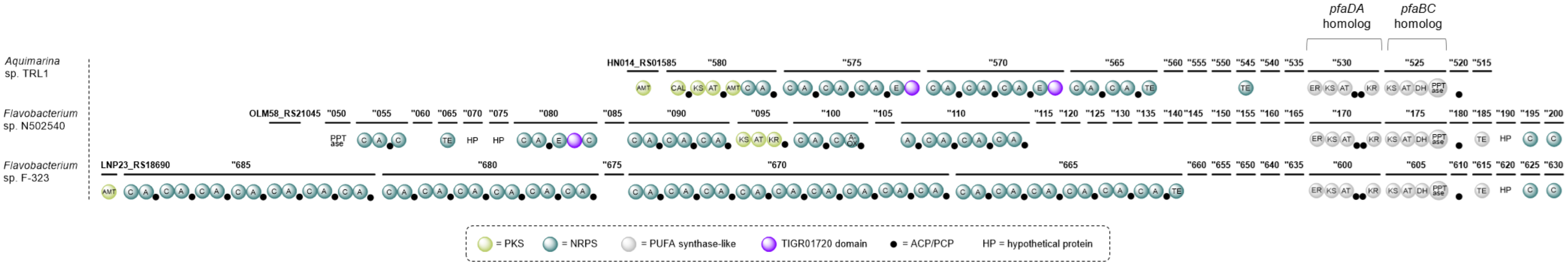


**Figure S9. Comparison of the domain architecture of the hybrid PKS-NRPS-PUFA synthase-like pathways in clade VI, which consists of *Aquimarina* sp., *Flavobacterium* sp. N502540 and *Flavobacterium* sp. F-323.** PKS domains are colored in light green, NRPS domains in dark green, PUFA synthase-like biosynthetic machinery in grey, acyl and peptidyl carrier proteins in black and TIGR01720 domains in bright purple. Putative tailoring enzymes and other proteins that are conserved across (a subset of) these pathways are shown with abbreviated labels.

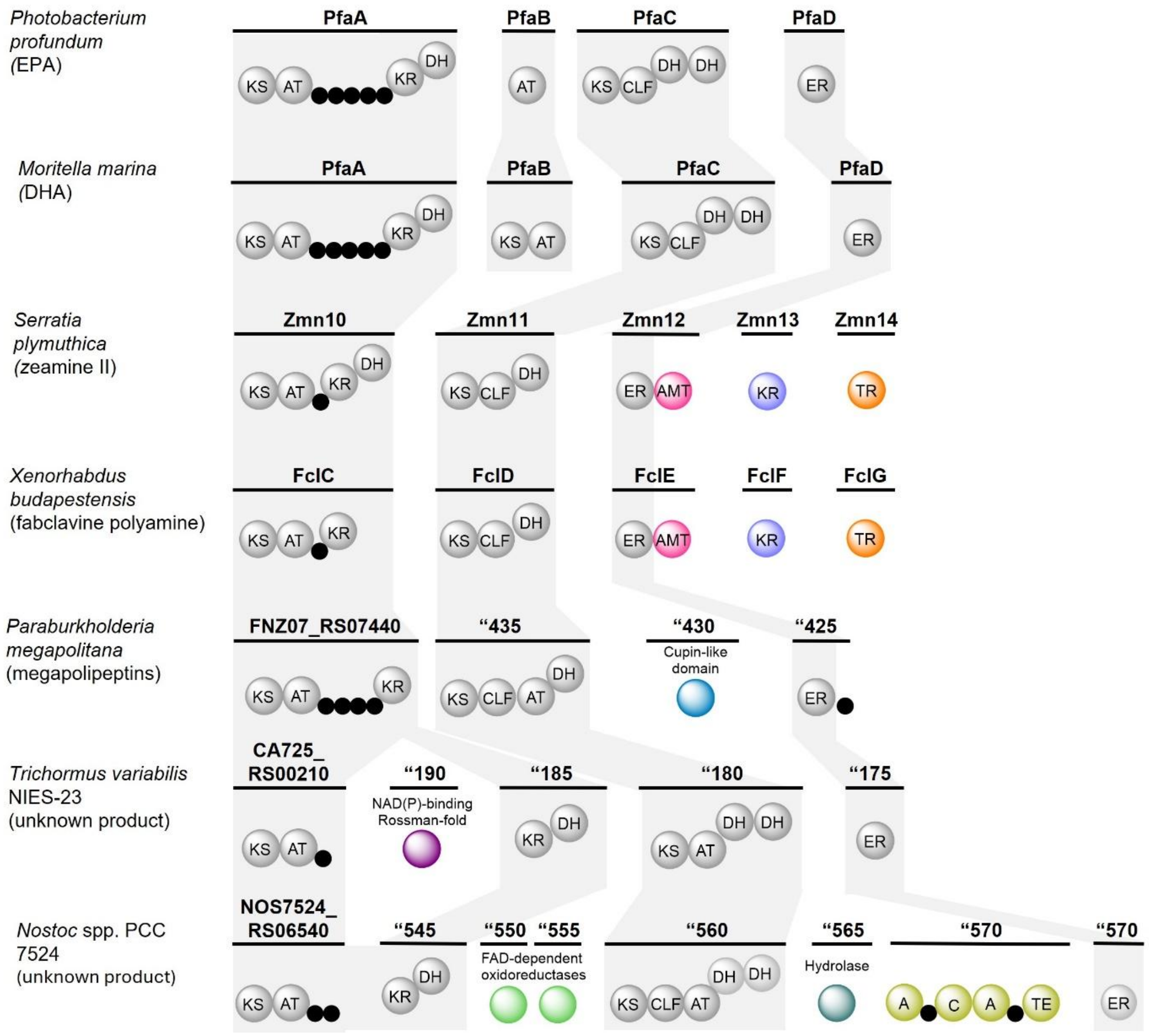


**Figure S10. Unusual catalytic domains in secondary lipid synthases.** Overview of unconventional catalytic domains that have been recruited by PUFA synthases (and homologs) over the course of evolution to diversify the structures of PUFAs.

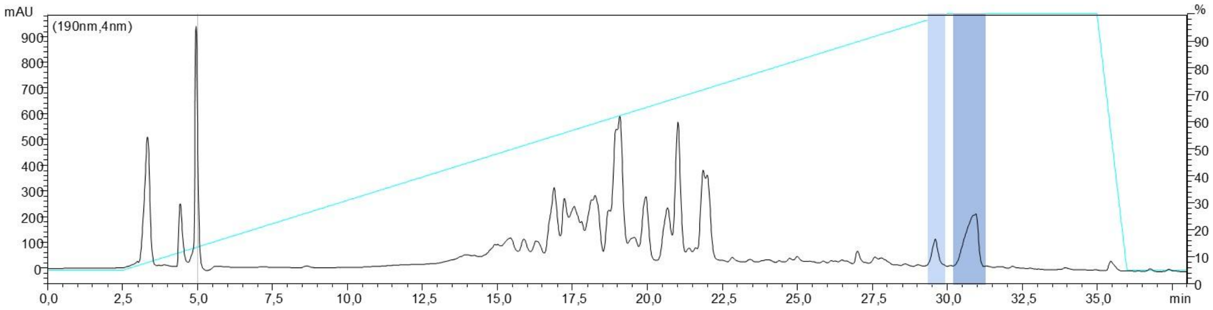


**Figure S11. Large-scale chitinimine production.** UV chromatogram from HPLC analysis (monitoring absorbance at 190 nm) of ethyl acetate extracts of agar plates grown with WT *C. koreensis* DSM 17726. The peaks that correspond to chitnimine II and chitinimine I/III are indicated with a light and dark blue box, respectively. The applied solvent gradient is shown as a blue line. The percentage of acetonitrile in water is indicated on the right y-axis.

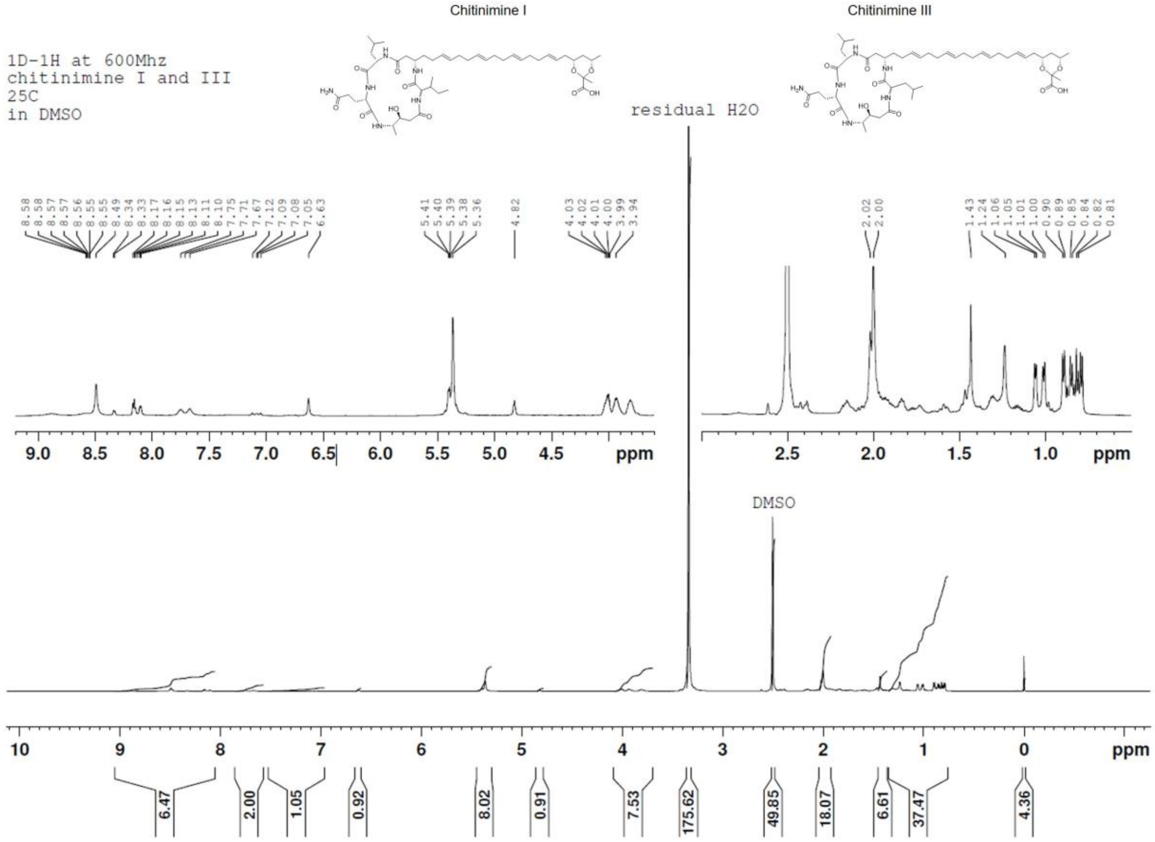


**Figure S12.** $^{1}$H NMR spectrum of chitinimine I and III in DMSO-d6.

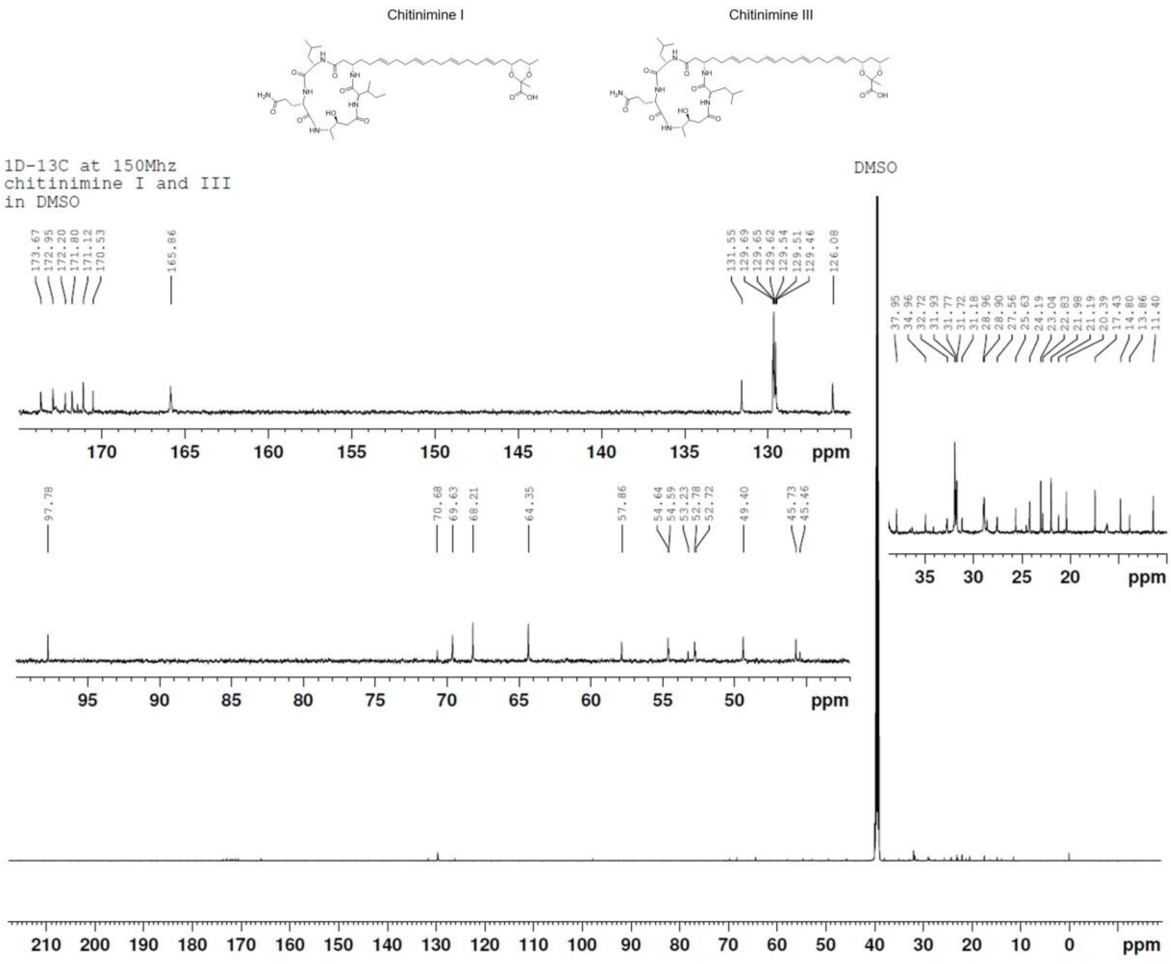


**Figure S13**. $^{13}C$ NMR spectrum of chitinimine I and III in DMSO-d6.

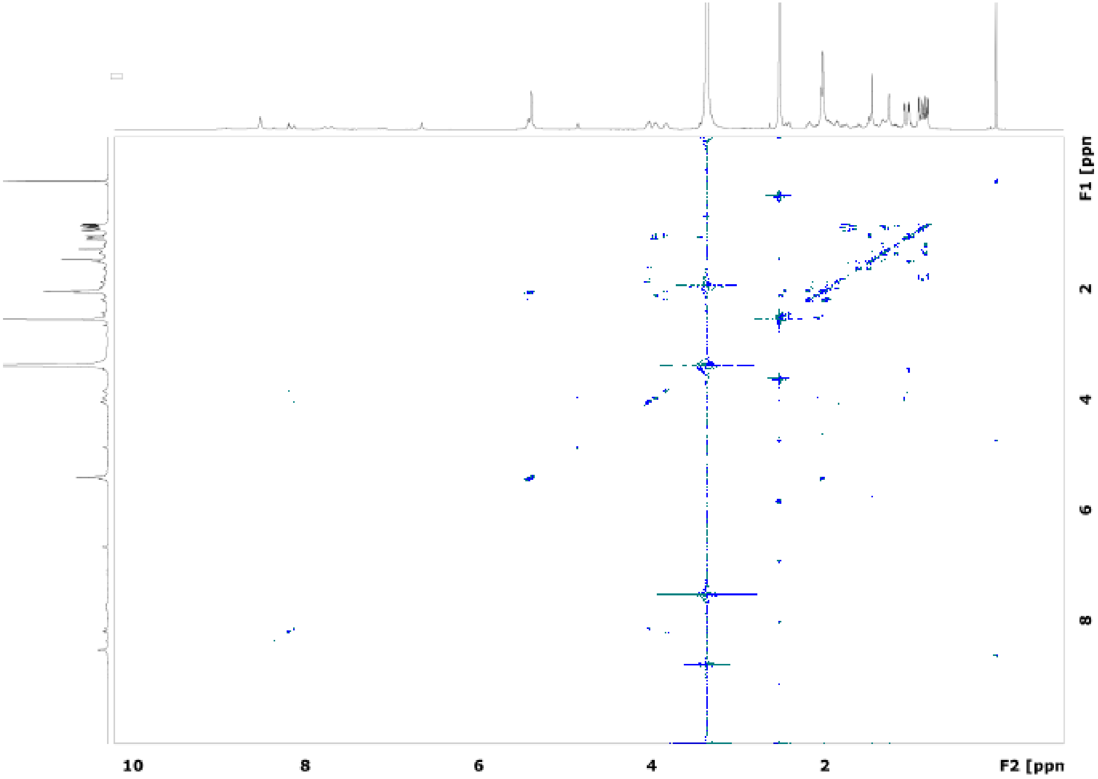


**Figure S14.** COSY spectrum of chitinimine I and III in DMSO-d6.

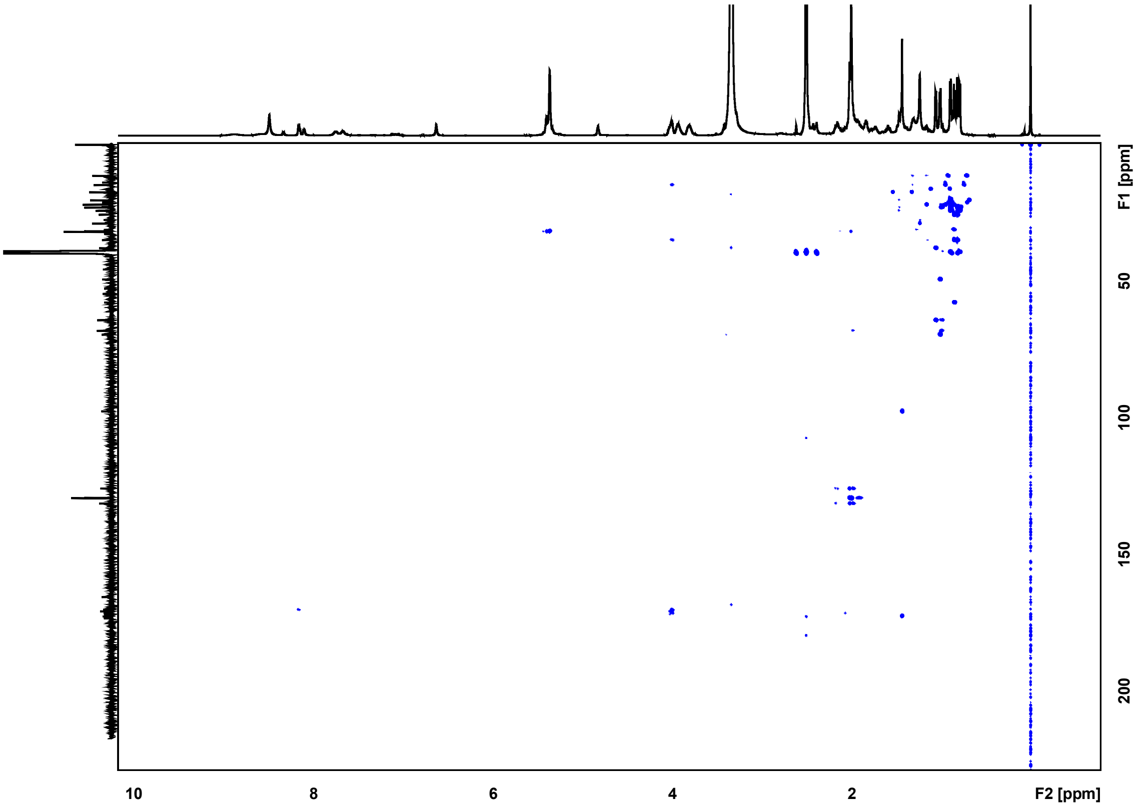


**Figure S15**. HMBC spectrum of chitinimine I and III in DMSO-d6.

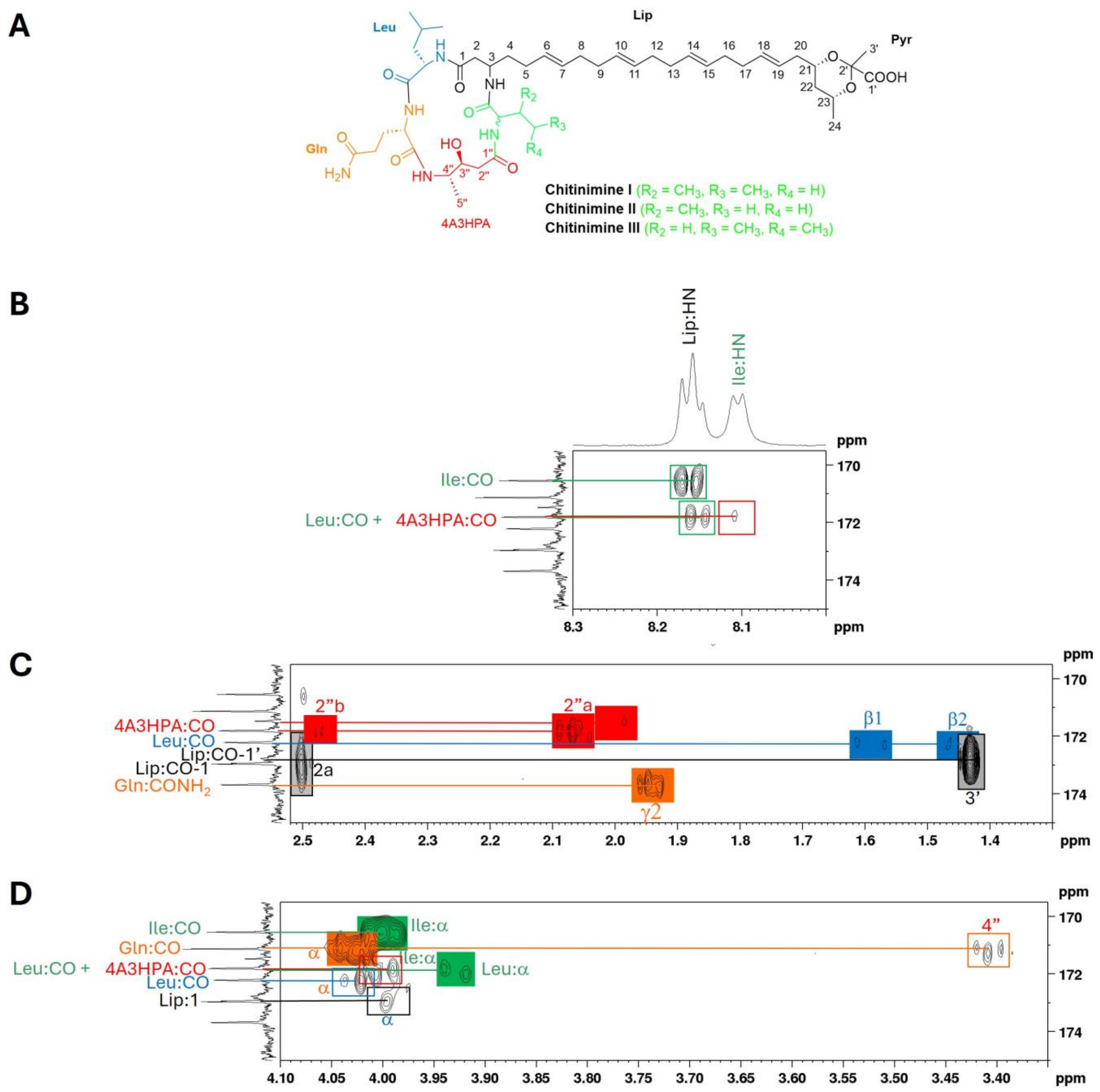


**Figure S16. Relevant sections of the HMBC spectrum for the assignment of carbonyl signals in chitinimine I and III.** (**A**) Labeling and color coding applied in following panels. (**B**) Unambiguous assignment for carbonyls of Ile and Leu-1 and in respectively chitinimine I and III was obtained through the interresidue correlation to Lip:HN. (**C**) Backbone carbonyl signals of Leu-2, 4A3HPA, the specialized lipid and the sidechain of Gln could be assigned from intraresidue HMBC cross peaks. (**D**) Correlation to 4A3HP: H-4" provided assignment of the backbone carbonyl in Gln.
Atom/position labelling as indicated in **Tables S4** and **S6**. Intra- and interresidue cross peaks are marked with solid and open boxes, respectively.

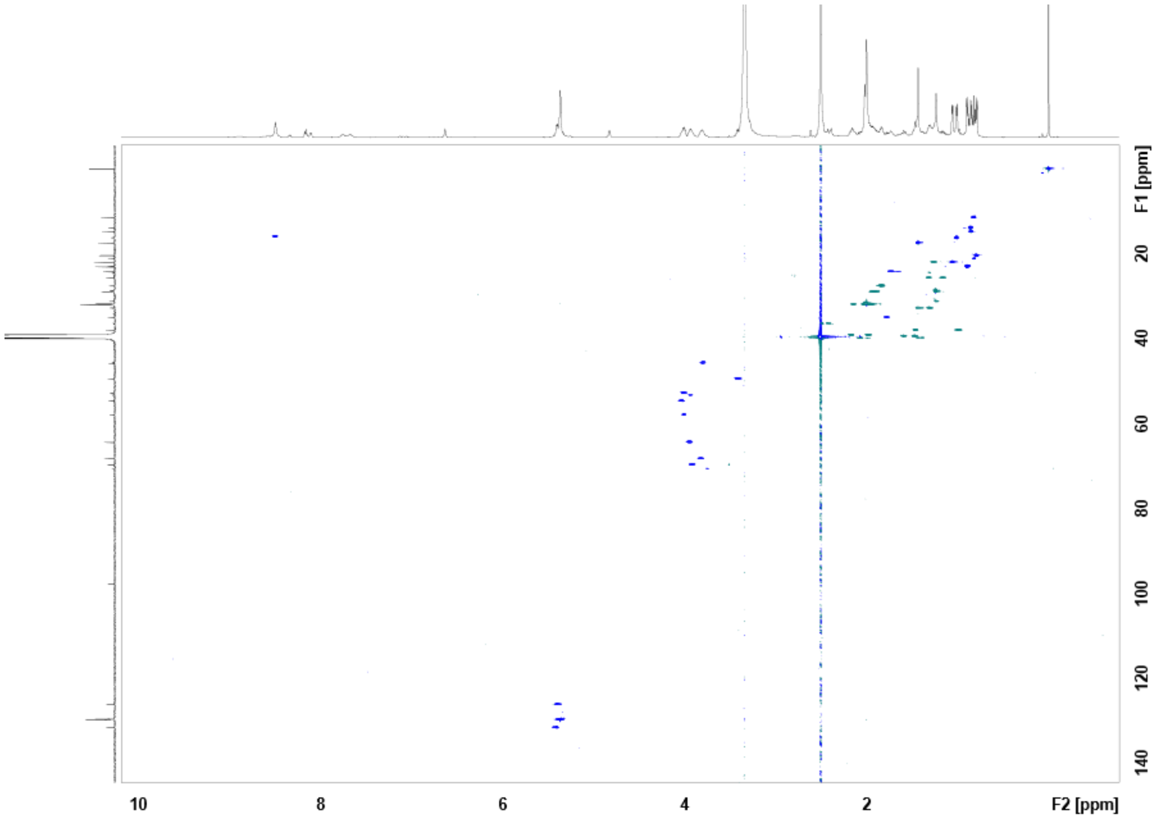


**Figure S17**. (Multiplicity-Edited) HSQC spectrum of chitinimine I and III in DMSO-d6.

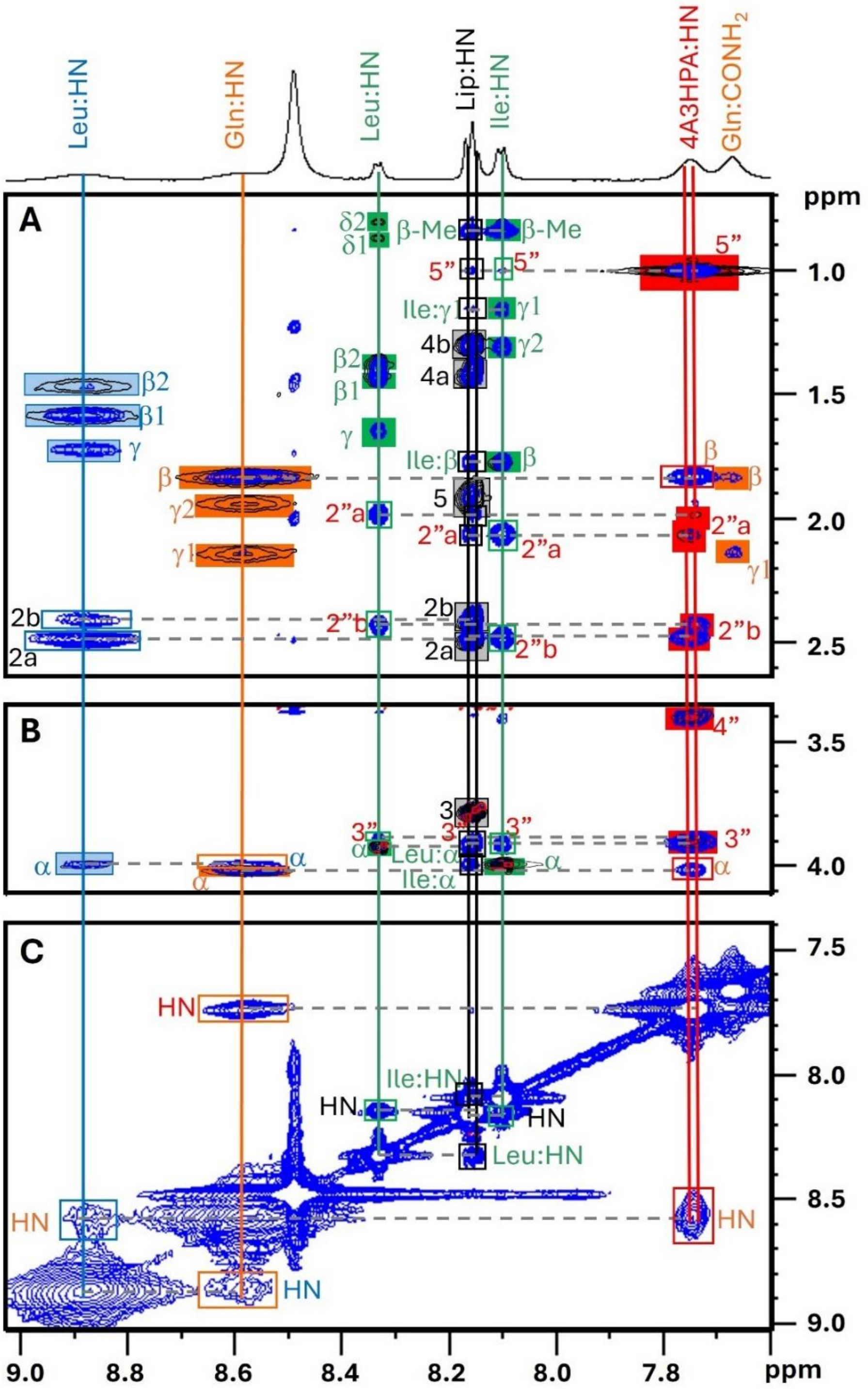


**Figure S18. NOE interactions for backbone amide proton signals in chitinimine I and III,** overlayed with corresponding regions in TOCSY (**A**, **B**) and COSY spectra (**B**). Labeling and color coding is according to the **Figure S16A**. Atom/position labelling as indicated in **Tables S4** and **S6**. Intraresidue and interresidue cross peaks are marked with solid and open boxes respectively.

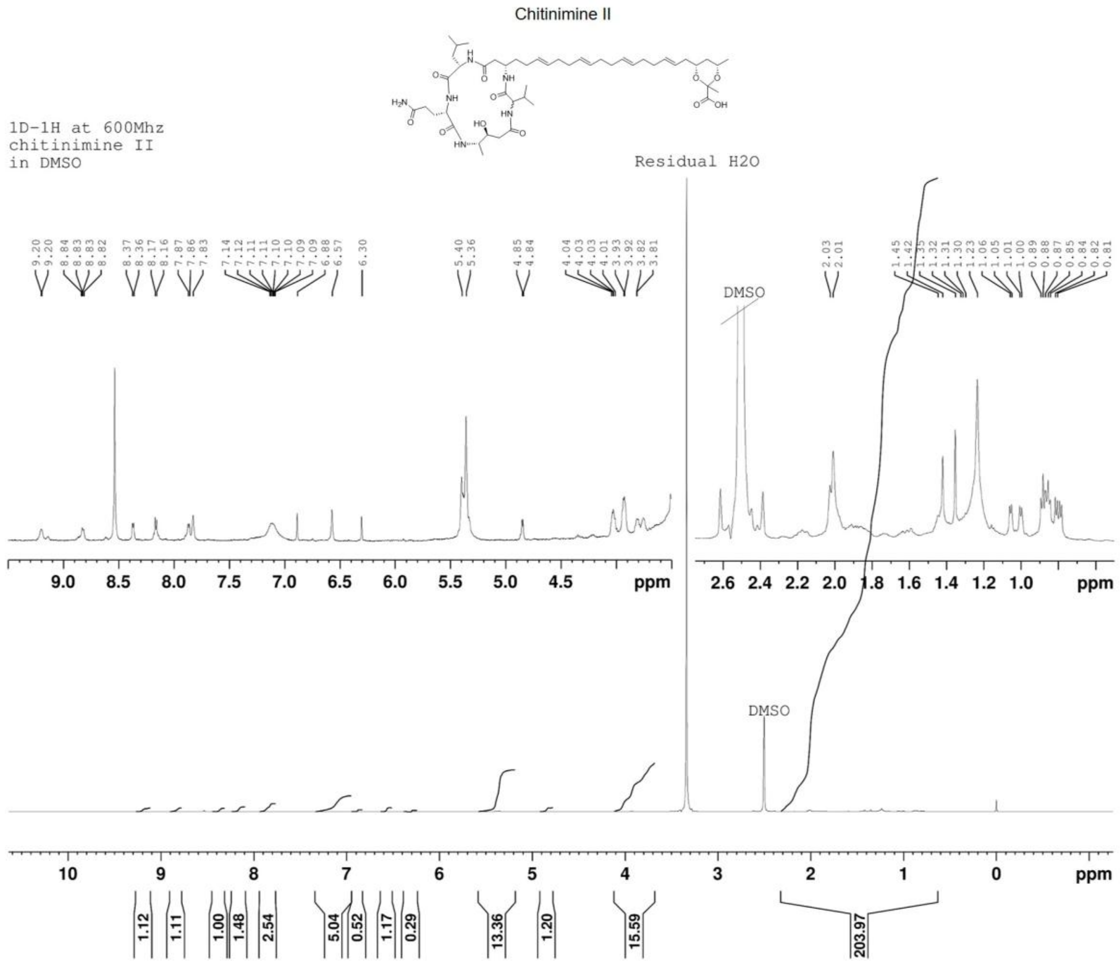


**Figure S19.** $^{1}H$ NMR spectrum of chitinimine II in DMSO-d6.

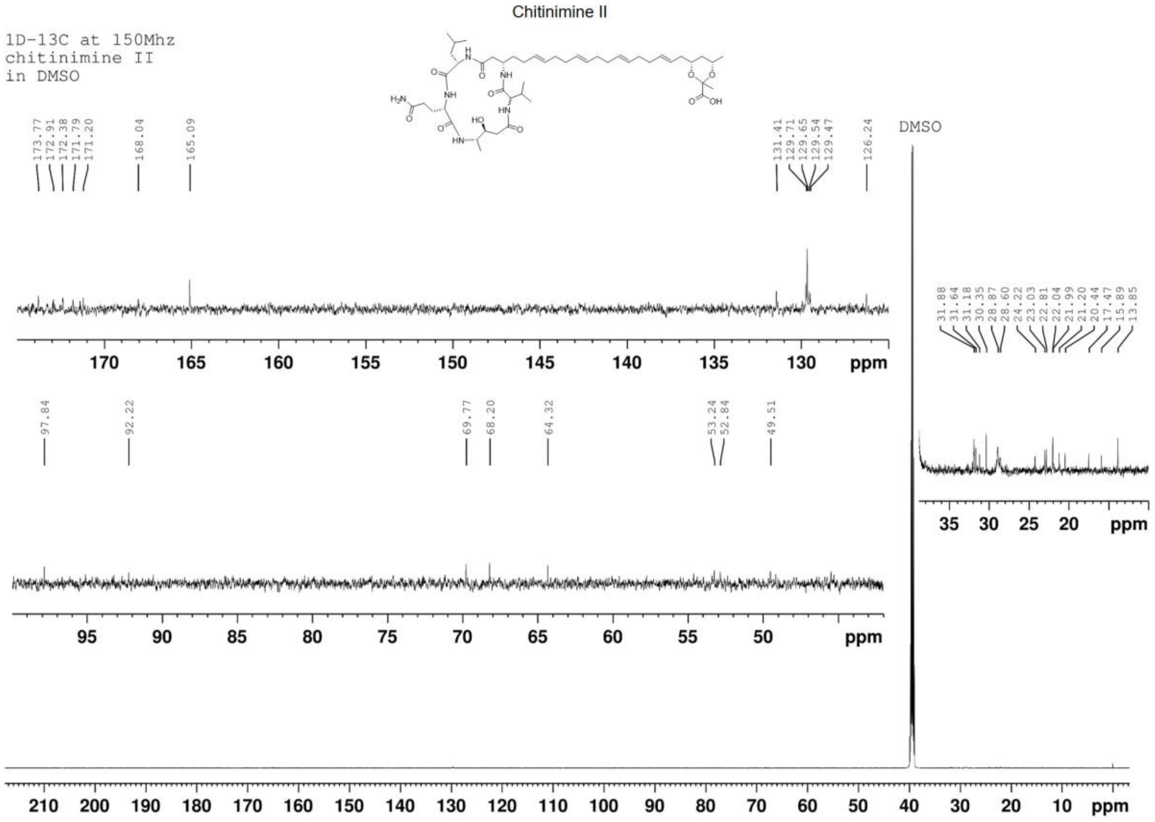


**Figure S20**. $^{13}$C NMR spectrum of chitinimine II in DMSO-d6.

A

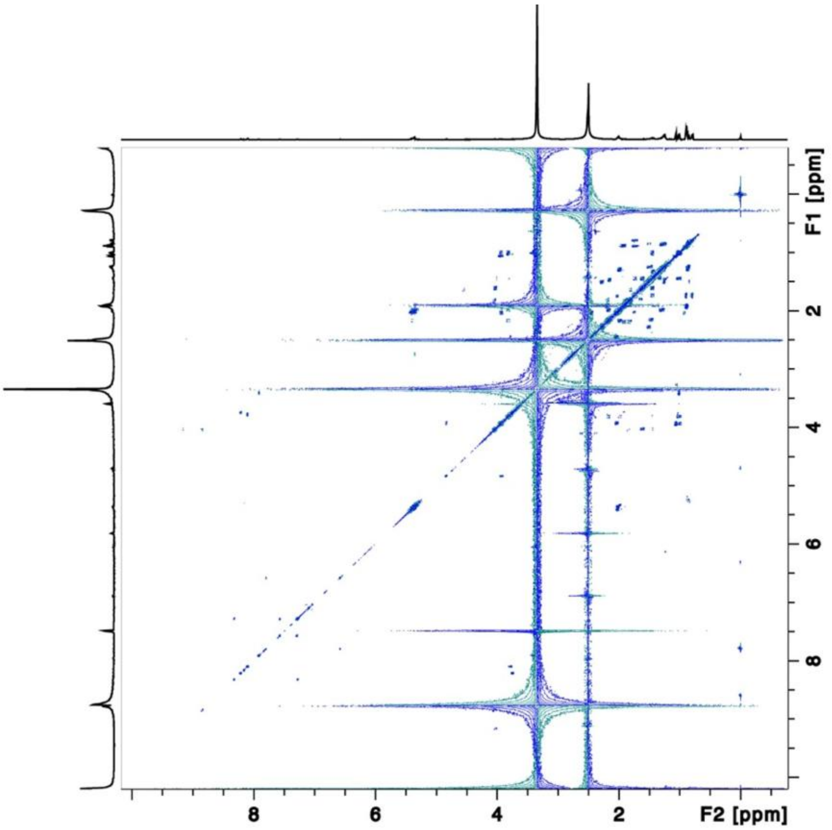


B

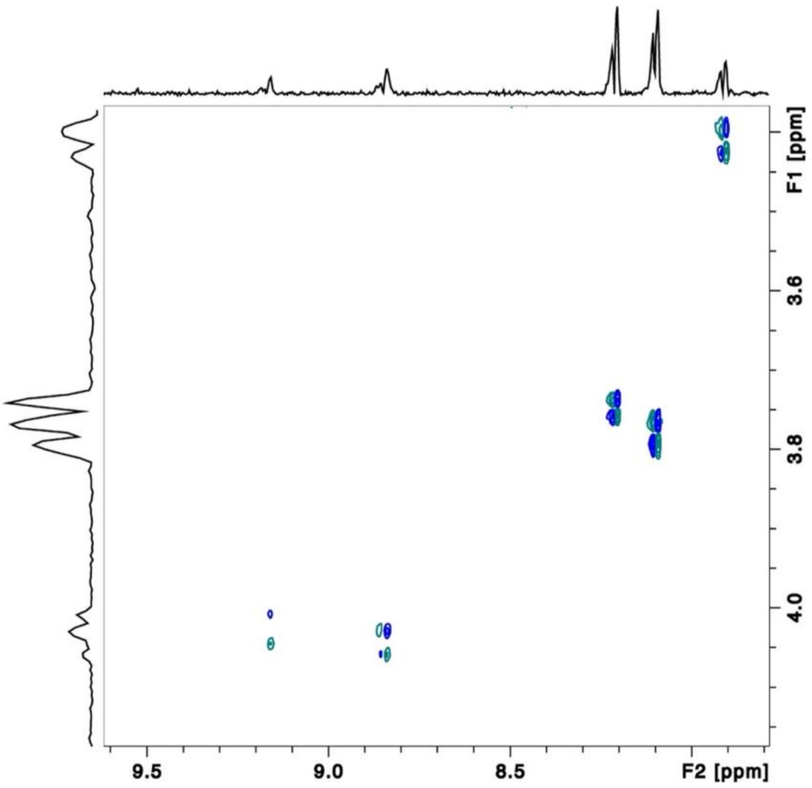


**Figure S21.** COSY spectrum of chitinimine II in DMSO-d6. (**A**) Full COSY spectrum. (**B**) Zoomed-in view of the COSY spectrum (F2: 7.8 – 9.6 ppm; F1: 3.4 – 4.15 ppm).

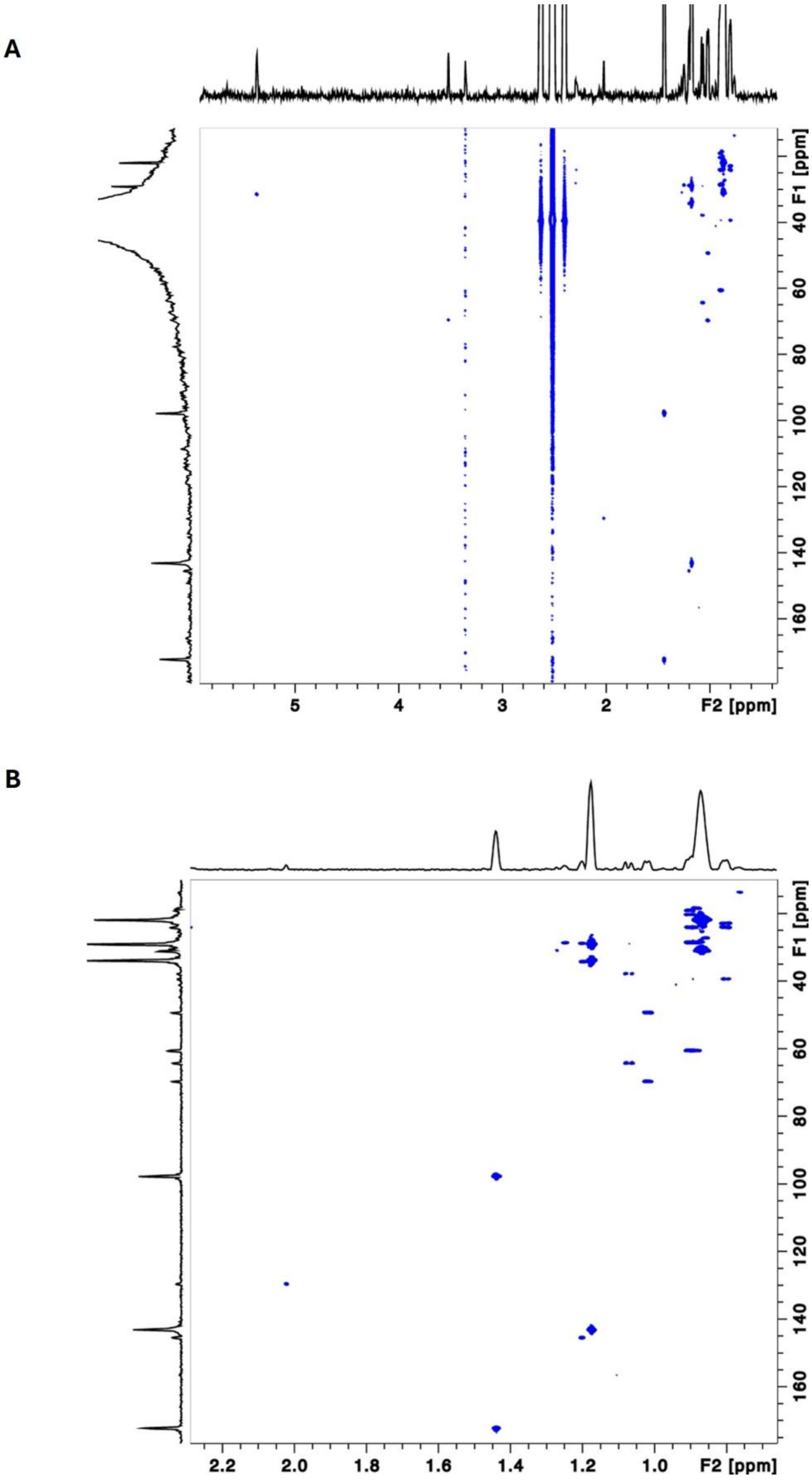


**Figure S22**. HMBC spectrum of chitinimine II in DMSO-d6. (**A**) Full HMBC spectrum. (**B**) Zoomed-in view of the HMBC spectrum (F2: 0.7 – 2.25 ppm; F1: 15 – 175 ppm).

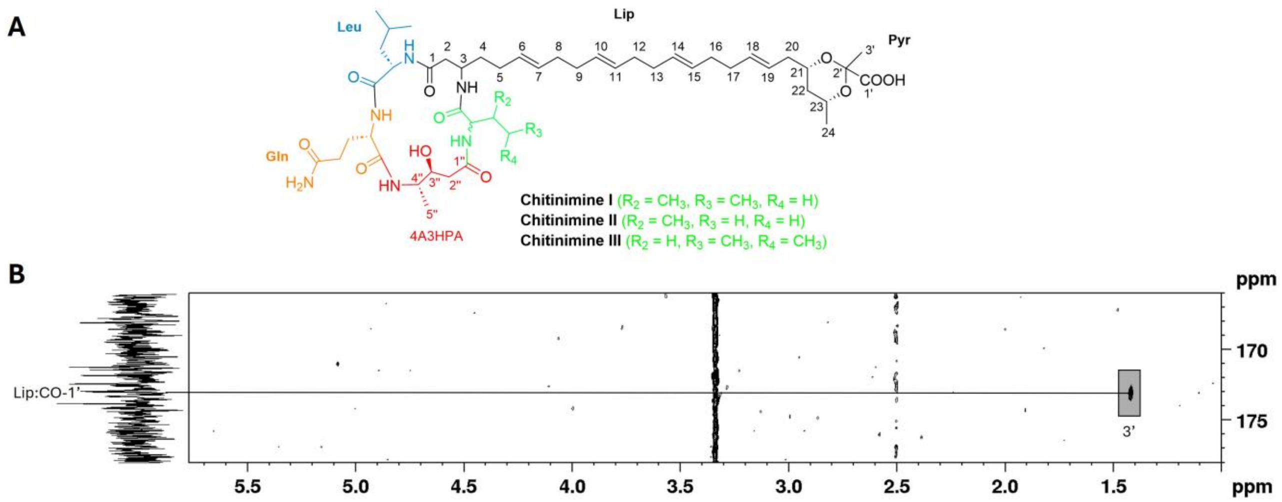


**Figure S23. Relevant section of the HMBC spectrum for the assignment of carbonyl signals in chitinimine II.** (**A**) Color and atom labelling used in chitinimine II. (**B**) Only Lip:CO-1 could be assigned through correlation with Lip:$CH_3$-3' due to low sample concentration.

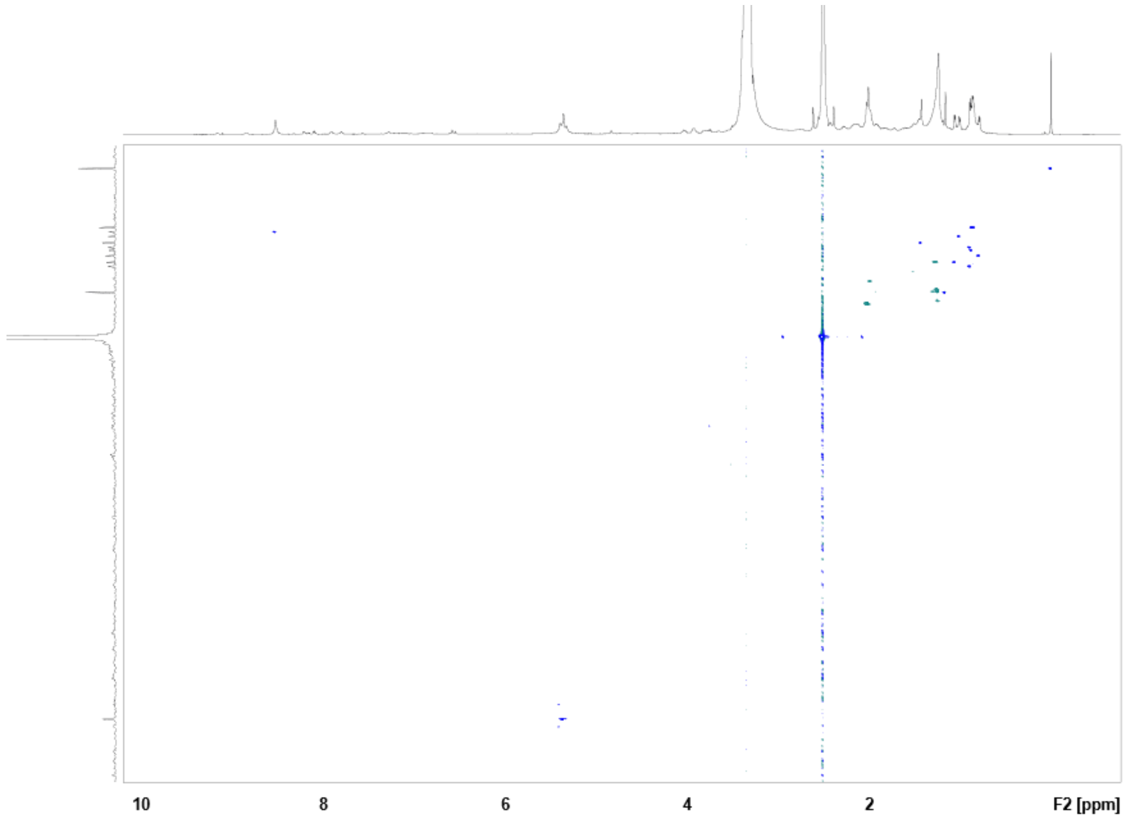


**Figure S24**. (Multiplicity-Edited) HSQC spectrum of chitinimine II in DMSO-d6.

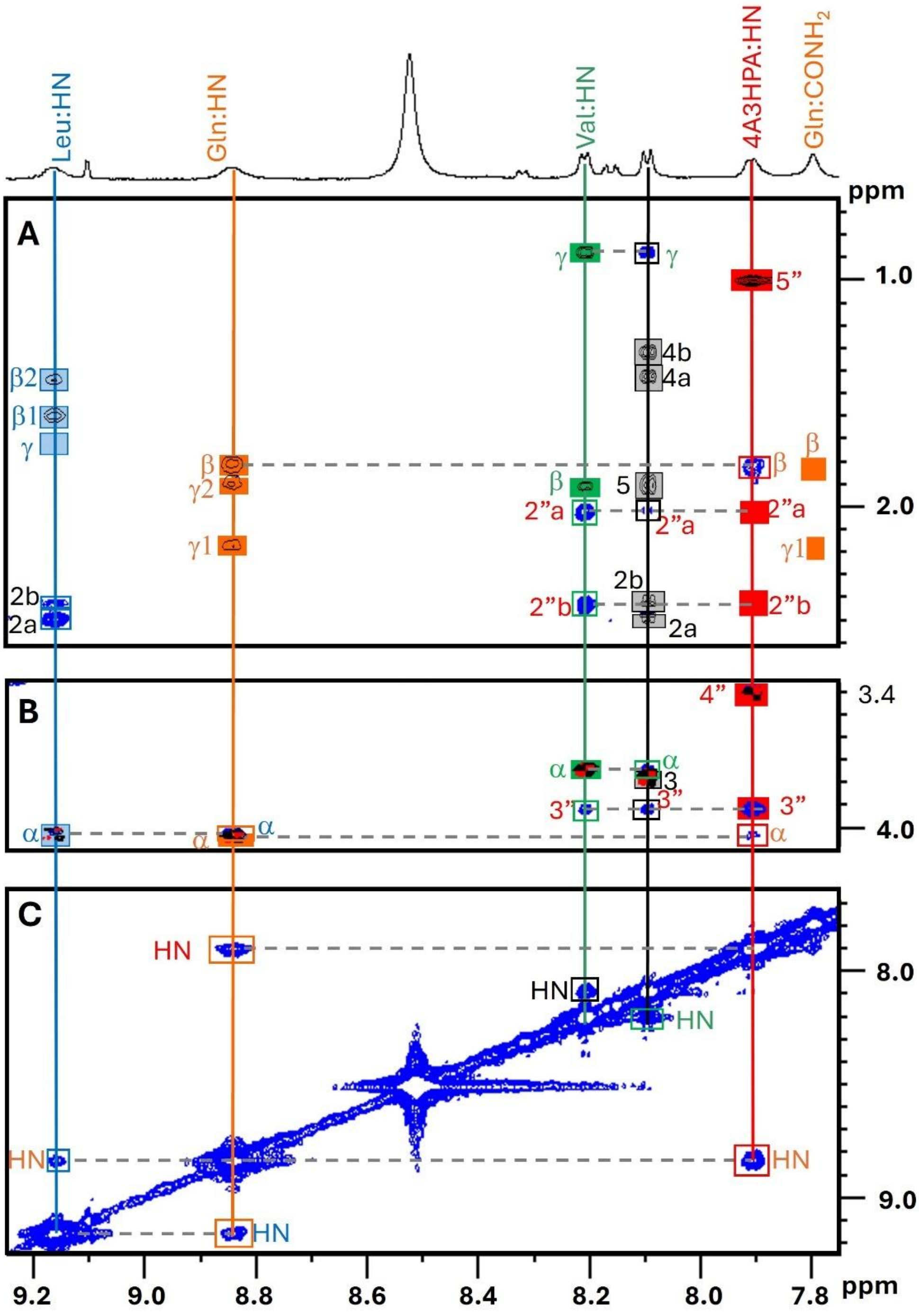


**Figure S25. Sections of the 2D NOESY spectrum with NOE interactions for backbone amide proton signals in chitinimine II,** overlayed with corresponding regions in TOCSY (**A**, **B**) and COSY spectra (**B**). Labeling and color coding is according to **Figure S23A** and **Table S5**. Intraresidue and interresidue cross peaks are marked with solid and open boxes, respectively.

**A**

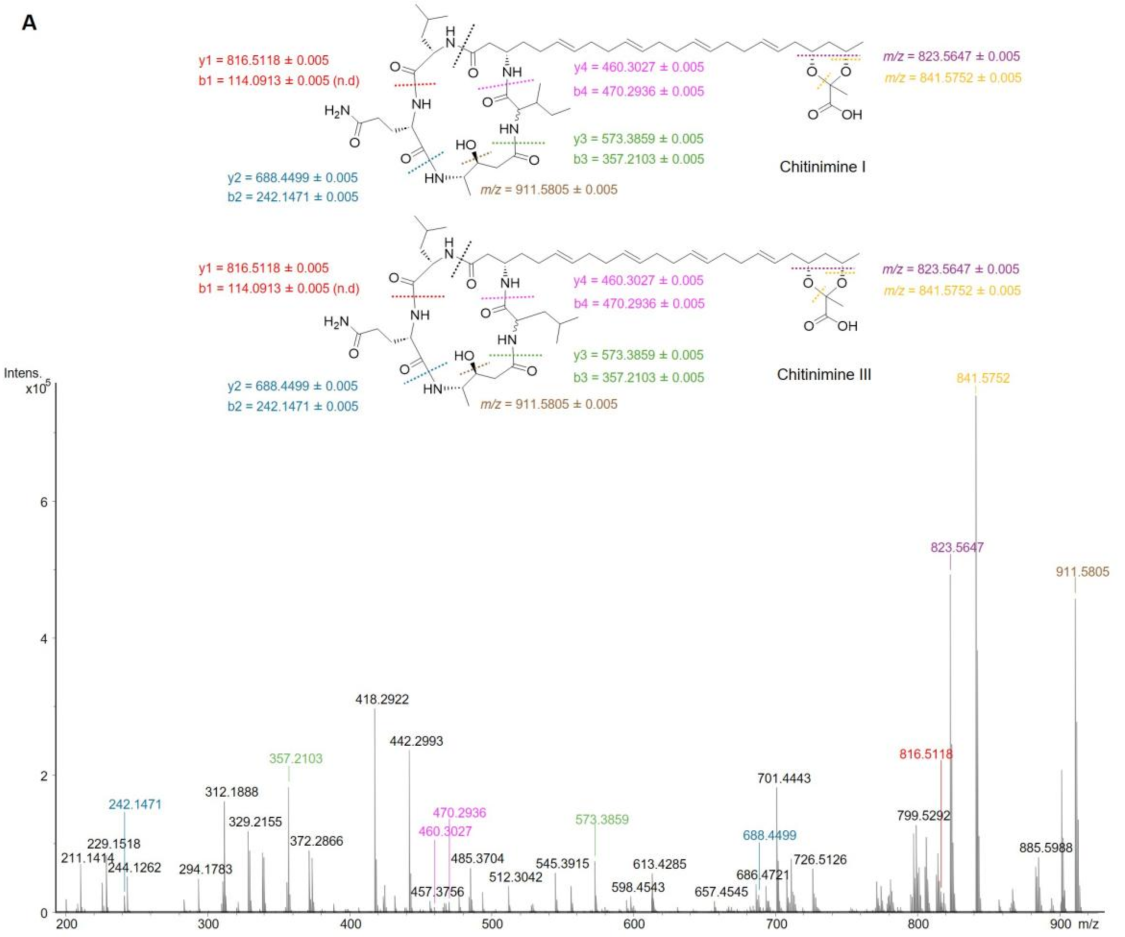


**B**

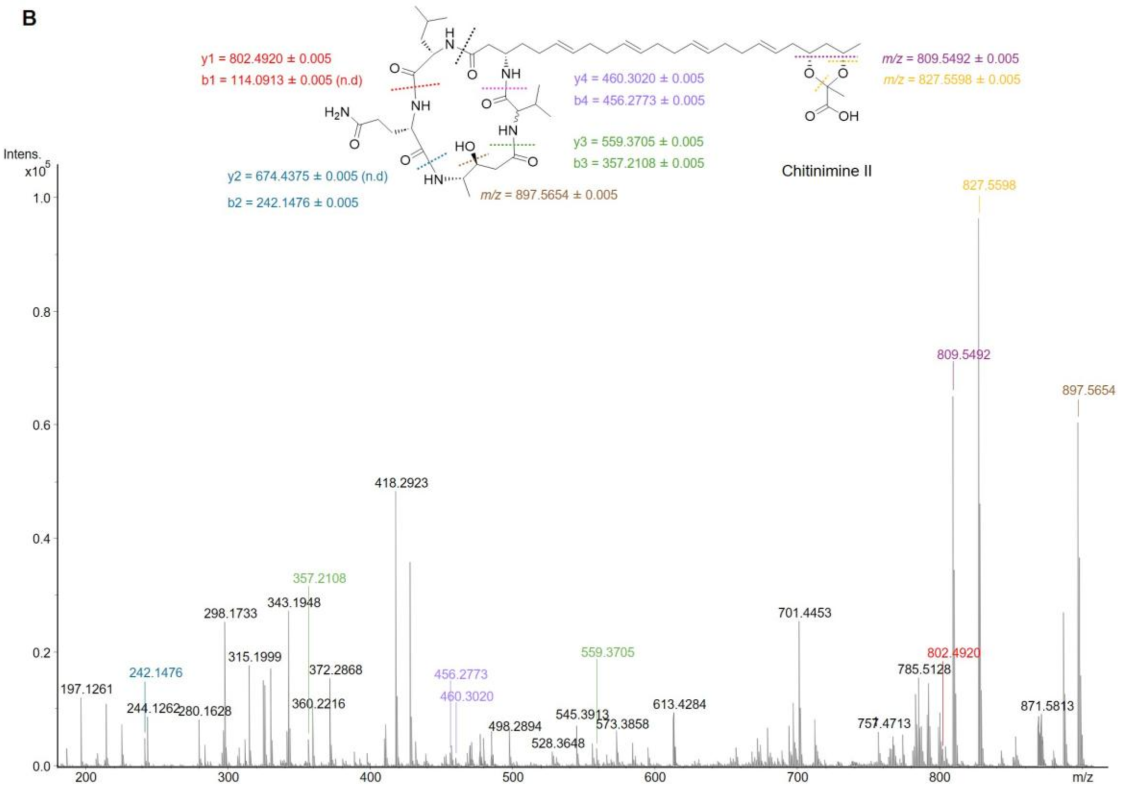


**Figure S26**. High-resolution LC-ESI-MS/MS spectra of (**A**) chitinimine I and III ($[M+H]^+$ = 929.5968 Da), and (**B**) chitinimine II ($[M+H]^+$ = 915.5815 Da). Selected b and y-fragment ions are indicated. Inset: structures of chitinimine I-III and detected fragment ions. nd = not determinable.

A

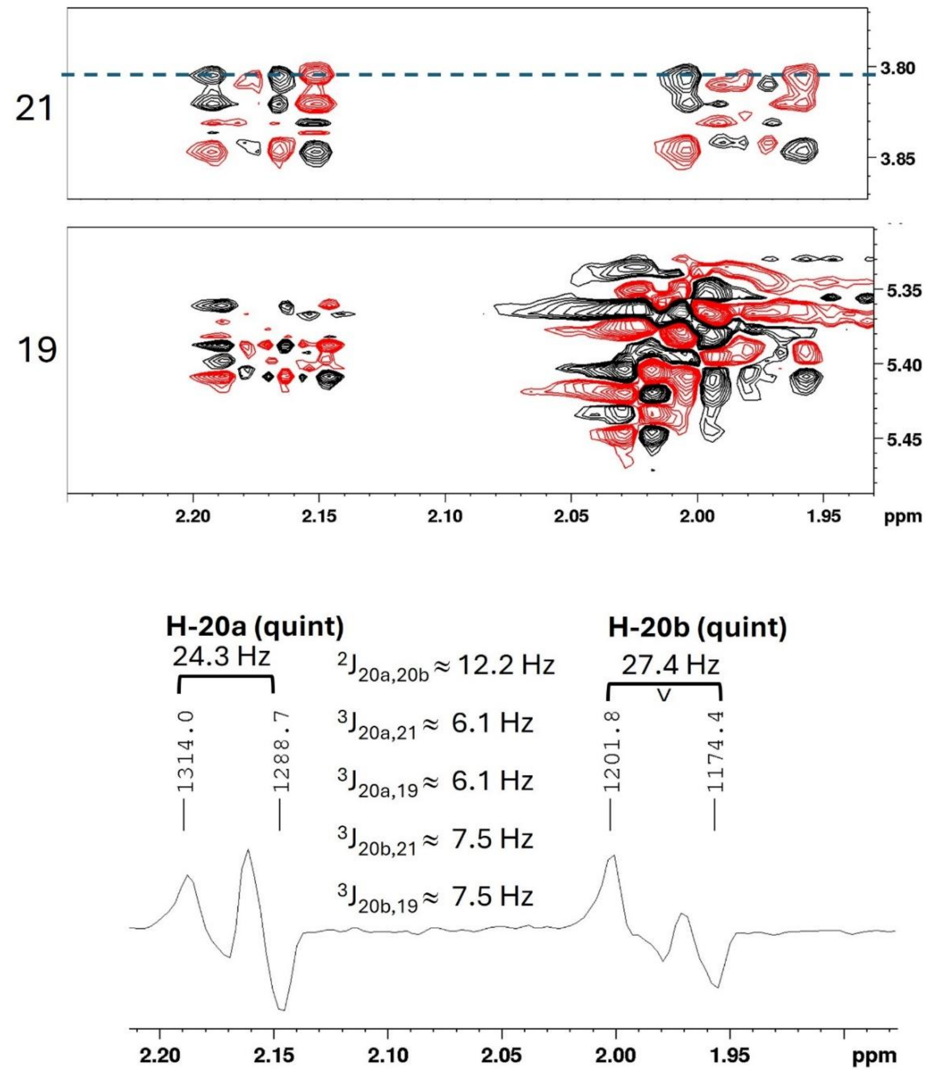
21
19
3.80
3.85
5.35
5.40
5.45
2.20
2.15
2.10
2.05
2.00
1.95
ppm
H-20a (quint)
24.3 Hz
H-20b (quint)
27.4 Hz
$^{2}J_{20a,20b} \approx 12.2$ Hz
$^{3}J_{20a,21} \approx 6.1$ Hz
$^{3}J_{20a,19} \approx 6.1$ Hz
$^{3}J_{20b,21} \approx 7.5$ Hz
$^{3}J_{20b,19} \approx 7.5$ Hz
1314.0
1288.7
1201.8
1174.4

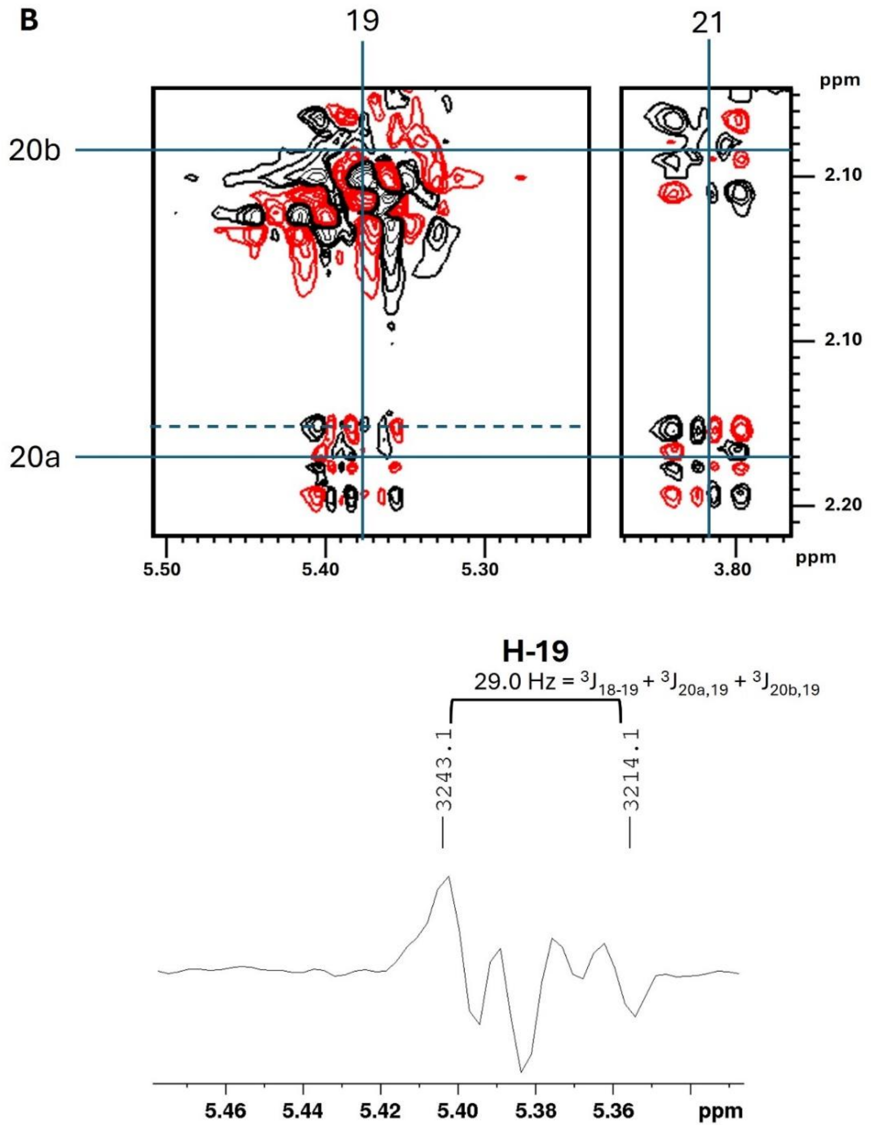


**Figure S27. Focus on the crosspeaks of H-20 protons to H-19 and H-20 in the DQFCOSY of chitinimine I and III.** (**A**) A dashed line indicates the position of the trace at the bottom that was used to determine the total width of H-20a and H-20b. (**B**) A dashed line indicates the position of the trace at the bottom that was used to determine the total width of H-19. The size of $^3J_{18\text{-}19}$ was estimated by subtracting $^3J_{20a,19}$ + $^3J_{20b,19}$ from the total width of H-19 (29.0 – 7.5 – 6.1 = 15.4 Hz).

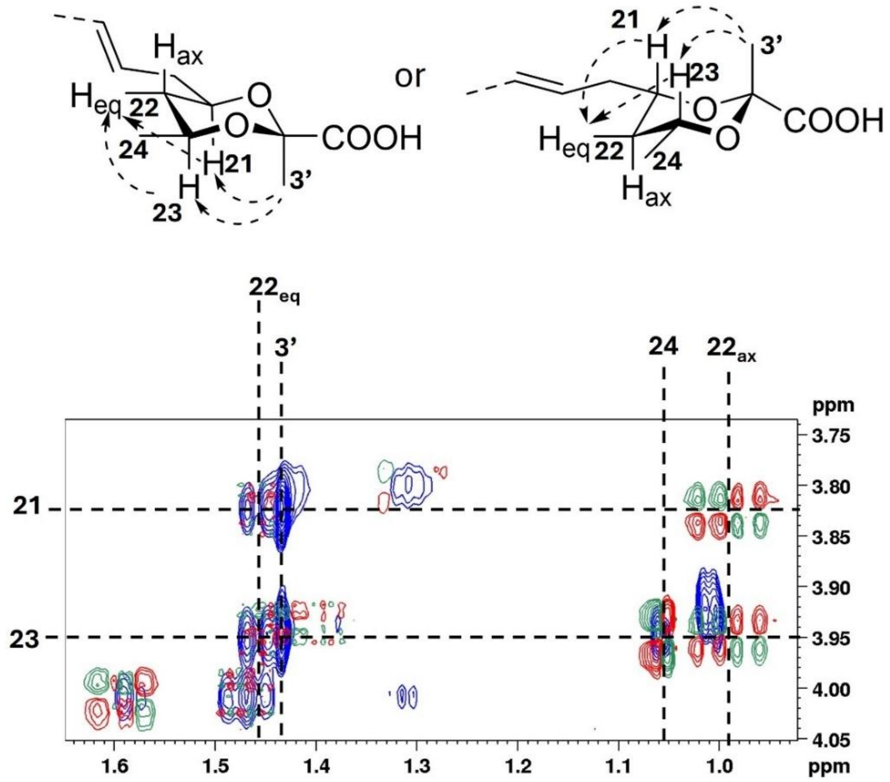


**Figure S28. Focus on the crosspeaks of H-22 protons to H-21 and H-23 in the DQFCOSY of chitinimine I and III, overlayed on the corresponding region in the NOESY spectrum.** Nearly identical COSY patterns and NOE interactions for crosspeaks of H23 and H21 to protons at C22 are observed in the six membered ring. NOE interaction of 3’-$CH_3$ in the pyruvate moiety to H-21 and H-23 of the specialized lipid is in agreement with the chair conformation of the ring having H-21, H-23 and 3’-$CH_3$ in an axial orientation. The axial orientation of H-21 and H-23 is confirmed with their strong $^3J$ to H-$22_{ax}$ and weak $^3J$ to H-$22_{eq}$.

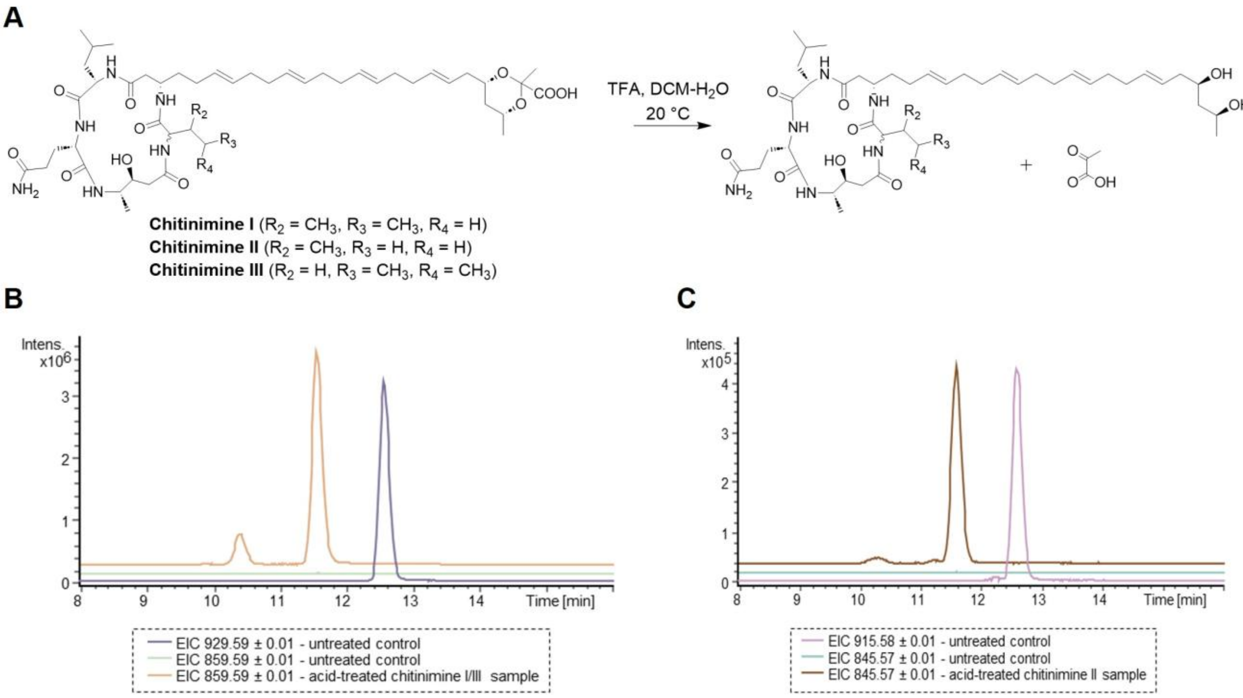


**Figure S29. Acid hydrolysis of the chitinimines.** (**A**) Samples containing either chitinimine I/III or chitinimine II were hydrolyzed using TFA, releasing pyruvic acid. (**B**) Extracted ion chromatograms at *m/z* = 929.5962 ± 0.01 (corresponding to the $[M+H]^+$ ion for chitinimine I/III) and *m/z* = 859.5902 ± 0.01 (corresponding to the $[M+H]^+$ ion for hydrolyzed chitinimine I/III) from UHPLC-ESI-Q-TOF-MS analyses of TFA-treated chitinimine I/III. (**C**) Extracted ion chromatograms at *m/z* = 915.5879 ± 0.01 (corresponding to the $[M+H]^+$ ion for chitinimine II) and *m/z* = 845.5746 ± 0.01 (corresponding to the $[M+H]^+$ ion for hydrolyzed chitinimine II) from UHPLC-ESI-Q-TOF-MS analyses of TFA-treated chitinimine II.

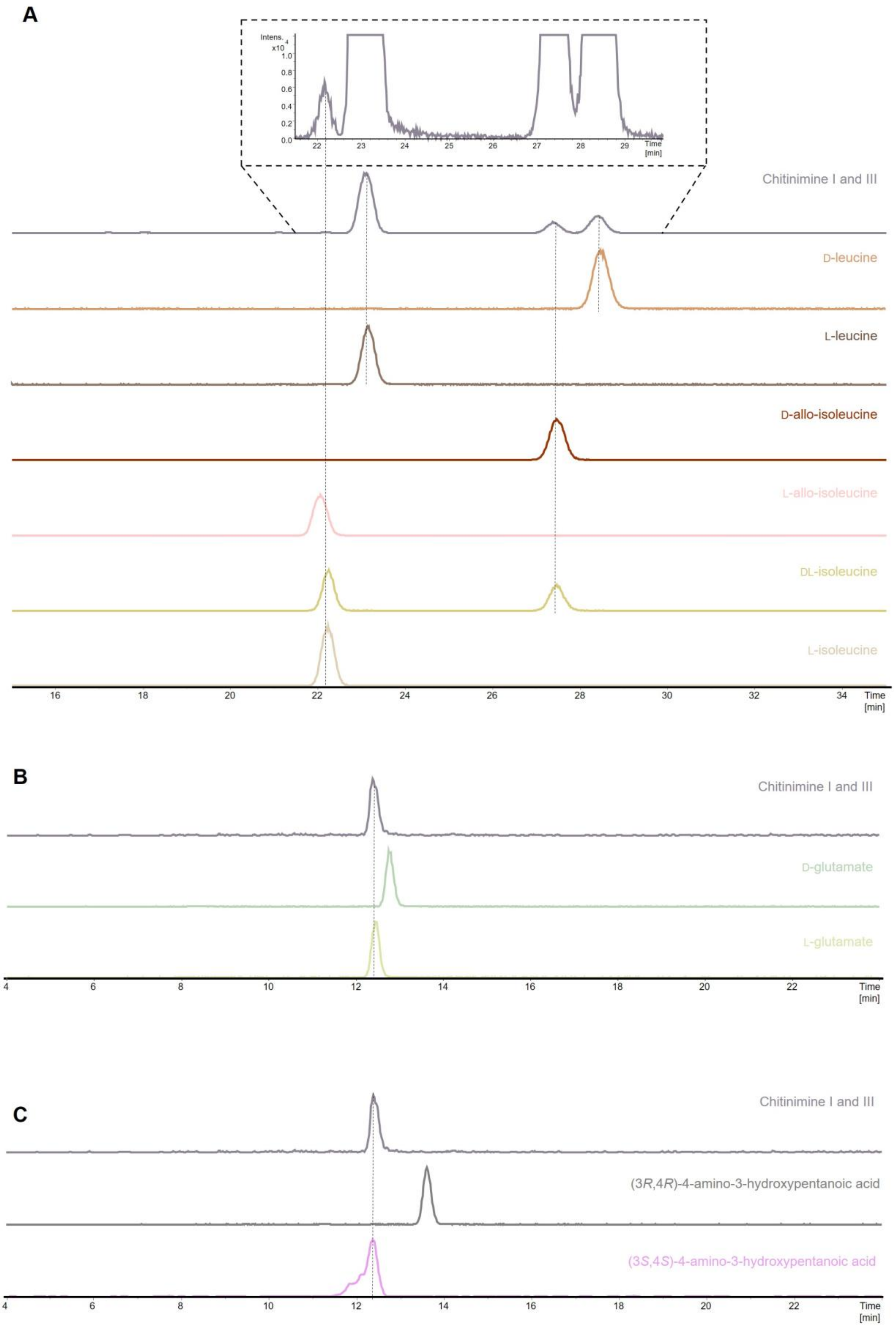
A
Intens. x10^4
Time [min]
Chitinimine I and III
D-leucine
L-leucine
D-allo-isoleucine
L-allo-isoleucine
DL-isoleucine
L-isoleucine
Time [min]
B
Chitinimine I and III
D-glutamate
L-glutamate
Time [min]
C
Chitinimine I and III
(3R,4R)-4-amino-3-hydroxypentanoic acid
(3S,4S)-4-amino-3-hydroxypentanoic acid
Time [min]

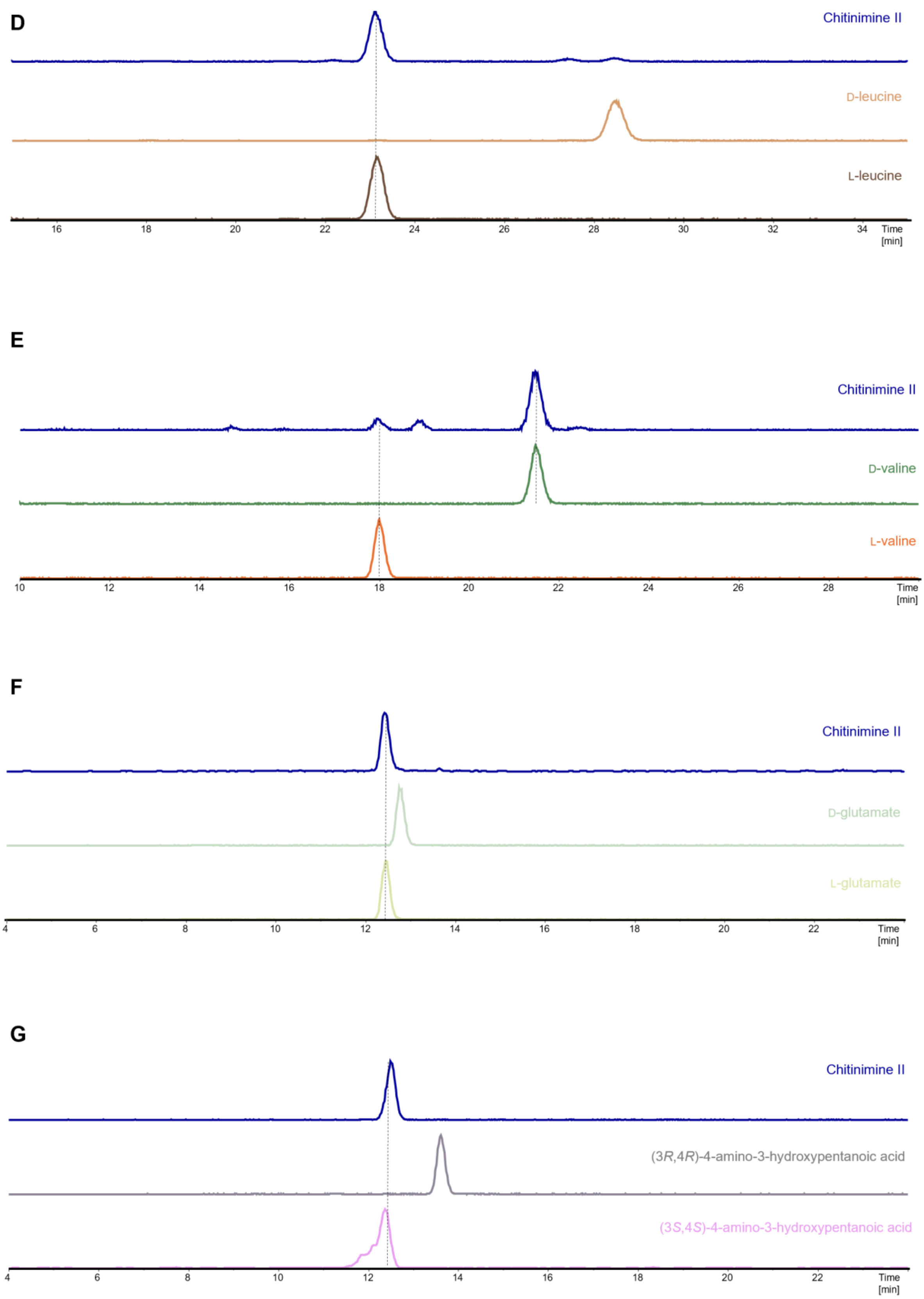


**Figure S30. Determination of the absolute stereochemistry of the chitinimine amino acids using Marfey's method.** (**A**) Extracted ion chromatograms at *m/z* = 384.1514 ± 0.005 (corresponding to $[M+H]^+$ for the Marfey's derivatives of Leu, Ile and *allo*-Ile) from LC-MS analyses comparing the derivatives from chitinimine I and III hydrolysates with the derivatives of the authentic standards D/L-Leu, D/L-Ile and D/L-*allo*-Ile. (**B**) Extracted ion chromatograms at *m/z* = 400.1099 ± 0.005 (corresponding to $[M+H]^+$ for the Marfey's derivative of Glu) from LC-MS analyses of Marfey's derivative of D-Glu and L-Glu and the derivative from the acid-hydrolysed chitinimine I and III. (**C**) Extracted ion chromatograms at *m/z* = 386.1306 ± 0.005 (corresponding to $[M+H]^+$ for the Marfey's derivative of 4-amino-3-hydroxypentanoic acid) from LC-MS analyses of Marfey's derivative of authentic standards (3*S*,4*S*)-4-amino-3-hydroxypentanoic acid and (3*R*,4*R*)-4-amino-3-hydroxypentanoic acid, and the derivative from the acid-hydrolysed chitinimine I and III. (**D**) Extracted ion chromatograms at *m/z* = 384.1514 ± 0.005 (corresponding to $[M+H]^+$ for the Marfey's derivatives of Leu) from LC-MS analyses comparing the derivatives from chitinimine II hydrolysates with the derivatives of the authentic standards D/L-Leu. (**E**) Extracted ion chromatograms at *m/z* = 370.1357 ± 0.005 (corresponding to the $[M+H]^+$

for the Marfey's derivative of Val) from LC-MS analyses of Marfey's derivative of authentic standards of D-Val and L-Val. (**F**) Extracted ion chromatograms at *m/z* = 400.1099 ± 0.005 (corresponding to $[M+H]^+$ for the Marfey's derivative of Glu) from LC-MS analyses of Marfey's derivative of D-Glu and L-Glu and the derivative from the acid-hydrolysed chitinimine II. (**G**) Extracted ion chromatograms at m/z = 386.1306 ± 0.005 (corresponding to the $[M+H]^+$ for the Marfey's derivative of 4-amino-3-hydroxypentanoic acid) from LC-MS analyses of Marfey's derivative of authentic standards (3*S*,4*S*)-4-amino-3-hydroxypentanoic acid and (3*R*,4*R*)-4-amino-3-hydroxypentanoic acid, and the derivative from the acid-hydrolysed chitinimine II.

**A**

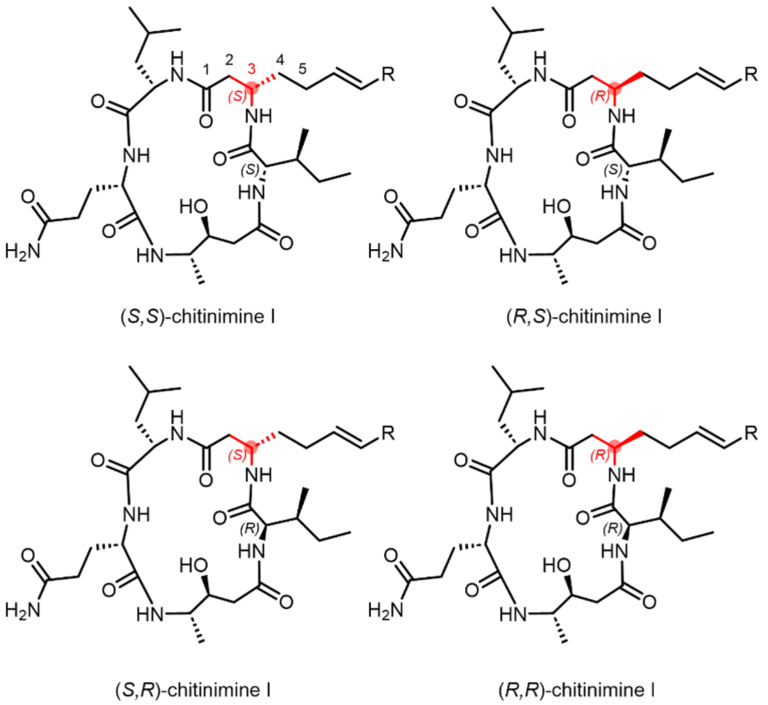


**B**

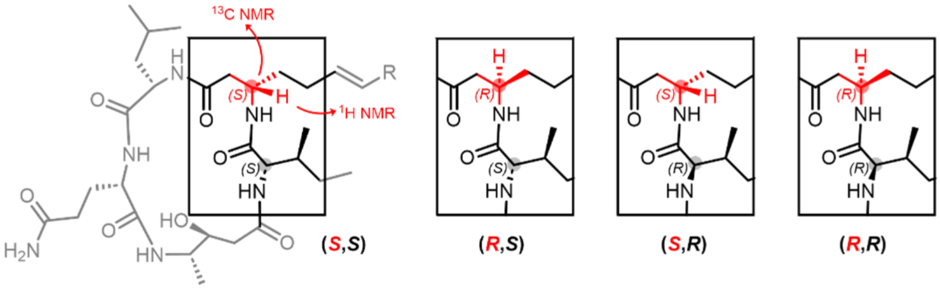


**Figure S31. Stereochemical prediction of C3 in chitinimines via DFT calculations.** (**A**) Representation of all possible stereoisomers of chitinimine I considered in the DFT modelling. (**B**) The stereochemistry at C3 of the specialized lipid is tentatively assigned as *S*. For the sake of simplicity, the lipid chain was shortened to R=H.

```
ChtnE_AT4      LAAGVGPLLPGLVDALCANHRDAEADTRALYPRQLAPLAAEQ-----------------R  43
ChtnD_AT3      LFAGQGAQTVGMGARLCIDHPAARACFEA----FDAAGRAQGSAPISALVFPPDTFDAGR  56
ChtnA_AT1      --PGQGNPQPGAAAALYRDHAGFRAAIDR----CAALLDELLDVPLATLLFD-----A--  47
ChtnB_A2       -FPGQGSQRAGMGHAIYAAEPAFRALADA----CLALLPAELAAQVRLAAFADPAEAG--  53
                  * *    *    :    .    .*           *

ChtnE_AT4      AAIEEALLADNQALIKAGVIASWQYTRLLTERIGLAPAARFGHSLGQASMLFAAGAWLPD  103
ChtnD_AT3      AEAQRAALTQTQHAQAAIGAYNMALYGVL-RSAGFAPDMALGHSFGELSALWAAGALDDA  115
ChtnA_AT1      -ERGGPLLRQTRYAQPAQFALGWALAQLW-AGWGVRPAALAGHSLGEYVAAVLAGMAPLE  105
ChtnB_A2       -AEAEALLARTEVAQPALFVHQYALARLL-LDWGLRPAGLLGHSLGEIVAAALAGVLTPP  111
                       *  ..     *          :     *. *    ***:*:       **

ChtnE_AT4      DGWMQRLAALG----DSLGRLSGELPAVRAAWNLAEGEPLDWANLLVLAPAERVLAAARS  159
ChtnD_AT3      -GYRAAVLARGSALTPPAGREVGGLIAV-------------------SAPAEQVSALLPQ  155
ChtnA_AT1      -QVLPLVARRGALMQETAD--GAAMLLV-------------------RAGAADVAALVEA  143
ChtnB_A2       -DAIQLVVRRARLMQDSAE--GAMLQAE--------------------LPA---AELAAL  145
                     :   .           . :                          *

ChtnE_AT4      E-PRAYVSLVNSPAEATLVGDRAACGRILAAIGA---EAVPAPDSLVMHCAPAAGERAAI  215
ChtnD_AT3      L-PGLALANLNSPRQTVAGGSAAAVEAALPRLAEAGLQAVRLPVAAAFHTGLV-DYAAAP  213
ChtnA_AT1      RPAELALAADNGPASCVLAGTAAAIEAAAAGLAGRGIRSRLLDVAAAFHSPLM-EPIVLR  202
ChtnB_A2       LPDGLAIAACNGPALTVAAGPVAQVEAFAAQLDARGVAWQRLRGRHAFHSPAM-APAAAA  204
                     ::  *.*   .  *  *         :              .:*         .

ChtnE_AT4      AARFTAPLGPRPEGLEFAGGVPAEWSPA---ALAERVAADLVAPLDFPALVERVYGRGVR  272
ChtnD_AT3      WQAALAGLPLAAPRLPVWANVSAQPYPADAAGIRALLARQPFEPVRFCEQVEAAYAAGGR  273
ChtnA_AT1      LAGLAAMVDWQAGGVPAIANLHGRRH--AGAPDAAYWAAHARGTVRYREGLEALLADGHR  260
ChtnB_A2       LRPWLATLTAHPPQWPLLSNLDGGWMSDAEAVDPQRWARQLCAPVQFAPALAELAGRPGA  264
                    * :          ..: .              * .    : :    :    .

ChtnE_AT4      LFLEIGPGANCSRWIA--------         288
ChtnD_AT3      IFVEIGPRGILSRLVGDILG----         293
ChtnA_AT1      LFLELGPRPALATLGPTLAGGAEA         284
ChtnB_A2       LLLEIGAGTTLAAFARQA------         282
               :::*:*     :

ChtnE_AT4      ------------------------------------------------------------ 0
ChtnD_AT3      LFAGQGAQTVGMGARLCIDHPAARACFEAFDAAGRAQGSAPISALVFPPDTFDAGRAEAQ  60
ChtnA_AT1      --PGQGNPQPGAAAALYRDHAGFRAAIDRCAALLDELLDVPLATLLFD-----A---ERG  50
ChtnB_AT2      -FPGQGSQRAGMGHAIYAAEPAFRALADACLALLPAELAAQVRLAAFADPAEAG---AEA  56
```

**Figure S32. Sequence alignment of acyltransferase (AT) domains from the chitinimines BGC.** The conserved catalytic triad GxSxG is highlighted in yellow, substrate specificity determinants in blue.[52–55]

```
TropR-I_jweed       MEESKVSMMNCNNEGRWSLKGTTALVTGGSKGIGYAIVEELAG-LG-ARVYTCSRN-EK-       56
TropR-II_jweed      ------------MAGRWNLEGCTALVTGGSRGIGYGIVEELAS-LG-ASVYTCSRN-QK-       44
GlcDh_bacme         --------------MYKDLEGKVVVITGSSTGLGKSMAIRFAT-EK-AKVVVNYRSKED-       43
KR-fas_brana        --------------------SPVVVVTGASRGIGKAIALSLGK-AG-CKVLVNYARSAK-       37
KR-fas_ecoli        ----------------MNFEGKIALVTGASRGIGRAIAETLAA-RG-AKVIGTATS-EN-       40
KR1_ave             ---------------------GTTLITGGTGALATHLTHHLTTHQPTQHLLLTSRTGPHT       39
KR1_ery             ---------------------GTVLVTGGTGGVGGQIARWLAR-RGAPHLLLVSRSGPDA       38
KR2_ery             ---------------------GTILVTGGTAGLGAEVARWLAG-RGAEHLALVSRRGPDT       38
ChtnD_KR            ----------------------LVLVTGGARGVTARCIEALAA-RVPARFVLIGRSAPMA       37
ChtnB_KR            ----------------------VTLITGGFGRVGQAFARRLAA-RPGARLVLLGRQVPAG       37
                                          ::**.    :        :         .

TropR-I_jweed       ---------------------------------------------ELDECLEIWREKGL       70
TropR-II_jweed      ---------------------------------------------ELNDCLTQWRSKGF       58
GlcDh_bacme         ---------------------------------------------EANSVLEEIKKVGG       57
KR-fas_brana        ---------------------------------------------AAEEVSKQIEAYGG       51
KR-fas_ecoli        ---------------------------------------------GAQAISDYLGA---       51
KR1_ave             PHAQHLT--T------------------------------------------QLQQKGI       54
KR1_ery             DGAGELV--A------------------------------------------ELEALGA       53
KR2_ery             EGVGDLT--A------------------------------------------ELTRLGA       53
```

```
ChtnD_KR          ADPAWAAGIAEPAALRARALQQLREEGTAPTPRLVEARCQAVLAGREVRQTLQTLAAHGA    97
ChtnB_KR          DDPRLLELRA-----------------------------------------------LGA    50


TropR-I_jweed     NVEGSVCDLLSRTERDKLMQTVAHVFDGKLNILVNNAGVVI---HKEAKDFTEKDYNIIM    127
TropR-II_jweed    KVEASVCDLSSRSERQELMNTVANHFHGKLNILVNNAGIVI---YKEAKDYTVEDYSLIM    115
GlcDh_bacme       EAIAVKGDVTVESDVINLVQSAIKE-FGKLDVMINNAGLEN---PVSSHEMSLSDWNKVI    113
KR-fas_brana      QAITFGGDVSKEADVEAMMKTAIDA-WGTIDVVVNNAGITR---DTLLIRMKKSQWDEVI    107
KR-fas_ecoli      NGKGLMLNVTDPASIESVLEKIRAE-FGEVDILVNNAGITR---DNLLMRMKDEEWNDII    107
KR1_ave           HLTITTCDTSTPRPTHNNSLNTIPP-QHPVTTVIHTGGILD---DATLTNLTPTQLNNVL    110
KR1_ery           RTTVAACDVTDRESVRELLGGIGDD-VPL-SAVFHAAATLD---DGTVDTLTGERIERAS    108
KR2_ery           RVSVHACDVSSREPVRELVHGLIEQ-GDVVRGVVHAAGLPQ---QVAINDMDEAAFDEVV    109
ChtnD_KR          QADYLPLDLGDAAATRAAIAALTAR-HGRVAALVHGAGALA---DRRIADKTAQDIETVF    153
ChtnB_KR          EVLALAGDIAADGVAHAAVQAALGR-FGRLDCVIHAAGVAGEAAHRPLLECGRAERAAIQ    109
                  .      :                        :.: ..

TropR-I_jweed     GTNFEAAYHLSQIAYPLL-KASQNGNVIFLSSIAGFSALPSVSLYSASKGAINQMTKSLA    186
TropR-II_jweed    SINFEAAYHLSVLAHPFL-KASERGNVVFISSVSGALAVPYEAVYGATKGAMDQLTRCLA    174
GlcDh_bacme       DTNLTGAFLGSREAIKYFVENDIKGTVINMSSVHEKIPWPLFVHYAASKGGMKLMTETLA    173
KR-fas_brana      DLNLTGVFLCTQAATKIMMK-KRKGRIINIASVVGLIGNIGQANYAAAKAGVIGFSKTAA    166
KR-fas_ecoli      ETNLSSVFRLSKAVMRAMMK-KRHGRIITIGSVVGTMGNGGQANYAAAKAGLIGFSKSLA    166
KR1_ave           RAKAHSAHLLHQLTQHTP-----LTAFVLYSSAAATFGAPGQANYAAANAYLDALAHHR-    164
KR1_ery           RAKVLGARNLHELTRELD-----LTAFVLFSSFASAFGAPGLGGYAPGNAYLDGLAQQR-    162
KR2_ery           AAKAGGAVHLDELCS--D-----AELFLLFSSGAGVWGSARQGAYAAGNAFLDAFARHR-    161
ChtnD_KR          RPKLDGLLTLLEALDPAP-----PARVLLFSSTAGFSGNAGQADYAMANEALAKLAFQLP    208
ChtnB_KR          AAKLDGTRRLAAALDGVA-----VRRVLLCSSLSTVLGGLGFAAYAAGNRALEVLAERQ-    163
                    :  .                    .:  .*            *.  :  :  ::

TropR-I_jweed     CEWAKDNIRVNSVAPGVILTPLVETAIKKNPHQKEEIDNFIVKTPMGRAGKPQEVSALIA    246
TropR-II_jweed    FEWAKDNIRVNGVGPGVIATSLVEMTIQ-DPEQKENLNKLIDRCALRRMGEPKELAAMVA    233
GlcDh_bacme       LEYAPKGIRVNNIGPGAINTPINAEKFA-DPEQRAD---VESMIPMGYIGEPEEIAAVAA    229
KR-fas_brana      REGASRNINVNVVCPGFIASDMTAKL---GEDMEKK---ILGTIPLGRTGQPENVAGLVE    220
KR-fas_ecoli      REVASRGITVNVVAPGFIETDMTRAL---SDDQRAG---ILAQVPAGRLGGAQEIANAVA    220
KR1_ave           -HT--HHLPATSIAWGTWQG----------------------------------------    181
KR1_ery           -RS--DGLPATAVAWGTWA-----------------------------------------    178
KR2_ery           -RG--RGLPATSVAWGLWA-----------------------------------------    177
ChtnD_KR          LRW--RGVRAVALAWGPWA-----------------------------------------    225
ChtnB_KR          -SR--DGAQWLALGYDGW------------------------------------------    178
                              :  .

TropR-I_jweed     FLCF-PAASYITGQIIWADGGFTANGGF-----    273
TropR-II_jweed    FLCF-PAASYVTGQIIYVDGGLMANCGF-----    260
GlcDh_bacme       WLAS-SEASYVTGITLFADGGMTQYPSFQAGRG    261
KR-fas_brana      FLALSPAASYITGQAFTIDGGIAI---------    244
KR-fas_ecoli      FLAS-DEAAYITGETLHVNGGMYMV--------    244
KR1_ave           ---------------------------------    181
KR1_ery           ---------------------------------    178
KR2_ery           ---------------------------------    177
ChtnD_KR          ---------------------------------    225
ChtnB_KR          ---------------------------------    178
```

**Figure S33. Sequence alignment of the ketoreductase (KR) domains from the chitinimine BGC with representative A- and B-type KR domains from other organisms.** B-type KR domains generate D-β-hydroxyl groups and their classification in *cis*-AT PKSs is based on the presence of a conserved Leu-Asp-Asp-like motif, which is highlighted in yellow.[56,57] The Rossmann fold involved in NADP(H)-binding GGxG(xxG) is marked in blue, the catalytic triad SYN/K in pink. [58] As a reference, the following KR domains were used: TropR-I_jweed, tropinone reductase-I from jimsonweed (*Datura stramonium*) (type A); TropR-II_jweed, tropinone reductase-II from jimsonweed (type A); GlcDh_bacme, glucose dehydrogenase from *Bacillus megaterium* (type A); KR-fas_brana, β-keto ACP reductase from *Brassicanapus* (type B); KR-fas_ecoli, β-keto ACP reductase from *E. coli* (type B); KR1_ave from the avermectin BGC from *Streptomyces avermitilis* (type B); KR1_ery from the erythromycin BGC from *Saccharopolyspora erythraea* (type B); KR2_ery from the erythromycin BGC from *Saccharopolyspora erythraea* (type A).

```
ChtnE_DH      REVLFDEADVMEFAEGRVANVLGPHYAPVDALPRRVRIPGPPFMAVSRITHLSGTYGQLE  60
ChtnE_DH      GSRIRTEYDIPDNAWNAVDGQASYLSLDAQGVLFLAGWLGIDFENRGNRAYRWLDAQLTY  120
ChtnE_DH      LGPMPRAGQRVEYDIHIHQAFRNGDATLFRTDFLARVDGRPVLKIDHCTAGFFTYEELAR  180
ChtnE_DH      GAGITDQHRNRRPPAAQPFAAPLAPPGRPLERADLNALARGAIAEVLSPAHAAGGRNPAL  240
ChtnE_DH      RIPPPVIQFIDRVVRIDAAGGACGLGRSEAEWRIDPQHWAIRAHFKDDPVFPGPCMLEGA  300
ChtnE_DH      VQLLQLHALALGLQTAVAGARFQPVAGRPILVRFRAQVVPRNQLFTYRADIVEIGLGPEP  360
ChtnE_DH      YLIADIDLIDEGRVAGRVEGLGVRLVGQP                                 389
```

**Figure S34. Sequence analysis of the dehydratase (DH) domain within ChtnE.** The domain contains a double-Hotdog fold, with active site aspartate residues (Dxx(x)H) and catalytic histidines located at either a HxxxVxxxP motif or a HxxxGxxP motif.[59]

```
ChtnE_KS5      ------------------------------------------------------------ 0
ChtnE_KS4      --IIGLGCLVPDAGDPATFWANLCAGRRSIRDADARDWGVEPTRFLAPGRGVADHVSSLE 58
ChtnD_KS3      IAVIGMAAMLPKAHDLAEYWRNIVDGTDCLEPLPADRWRSEHYYDADPKA---ADRAYAE 57
ChtnA_KS1      IAIIGAACRFPGADSPDRLAELLFDGREAIGPVAALR---PA-----------IAAGGIE 46
ChtnB_KS2      IAVVGMAGRFPGAADVEALWQLLLEGRSGVREIGRDEALADG---ADPAL--LDHPGYVP 55


ChtnE_KS5      ---------AIDALRLRVPPNDIDRMYPQQLLMLAVGDAALRDAGIEPGSR--------- 42
ChtnE_KS4      LG--KPRDFVFDPSGYLLPADFLAAQDRCIQWPLEAARQALLDAGLQPGE-------DLG 109
ChtnD_KS3      RGGF-VPDLWFDPLRYGMPPNTLASTDAAQLYALAVGRQALLDAGYDPDPAGTGRRLPAG 116
ChtnA_KS1      RAGLIAAPELFDPQFFGIAQREADQMDPQQRLALELAVEALEAAGLPRAG------LAGS 100
ChtnB_KS2      FAGTLDGIDAFDERLFGYSPADAALIDPQGRIFLECAHEALASAGIDPAR-------CGG 108
                            :*                     *  .  **  **

ChtnE_KS5      -TAVIVAGAMDHA-------GHRLMARWEAAWRLEDNLDAAGFDLSAEERTQLAALVRDA 94
ChtnE_KS4      RVGLVLGSYAWAAGSASDALTR---PLYDQALAQ------AFA---EAAGDPDRDPLRLT 157
ChtnD_KS3      RAGIVLGVSGNTMKLSSEMGKRADIAKWIDALRQ------AGA---GAA---LIETVAGA 164
ChtnA_KS1      RTGVYLGISTYDYSRLQMRRGD-----------------------GGE---L------- 126
ChtnB_KS2      RIGVYAGASVSSYALAALRGPA-------------------LA---DTE---L------- 136
                    .:  .

ChtnE_KS5      LHQPV---DAVVMLSYVGSLLASRIAATWDLSGPAFMLTGDETGALRALDLGAKLLASDE 151
ChtnE_KS4      VGRPTP--STHPESARISGGITTTVARALGLGGPRYAIDAACATSLYAIHLAALHLAAGE 215
ChtnD_KS3      MRRHYPDWTEDTFPGFLANLVAGRIANRFGLGAASHTVDAACASSLAAVRLACLELRSGA 224
ChtnA_KS1      ----Y------AGTGNAFSIAANRISYWLNLAGPSMAVDTACSSSLTAVHLAVRALRAGE 176
ChtnB_KS2      ----FR-----ALFANDKDYLASRVAYKLGLKGPAVGVQTACSTSLVAVAMAVRALRSGE 187
                                .   .  :  ::   .* .    :    : :* *: :.   * :.

ChtnE_KS5      ADAVLVGAVDLAGAIENLMVRQAQG-------------VDRAAPVGEGAVAVVLEPAAAV 198
ChtnE_KS4      ADAMLVVAANAFDTLYATFGFAATQALPDGSPNRPFDARSDGVAPADGAVALVLRRSGSR 275
ChtnD_KS3      ADLMLTGGVDTDNSNVAFLSFSKTPALSRSGRVRAFDAAADGTMISEGVGMLVLKRLDDA 284
ChtnA_KS1      IDLALVGGVGLLLSGELMQVFAGAGMLAPDGRCKTFDAAADGYVRGEGGGMVVLRRAAEA 236
ChtnB_KS2      CDAVLAGGASVSVPQRVGYLYEDGSILSPDGRCRAFGIDAAGTVPGNGVGVVVLKRLSRA 247
                   *  *. ...                               .   .:*   :**.

ChtnE_KS5      -------------RERGGAAYAEWRGAGFGEQP-------------AEAARQAHAATGI 231
ChtnE_KS4      GHERPGLSGREGPDLCGREAYGVIRAIGLSSDGRG-QTLTAPNPKGQQLACERAYARSGI 334
ChtnD_KS3      -------------LAAGDRVYGLIRGLGASTDGAG-GAIFAPHAAGQARALEAAYADAGI 330
ChtnA_KS1      -------------AAAGDRVLALIAGSAVNQDGR-SNGLTAPSGPAQSAVLRAALADAGL 282
ChtnB_KS2      -------------LADGDPIRAVIRAVALNNDGADKVGFSAPSVGGQEEVLQAALREAGL 294
                                  *    .   . . . :                 . . *   :*:

ChtnE_KS5      AAAEPGLVEAGDTLPA---AAEL------------AGQPALASAAAVFGHARMAAPLLAA 276
ChtnE_KS4      DPASVAYVECHATGTKLGDRVELETVGRVFGP-----GQPVGSVKSNVGHLLTAAGVAGL 389
ChtnD_KS3      DPASVGLIEAHATGTVVGDAVEIESLESVLGG--AAAPVALGSVKAQIGHAKAAAGAASL 388
ChtnA_KS1      APAEVDAVELHGTGTPLGDPIEAQALGEVY-AAGRAAPLAVGSIKTNIGHLEAAAGIAGL 341
ChtnB_KS2      DAADIGYVETHGTGTRLGDEVELSALAGAFGGAGQGARCLIGSLKSNLGHLDAAAGVAGL 354
                    *.   :*   *        *                  :.*  : .**   **    .

ChtnE_KS5      LHAALALNARQLPAWAGWREAAAAHSLDGRAGYVPTEPRPWLRPRHGCRIAAVLARDGDG 336
ChtnE_KS4      VKTLFALREGMIPATVGIGQSLAAEIAGQ--PRILTEPTPWP---GAQRRAAVNAFGFGG 444
ChtnD_KS3      IKTLLALYHKVIPPTLGVSAPNPRLDPRERPFYLPRRARPWLAPAAGPRRAGVSVFGFGG 448
ChtnA_KS1      IKAALALAARRLPPSLNFSRPNPDIDLAALGLAVATEAVPLD-PIGRPARVGVSSFGFGG 400
```

```
ChtnB_KS2      IKAVLTVERGIVPASLHVEQPNALLAAGGSRFALATATVAWP-DDGRPRRAGVSSFGIGG  413
                  ::: :::    :*                     :                ..*     . .*

ChtnE_KS5      SAARALLAEAP   347
ChtnE_KS4      VNAHLVVD---   452
ChtnD_KS3      ANVHLALEE--   457
ChtnA_KS1      SNAHVVLEAA-   410
ChtnB_KS2      TNAHCIVEQP-   423
                    .:  :
```

**Figure S35. Sequence alignment of ketosynthase (KS) domains from the chitinimines BGC.** The catalytic triad highlighted in yellow is present in all KS domains, except in ChtnE_KS5, which is a chain-length factor.[60]

```
ChtnD_ACP6      --LLLQTVADKTGYPVEMLSLDMRLEGDLGVDSIKRVEILAAMRDALGLAAD—GAGDGL  56
ChtnD_ACP4      ---LLRTVADKTGFPVELLAPGMRLEGDLGVDSIKRVEILAALRDALGLAAADGQAGEAL 57
ChtnD_ACP3      ---LLRTVADKTGFPVELLTLEMKLEADLGVDSIKRVEILAAMRDALGLTAAAGD-GEAL 56
ChtnD_ACP5      ---LLRTVADKTGFPVELLTLEMKLEADLGVDSIKRVEILAAVRDALGLTTAAGD-GEAL 56
ChtnA_ACP1      ---VVEQIARALGEAPERLPLDKPF-IEMGADSVMMAEAMRAIQARYGVRISAR---QLL 53
ChtnF_ACP7      -DWLMAQVAAQLEVEADDIDPRRTF-ESYALDSARALLVLTRLEARLGLRLSPT---LIW 55
ChtnC_PCP4      -EALAAVWRAVL--QCGELALEDDF-YALGGDSIMAMQVSMRLTRQ-GWTLRPQ---DML 52
ChtnA_PCP1      ETALLDYVRGTL--QVALRGIDHDF-FAAGGQSLAATQLIGWVQRQWSVKPALK---DFF 54
ChtnB_PCP3      ERRIAAIWCEVL--GLPAVDAARNF-FEAGGNSLLLMQVHARLKQAFSPAPRLA---DLF 54
ChtnB_PCP2      EQAIAALWQALL--GVERVGRHDDF-FALGGHSLLATRAAARLGRRFGLRLPMA---ALF 54
ChtnC_PCP5      ELELARLWEDLL--GLAPIGRDDGF-FALGGHSLLVLELMARLRARFGRAVPFA---AFL 54
ChtnB_ACP2      EAAVAQAIADTL--ALAAVGPDDDF-FALGGDSLVATRVIARLRGATGLPLSVG---LVL 54
                      :                    :    . .*         :    .

ChtnD_ACP6      RGAVTLAELAER----      68
ChtnD_ACP4      RNAATLADIAA-----      68
ChtnD_ACP3      RGAATLGEIAAL----      68
ChtnD_ACP5      RGAATLGEIAALL---      69
ChtnA_ACP1      NELDTVDALSAHL---      66
ChtnF_ACP7      N-YPTIEALAGRLAQ-      69
ChtnC_PCP4      R-QPTLAAQCALMRR-      66
ChtnA_PCP1      E-LPTVACLAALIEA-      68
ChtnB_PCP3      R-FPSVAALAAFLGRE      69
ChtnB_PCP2      D-APVLAALAARIEAA      69
ChtnC_PCP5      R-APTVAGLAALLG--      67
ChtnB_ACP2      Q-APTVRLLAAAVR--      67
                        :   .
```

**Figure S36. Sequence alignment of acyl (ACP) and peptidyl carrier protein (PCP) domains from the chitinimine BGC.** The highly conserved 4'-phosphopantetheine binding motif (LGG(H/D)S(L/I)) is highlighted in yellow.[61]

```
ChtnC_E         --LPVTPIQAWFFALELAHPQ---HWNQAVRLALDP-AAAGRLEPALAALEAAHEALRLR 54
ChtnA_C1        ATLPLSDSQRQIWLATELDPAGAGAYCETVALEVDGELNPALLERALAQLALRHEALRTV 60
ChtnC_C5        DLLPVTPQQRGMLLESQAHAG-LGLHVEQFVATFDGAFDRAALEAAWARLVARHDTLRSG 59
ChtnC_C4        APVPLSIEQRAVWLAEQHGG--GRAFLIPGALRLRGRLDAAALRLALQALVDRHEAFRTA 58
ChtnB_C2        -----TPAQQRLWLLCGLGED-AANYVIAGALRLQGALDPARLEAALNDCLARHESLRTG 54
ChtnB_C3        -PAPLSASQLGLWFVQQLDPA-STAYVLSGAIRIDGALSRSLLSRTLDLLQARHHALRTR 58
                      :  *  .                     .      . *  :       *.::*

ChtnC_E        FAPGETGWTMRVAPAGAPPLRRVRAEDA------GEAL-VQIEAAQRSLDLAAGPVWRAL  107
ChtnA_C1       LDAGG--AGQTVQPALKPPLSYGE---------HDDVAGWLRGFVEAAFDPAAGGPLRAA  109
ChtnC_C5       FAWRQEHAPLCLVHARAEP-AWQHLDWRGEAVDEARIAAWLERDRLAGFDGARP-PLRFA  117
ChtnC_C4       IRLVGDEPMQCVQSAVHFALPEHDLSALMPAERDGALERLLAAEAARPFELARAPLLRAV  118
ChtnB_C2       FAEIDGVPQQAIEPQAALALPLTELDALPAEARLEAALATAGALARQPFDLARPPLLRAR  114
ChtnB_C3       FDAAGGVPSQTVLAPSGIALPIDDLSRLAPAMREAELQRRLDAEARRPFELPGPPPVRAR  118
                 :          :                                     ::      *

ChtnC_E        LIDGPHDGWPHLVLIAHHLVVDGVSWRLLVDDLAQALAGGE-----PAPAALGYADWAAH  162
ChtnA_C1       LLKRSDGRH-VLALRAHHVLVDGWSLALIVDELGRLYHGGA-----ALAEAQPFRRQLDW  163
ChtnC_C5       TLRLDASRW-LFVWTYHHALLDGWSVARLLAEALAPA---------ADEAPPAARDHARW  167
ChtnC_C4       LVRLAAEEH-VLALTPHHIVADGWSIDVMVRELSLFYRPDGSP-PPPLPAPLGYPDFAAW  176
ChtnB_C2       LFRLGADDW-LLALAIHHIVCDGPSLGILIADLAAAYAGRGQGRPLPAAPALQFADFAEW  173
ChtnB_C3       LLVLGPDCH-VLSLSLHHIVADGWSLGLVWRDLVQGYRALREGGAPDWTPLPQYADYARG  177
                  .         :    ** : ** *   :  :

ChtnC_E        LAGLPPQPPR--------AAVPPAPPPIARPDGDDFEAQTRI----------ATLALPMD  204
ChtnA_C1       LAGAQ----DEAAERFWRDRVAEPPAPLALPGQRLFDAA---ARPRWEGE-RVRLALPAE  215
ChtnC_C5       LAGQD----RAAAAAFWRAELAGAEVP--TPAGRVDP-----ALAPGRGHADRCRRIDAA  216
ChtnC_C4       QAGRIAAGADAADLDYWRATLAELPPPLELPAARQAAAGTDGIAAGYAGA-AVQATLPAA  235
ChtnB_C2       QQEQLARPETERLLAAAAARLAGVPD-LLVPTDRPRP-----ALRAGRGA-RHAFALDAV  226
ChtnB_C3       EAARLAAPAAAAELDYWRRALDALPP-LELPTDFARP-----PLPGYRGA-QYRFTVPAR  230
                                       :         *                         :

ChtnC_E        ATAALLGPANQPYRSEPTELLLAGLLLGLQAAHGRSALAVALERHGRDVDGVELAGTVGW  264
ChtnA_C1       L-AEKLAALAAGRRVTPFTAALALTLGWLHRLCDRDDILIGVPAHGRPD---GLERMVGQ  271
ChtnC_C5       T-VAQLETLARRHRLTPALLAQGLWGLALAWASGRREVTLARTVAGRPAEVDGSERWVGL  275
ChtnC_C4       T-ARGLRRLAAESGTTLFSALAALFAALLQRYGGRRELVLGTASAGRGRI--ELEEVVGL  292
ChtnB_C2       L-MDAVTLRARALGSTPFAVLLAAWGIVLAGWSGQDDFAIGTPVSGRADP--ALAEVVGL  283
ChtnB_C3       L-CGSADRLARAAGASRFMVLLAAFQALLARWSGQRDFAVGVPVSGREAL--DWQETVGC  287
                                         .     *    .:  . :.    **          **

ChtnC_E        FTAIVPLLLAL    275
ChtnA_C1       AVQLMPLRSRI    282
ChtnC_C5       FINSLPLRLSL    286
ChtnC_C4       FAGRLPLRLDF    303
ChtnB_C2       FAETAALRFRC    294
ChtnB_C3       FVNTVAIRAEL    298
                       :
```

**Figure S37. Sequence alignment of the condensation (C) domains from the chitinimine NRPS modules.** The active site HHxxxDG motif is highlighted in yellow and present in all C domains.[62] E domain-specific signature motifs are highlighted in green.

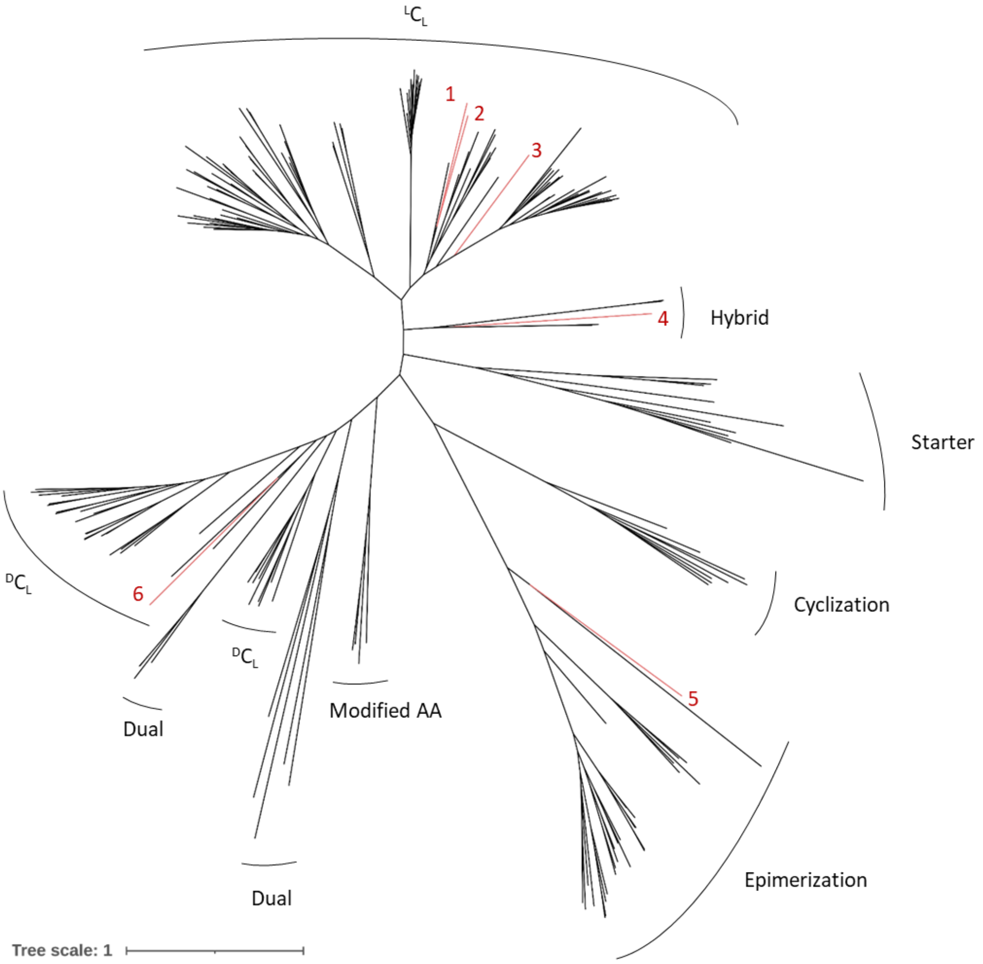


**Figure S38. Neighbor joining phylogenetic tree of condensation (C) domains from the chitinimine BGC and reference C domains from the NaPDoS database.** The phylogenetic tree was generated with NaPDoS2 and visualized with iTOL. Red branches represent C domains from the chitinimine BGC in *C. koreensis*. Functional classifications are based on NaPDoS annotations: 'Starter' C domains are typically not present in the first module of an NRPS, but when present, link an amino acid to a fatty acid, polyketide, or another molecule; '$^{L}C_{L}$' domains catalyze peptide bond formation between two L-configured amino acids; '$^{D}C_{L}$' domains couple a D-configured amino acid to an L-configured amino acid; 'Cyclization' domains catalyse chain elongation with cysteine, serine or threonine residues, followed by a two-step cyclodehydration reaction to form five-membered thiazoline or (methyl)oxazoline rings, respectively; 'Epimerization' domains act on elongated PCP-bound peptidyl intermediates and isomerize the $\alpha$-position of the C-terminal amino acid residue; 'Modified AA' domains modify the newly incorporated amino acid residue; 'Dual' domains have both condensation and epimerization abilities; 'Hybrid' domains add an amino acid to a growing polyketide chain. 1: ChtnB_C3 ($^{L}C_{L}$); 2: ChtnC_C4 ($^{L}C_{L}$); 3: ChtnB_C2 ($^{L}C_{L}$); 4: ChtnA_C1 (Hybrid: immediately downstream of a PKS module); 5: ChtnC_E (Epimerase); 6: ChtnC_C5 ($^{D}C_{L}$).

| | A | B |
|---|---|---|
| *Enterococcus faecium* DSM 25390 | 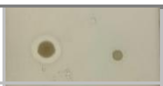 | 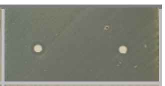 |
| *Staphylococcus aureus* DSM 21979 | 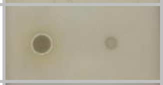 | 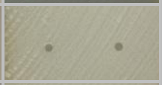 |
| *Staphylococcus aureus* RN4220 | 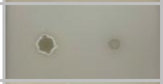 | 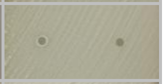 |
| *Staphylococcus aureus* ATCC 6538 | 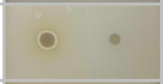 | 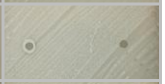 |
| *Staphylococcus aureus* StaAu068 | 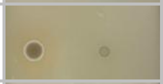 | 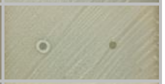 |
| *Staphylococcus aureus* Sa9 | 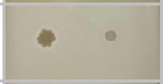 | 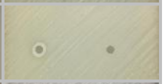 |
| *Staphylococcus capitis* StaCa010 | 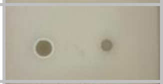 | 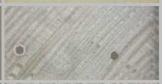 |
| *Staphylococcus haemolyticus* StaHa024 | 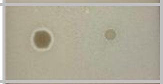 | 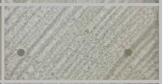 |
| *Staphylococcus hominis* StaHo017 | 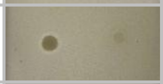 | 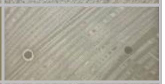 |
| *Staphylococcus lugdunensis* StaLu018 | 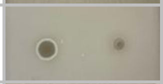 | 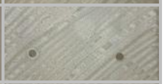 |
| *Bacillus cereus* DSM 31/ATCC 14579 | 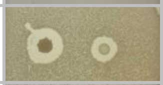 | 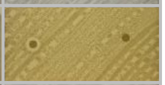 |
| *Bacillus subtilis* ATCC 9799 | 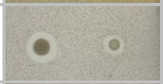 | 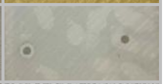 |
| *Mycobacterium smegmatis* MC2-155 | 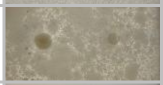 | 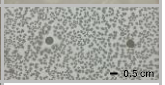  |
| *Salmonella enterica* serovar Newport C487 | 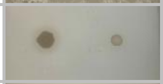 | No bioactivity |
| *Salmonella enterica* serovar Typhimurium ATCC 14028 (+GFP) | 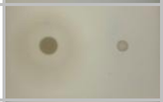 | No bioactivity |
| *Salmonella enterica serovar* Enteritidis ATCC 13046 | 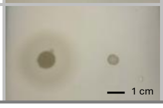  | No bioactivity |

**Figure S39. Antibacterial activity of the chitinimines and the *C. koreenis* DSM 17726 WT and Δ*chtnA* mutant.** (**A**) Soft agar halo assays comparing the antibacterial activity of the *C. koreensis* WT (left) and Δ*chtnA* mutant (right) strain. (**B**) Plate lawn assays with 10 µL of a 1 mg/mL purified chitinimine mixture (left) and a DMSO solvent control (right).

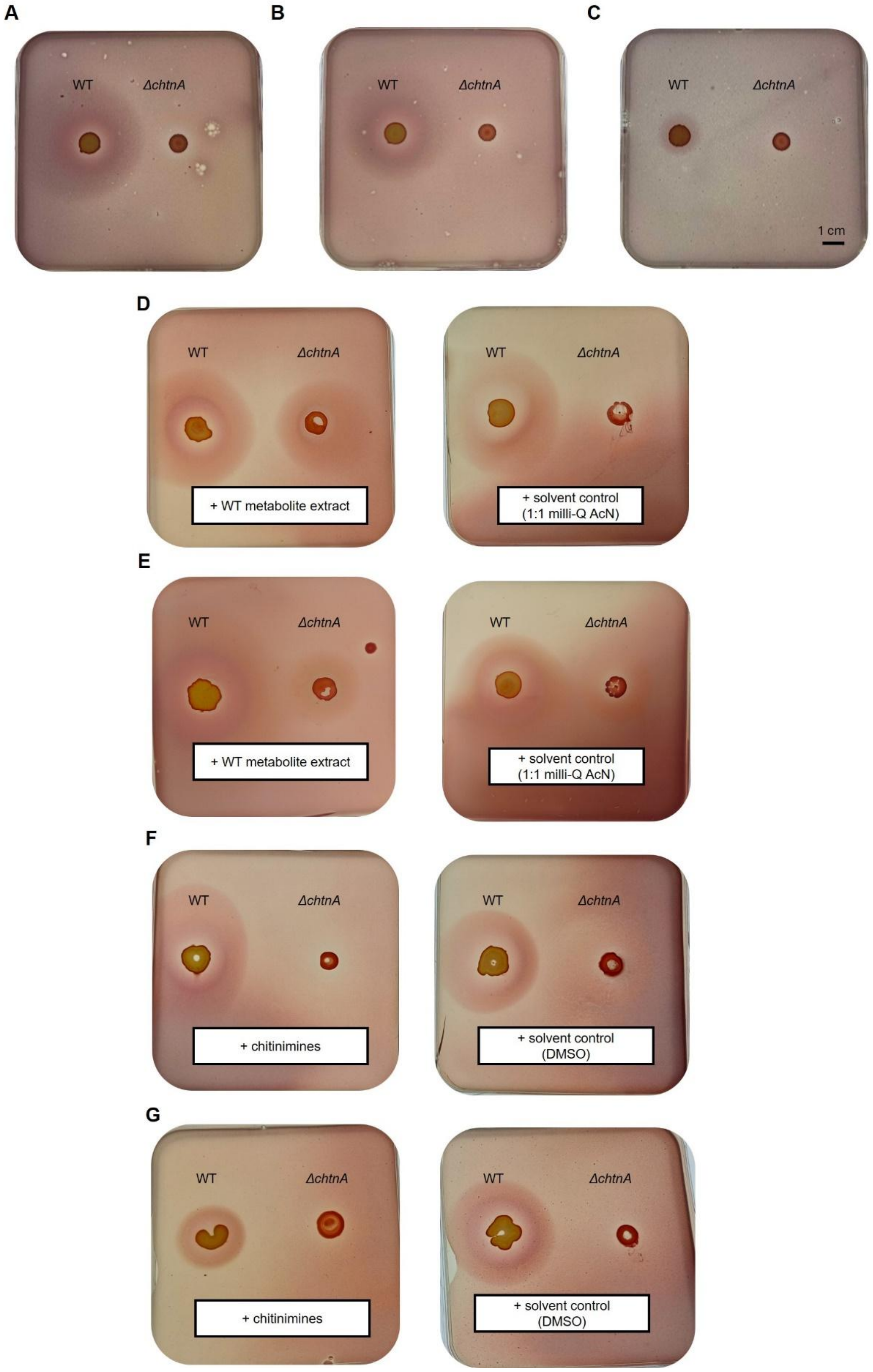


**Figure S40. Growth-promoting effects of *C. koreenis* DSM 17726 WT and the Δ*chtnA* mutant on *Salmonella* species.** WT (left) and Δ*chtnA* mutant (right) cultures of *C. koreensis* were spotted onto BSM agar plates supplemented with glucose as a sole carbon source. Following incubation for four days at 28°C, the cells were inactivated by chloroform vapours and overlaid by soft agar inoculated with *Salmonella enterica* serovar Typhimurium ATCC 14028 (**A**), *Salmonella enterica* serovar Enteritidis ATCC 13046 (**B**) or *Salmonella enterica* serovar Newport C487 (**C**). The soft agar was also supplemented with iodonitrotetrazolium chloride to enhance visualisation of *Salmonella* growth. After 24 hours of incubation, growth-promoting effects were observed in the presence of WT *C. koreensis* DSM 17726. Chemical complementation of the KOs of *S. enterica* serovar Typhimurium ATCC 14028 (**D**) and *S. enterica* serovar Enteritidis ATCC 13046 (**E**) was achieved by supplementing the KO cells with 10 µl of ethyl acetate extract of WT *C. koreensis* dissolved in 1:1 milli-Q acetonitrile. Only providing the chitinimines (dissolved in DMSO) did not result in recovery of the growth promotion effect of KO *C. koreensis* against *S. enterica* serovar Typhimurium ATCC 14028 (**F**) or *S. enterica* serovar Enteritidis ATCC 13046 (**G**).

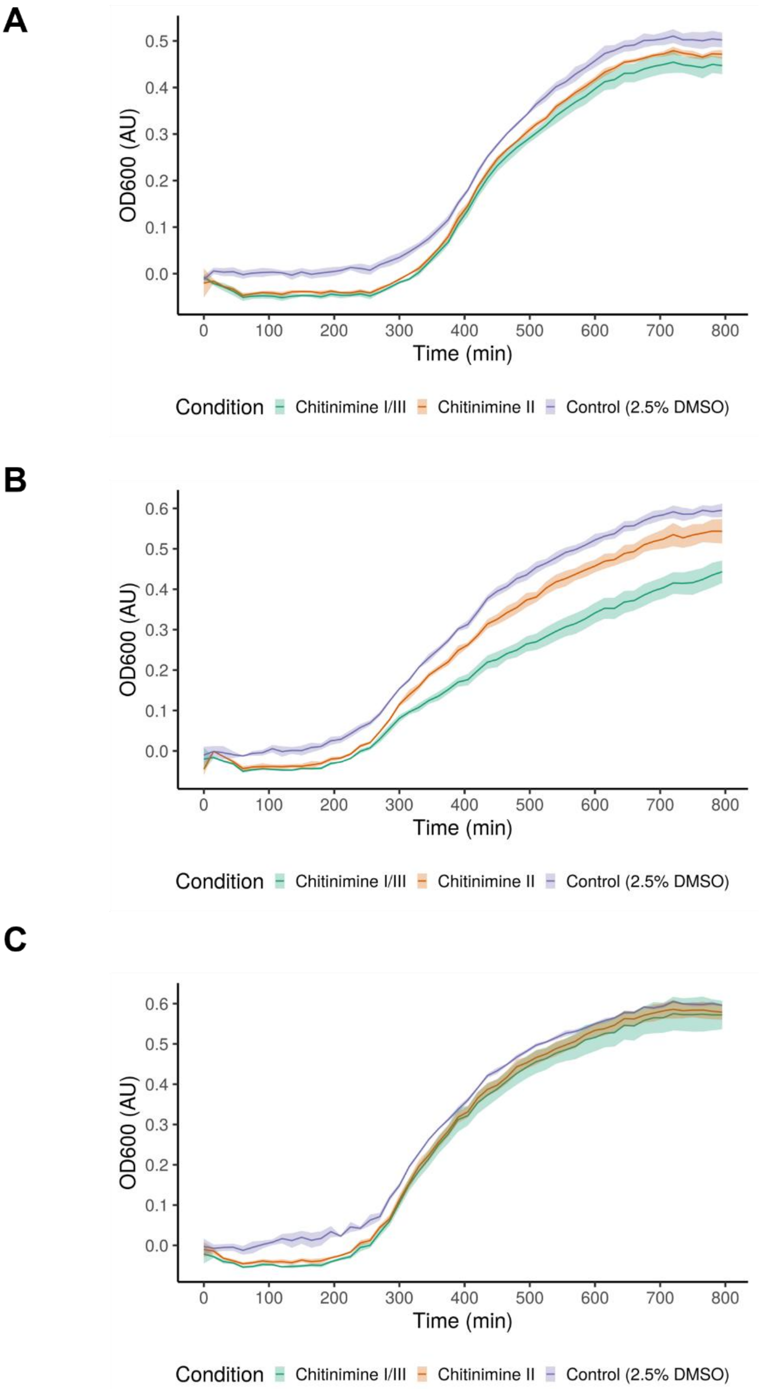


**Figure S41. Growth curves of *Salmonella* strains in the presence of chitinimines.** *Salmonella enterica* serovar Newport C487 (**A**), *Salmonella enterica* serovar Typhimurium ATCC 14028 (**B**), and *Salmonella enterica* serovar Enteritidis ATCC 13046 (**C**) were exposed to chitinimine I/III or chitinimine II (125 µg/mL in 2.5% DMSO), and the optical density at 600 nm was monitored for 13 hours. No growth-promoting effect was observed compared to cultures supplemented only with 2.5% DMSO, indicating that the chitinimines alone do not have growth-promoting activity. Growth curves represent the mean of three replicates, while shaded areas represent standard deviations.

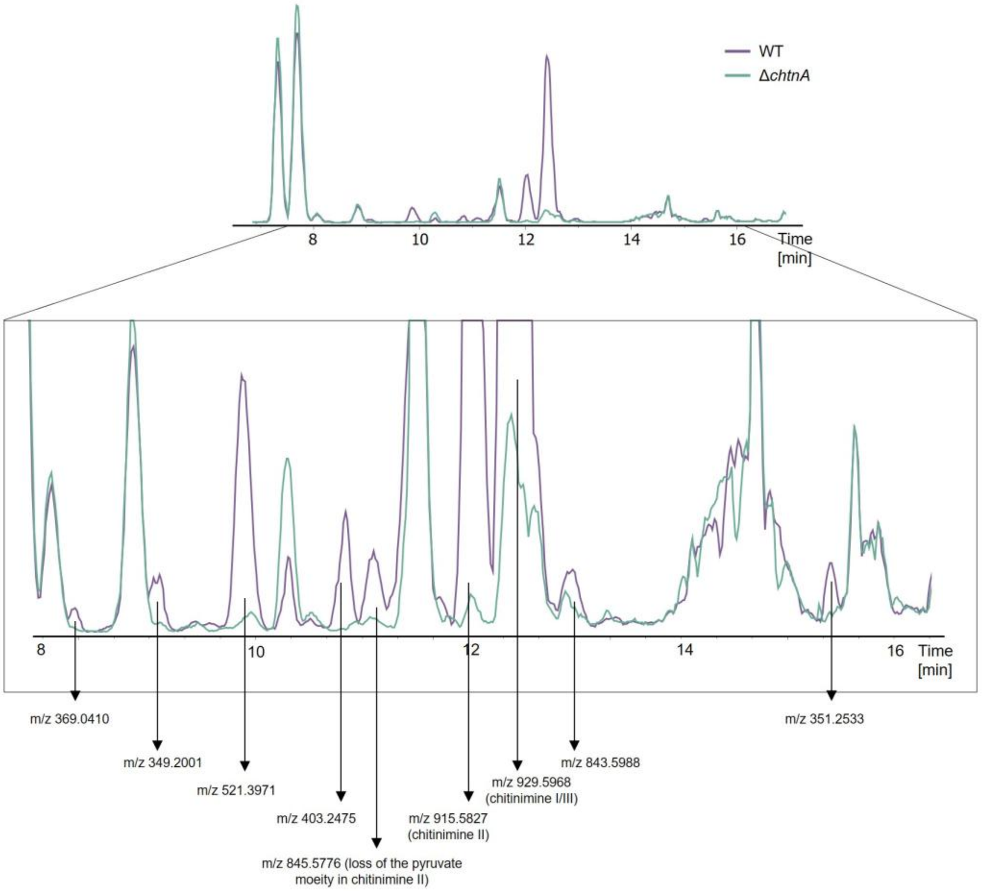


**Figure S42**. **Comparative metabolic profiling of *C. koreensis* wildtype and the Δ*chtnA* mutant.** A chromatogram spanning peaks corresponding to metabolites whose production is altered in the mutant is shown, alongside the *m/z* value referring to the most intense ion at the corresponding differential chromatographic peak.

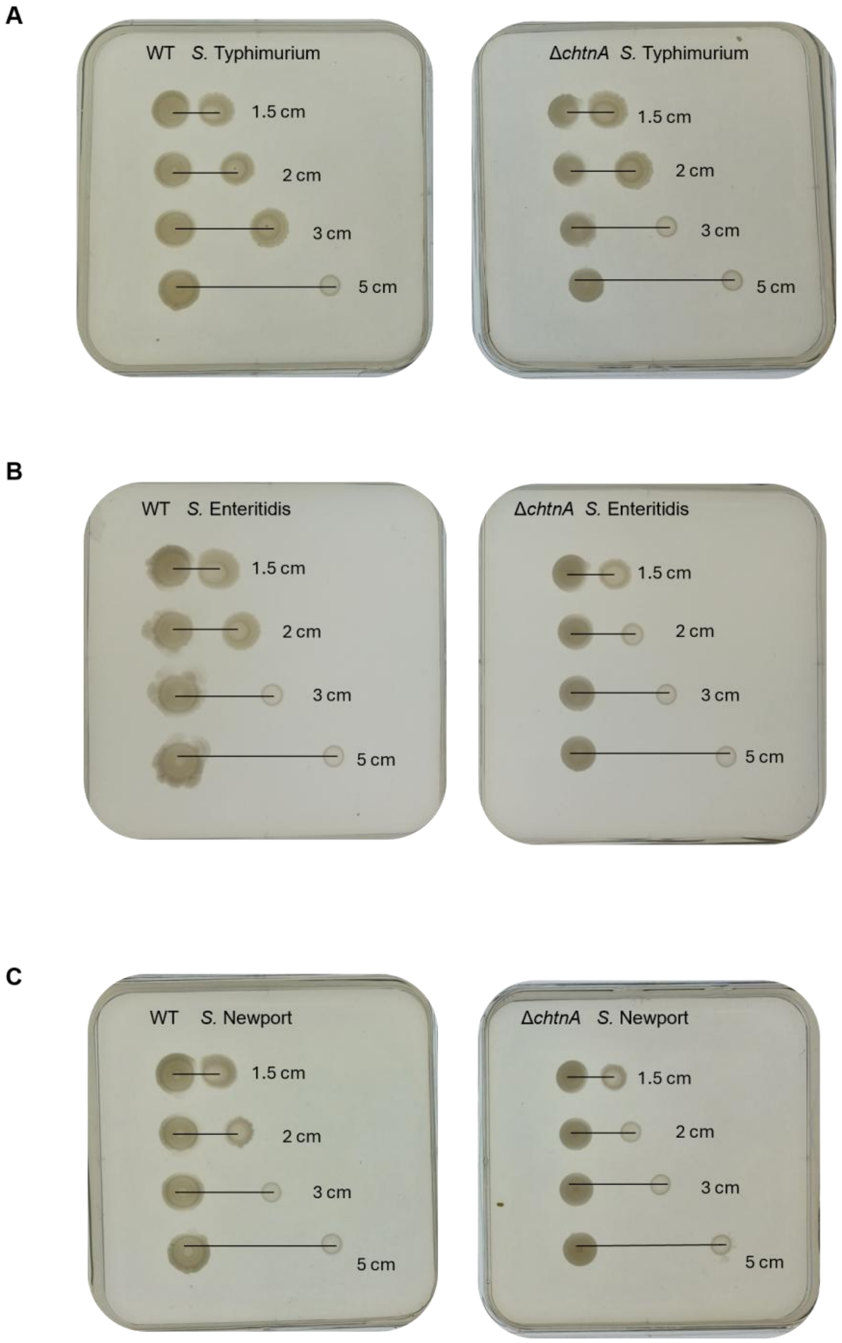


**Figure S43. Promoted growth of *Salmonella* species in presence of *C. koreensis*.** WT (left plate) or KO (right plate) *C. koreensis* cultures were spotted onto BSM agar plates supplemented with glucose as a sole carbon source. Following incubation for four days at 28°C, cultures of *Salmonella enterica* serovar Typhimurium ATCC 14028 (**A**), *Salmonella enterica* serovar Enteritidis ATCC 13046 (**B**) or *Salmonella enterica* serovar Newport C487 (**C**) were spotted on the same plates at variable distances. After 48 hours of incubation, the growth of *Salmonella* species was enhanced at closer distances and in presence of WT *C. koreensis* in comparison to the Δ*chtnA* mutant.

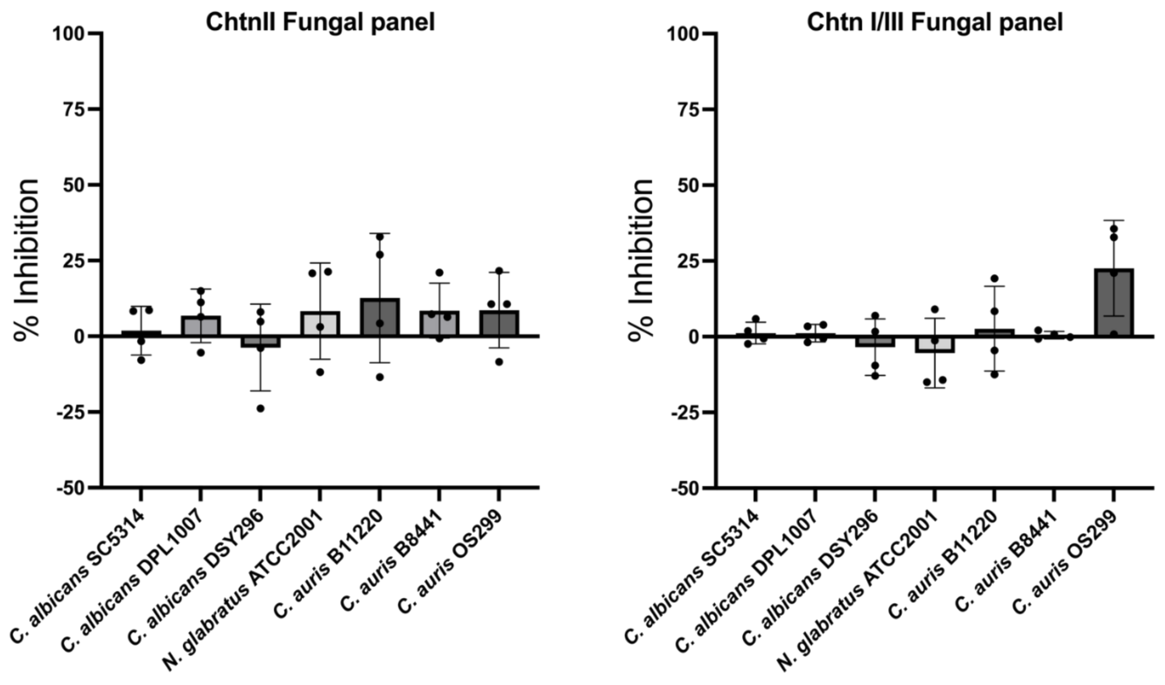


**Figure S44. Antifungal activity of the chitinimine II (left) and chitinimines I/III against a panel of *Candida albicans*, *C. glabrata* and *C. auris* strains.** Growth inhibition (%) was calculated relative to the untreated control for each strain.

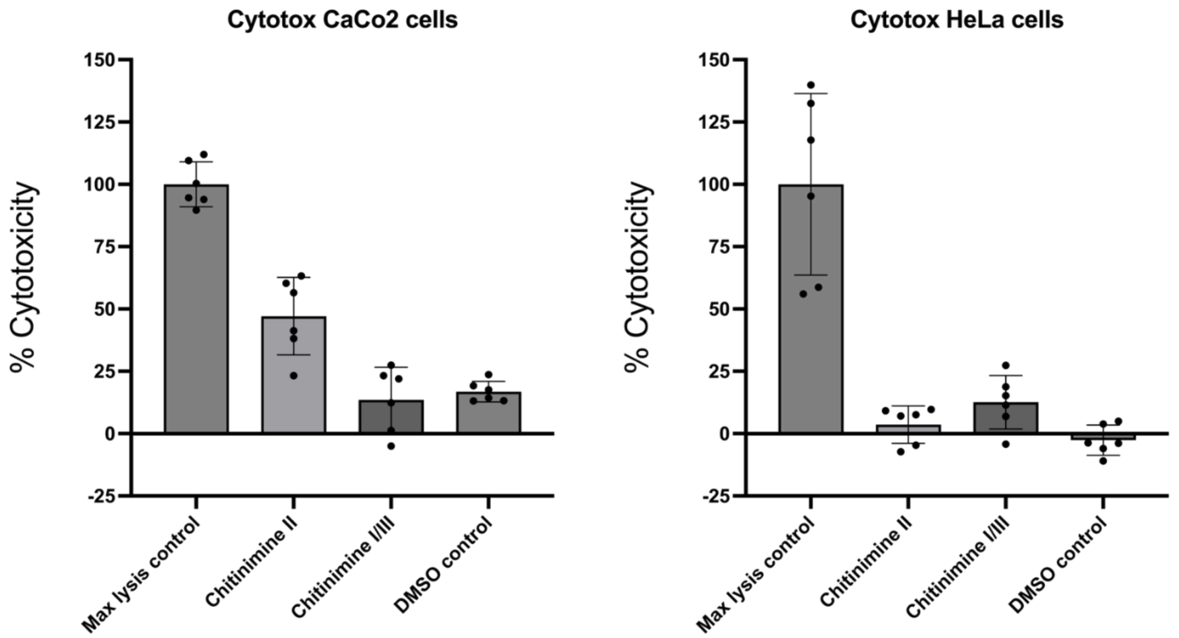


**Figure S45. Cytotoxicity of chitinimine I/III and chitinimine II against HeLa and CaCo-2 cells, as determined by the lactate dehydrogenase (LDH) cytotoxicity assay**. Maximum LDH release was obtained by lysing cells with the supplied lysis buffer ('max lysis control'). Cells treated with 2% DMSO served as negative controls. Error bars represent the standard deviation of three independent experiments.

## Supplementary Tables

**Table S1**. **Summary of genome mining results for candidate clusters encoding hybrid PKS-NRPS-PUFA synthase-like biosynthetic pathways.** Compilation of NCBI GenBank accession numbers, species and strain names, bacterial orders, if the clusters were retained or discarded, and – if applicable – the reason for exclusion. For each entry, an image of the corresponding cluster-containing region from antiSMASH is included.

| GenBank accession no. | Species and strain | Order | Discarded? | Reason for exclusion |
|---|---|---|---|---|
| **Gram-negative bacteria** | | | | |
| AP009552.1 | *Microcystis aeruginosa* NIES-843 | Chroococcales | No | / |

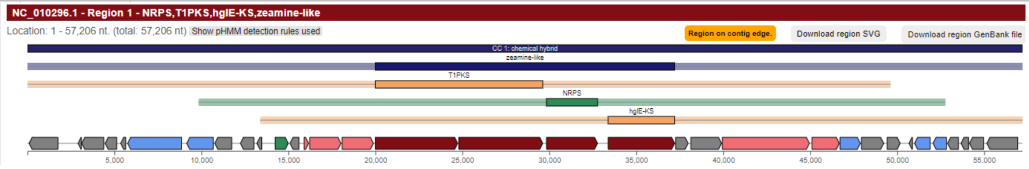


| | | | | |
|---|---|---|---|---|
| CP097576.1 | *Microcystis aeruginosa* Chao 1910 | Chroococcales | No | / |

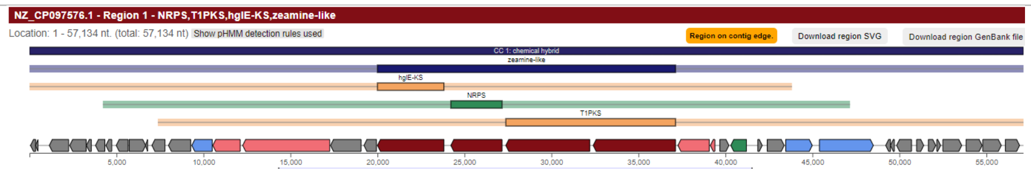


| | | | | |
|---|---|---|---|---|
| CP003552.1 | *Nostoc* sp. PCC 7524 (ATCC 29411) | Nostocales | Yes | PfaBC homolog misannotated as T1PKS |

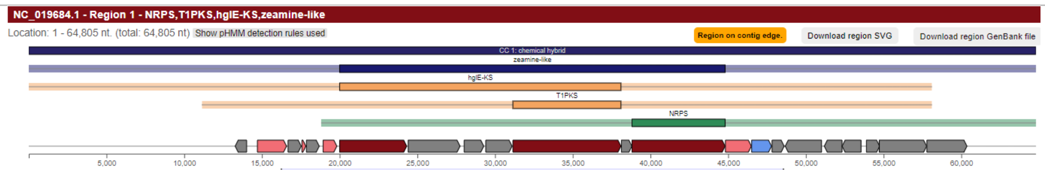


| | | | | |
|---|---|---|---|---|
| AP018216.1 | *Trichormus variabilis* NIES-23 | Nostocales | No | / |

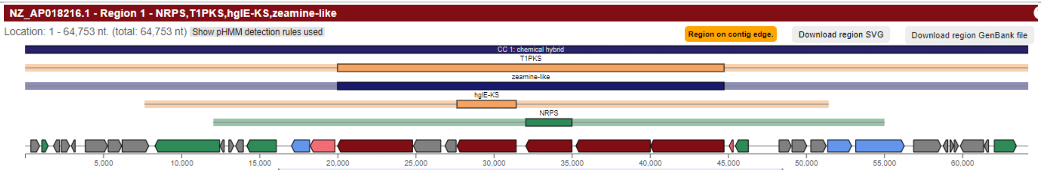


| | | | | |
|---|---|---|---|---|
| AP018316.1 | *Dolichospermum compactum* NIES-806 | Nostocales | Yes | 85% of genes from heterocyst glycolipid BGC share at least 30% identity to this *pfa* operon and 100% of genes from the anabaenopeptin NZ857/nostamide A BGC share at least 30% similarity to the NRPS genes. |

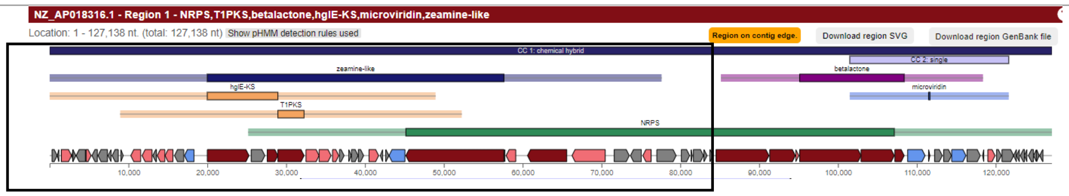


| | | | | |
|---|---|---|---|---|
| BDUD01000001.1 | *Nostoc commune* NIES-4072 | Nostocales | No | / |

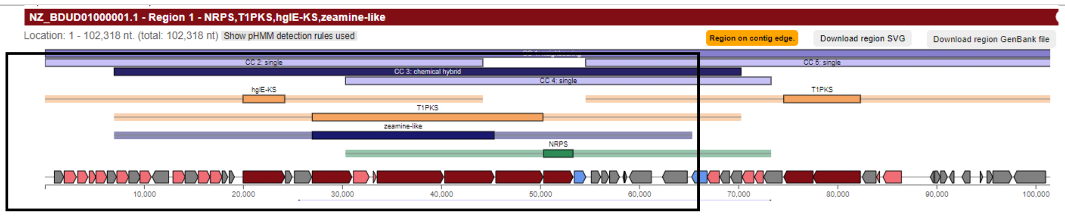


| | | | | |
|---|---|---|---|---|
| CP024792.1 | *Nostoc flagelliforme* CCNUN1 | Nostocales | No | / |

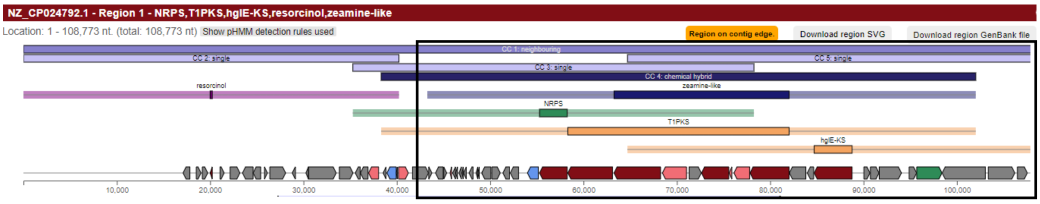


| | | | | |
|---|---|---|---|---|
| CP045227.1 | *Nostoc sphaeroides* CCNUC1 | Nostocales | No | / |

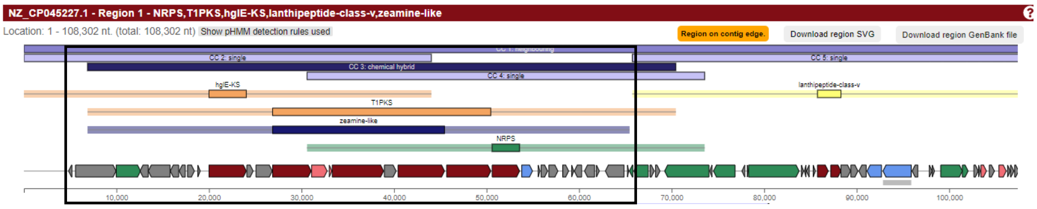


| | | | | |
|---|---|---|---|---|
| CP034058.1 | *Anabaena* sp. YBS01 | Nostocales | No | / |

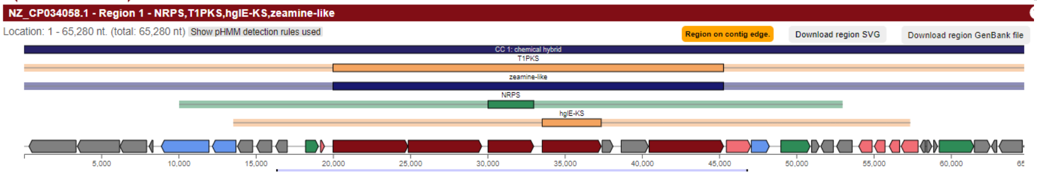


| | | | | |
|---|---|---|---|---|
| JH992901.1 | *Mastigocladopsis repens* PCC 10914 | Nostocales | Yes | 85% of genes from heterocyst glycolipid BGC share at least 30% identity to this *pfa* operon |

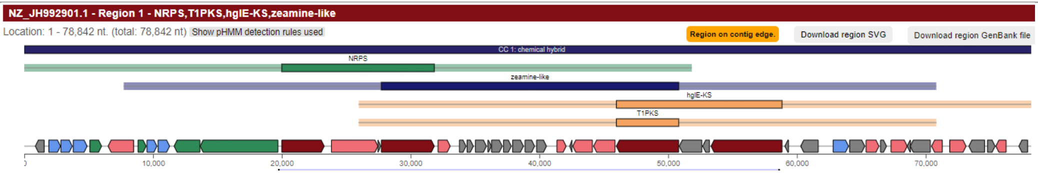


| | | | | |
|---|---|---|---|---|
| KQ976354.1 | *Scytonema hofmannii* PCC 7110 | Nostocales | No | / |

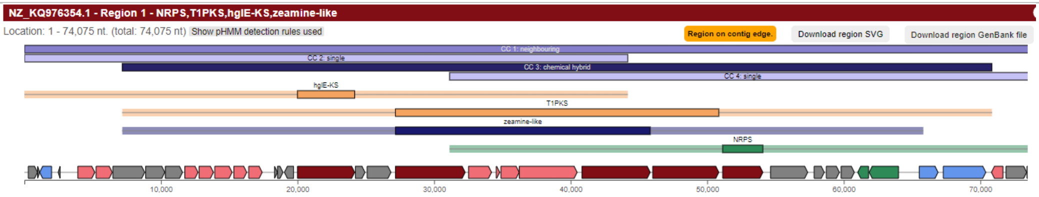


| | | | | |
|---|---|---|---|---|
| VILF01000001.1 | *Dolichospermum flos-aquae* UHCC 0037 | Nostocales | Yes | 85% of genes from heterocyst glycolipid BGC share at least 30% identity to this *pfa* operon |

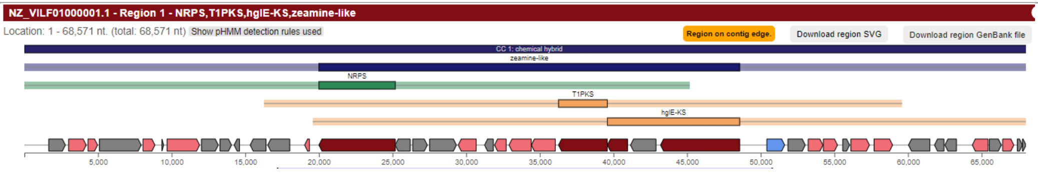


| | | | | |
|---|---|---|---|---|
| CP009962.1 | *Collimonas arenae* Cal35 | Burkholderiales | No | / |

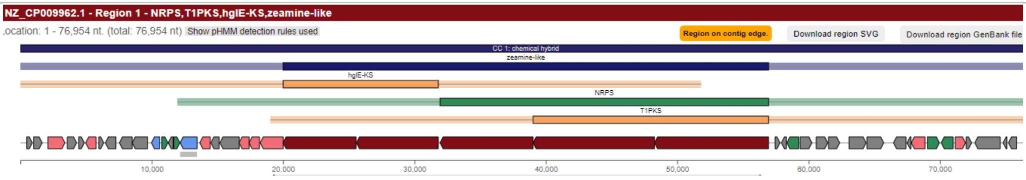


| | | | | |
|---|---|---|---|---|
| CP025429.1 | *Chromobacterium* sp. ATCC 53434 | Burkholderiales | No | / |

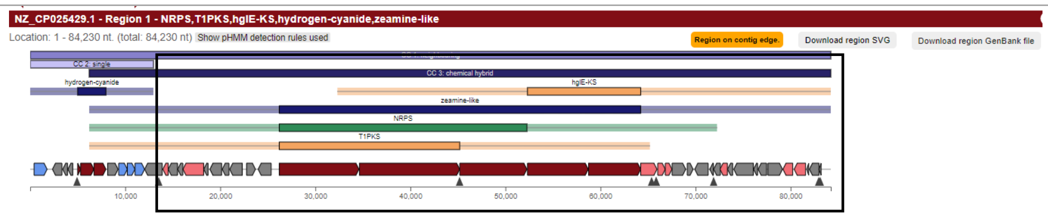


| | | | | |
|---|---|---|---|---|
| CP041743.1 | *Paraburkholderia megapolitana* LMG 23650 | Burkholderiales | No | / |

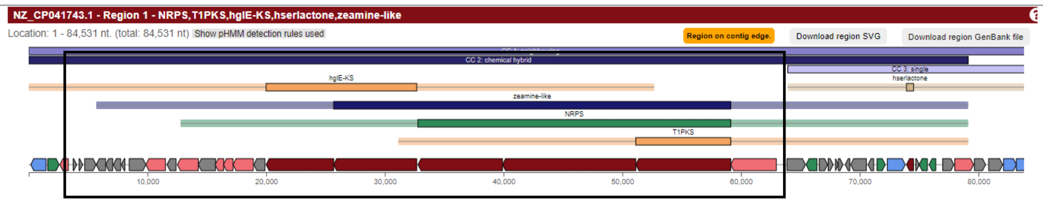


| | | | | |
|---|---|---|---|---|
| KE386747.1 | *Chitinimonas koreensis* DSM 17726 | Burkholderiales | No | / |

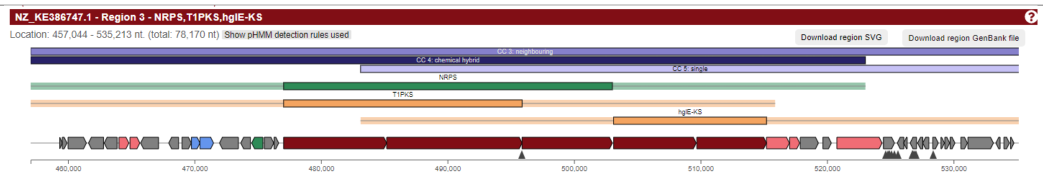


| | | | | |
|---|---|---|---|---|
| AUGX01000034.1 | *Ottowia thiooxydans* DSM 14619 | Burkholderiales | No | / |

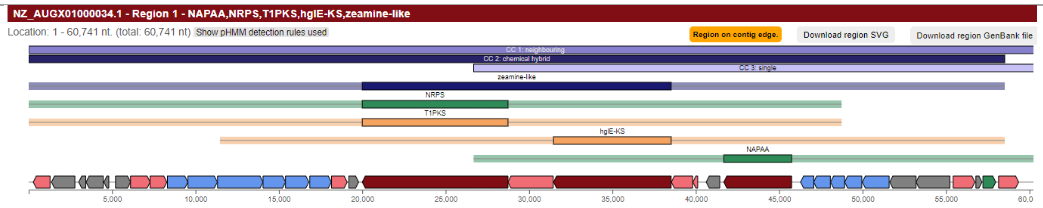


| | | | | |
|---|---|---|---|---|
| FWZX01000003.1 | *Tistlia consotensis* USBA 355 | Rhodospirillales | No | / |

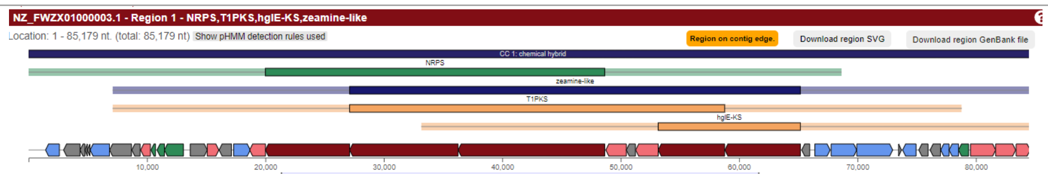


| | | | | |
|---|---|---|---|---|
| SNZH01000009.1 | *Tahibacter aquaticus* DSM 21667 | Lysobacterales | No | / |

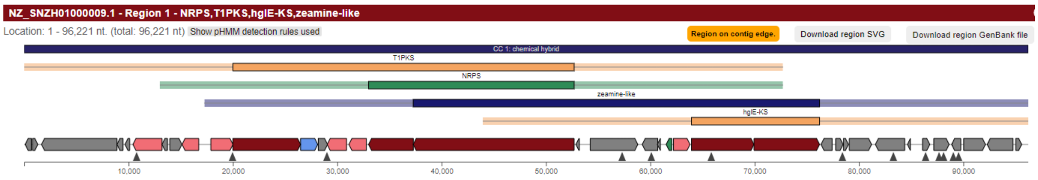


| | | | | |
|---|---|---|---|---|
| CP053590.1 | *Aquimarina* sp. TRL1 | Flavobacteriales | No | / |

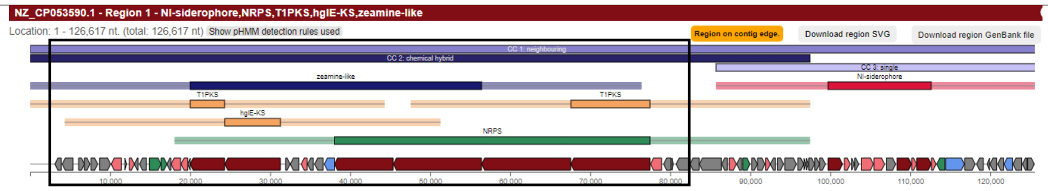


| | | | | |
|---|---|---|---|---|
| CP110012.1 | *Flavobacterium* sp. N502540 | Flavobacteriales | No | / |

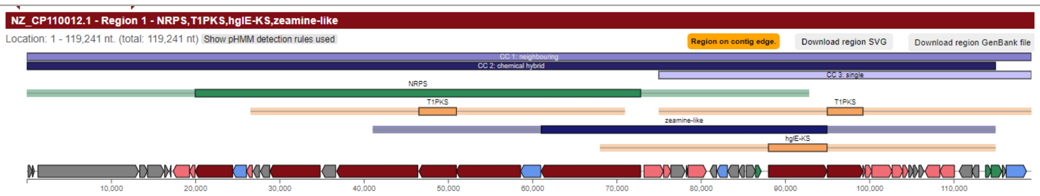


| | | | | |
|---|---|---|---|---|
| CP087134.1 | *Flavobacterium* sp. F-323 | Flavobacteriales | Yes | PfaBC homolog misannotated as T1PKS |

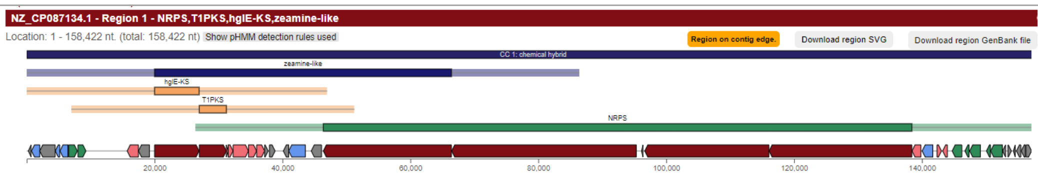


| | | | | |
|---|---|---|---|---|
| KB900627.1 | *Methylosinus* sp. LW4 | Hyphomicrobiales | Yes | 85% of genes from the phenolic lipids BGC share at least 30% identity to this *pfa* operon |

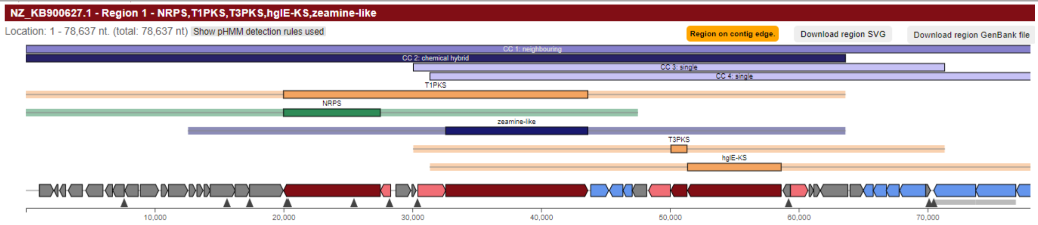


| | | | | |
|---|---|---|---|---|
| SOEJ01000010.1 | *Methylosinus* sp. sav-2 | Hyphomicrobiales | Yes | 85% of genes from the phenolic lipids BGC share at least 30% identity to this *pfa* operon |

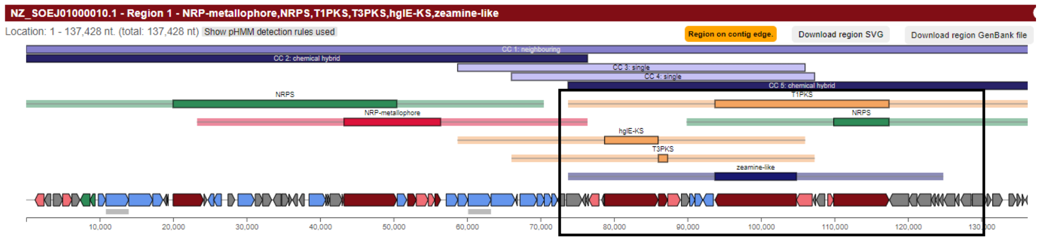


| | | | | |
|---|---|---|---|---|
| AP022333.1 | *Methylosinus* sp. C49 | Hyphomicrobiales | Yes | 85% of genes from the phenolic lipids BGC share at least 30% identity to this *pfa* operon |

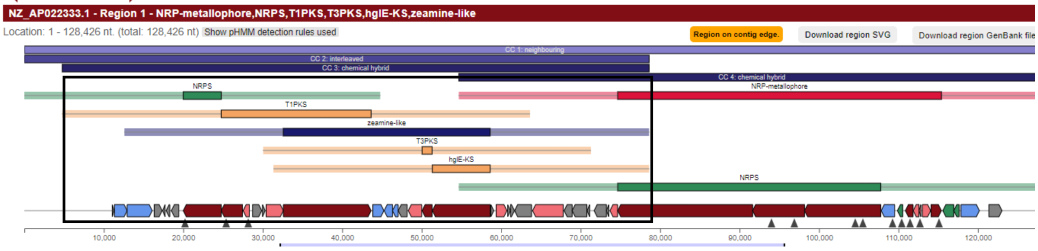


| | | | | |
|---|---|---|---|---|
| AZUO01000004.1 | *Methylosinus* sp. LW3 | Hyphomicrobiales | Yes | 85% of genes from the phenolic lipids BGC share at least 30% identity to this *pfa* operon |

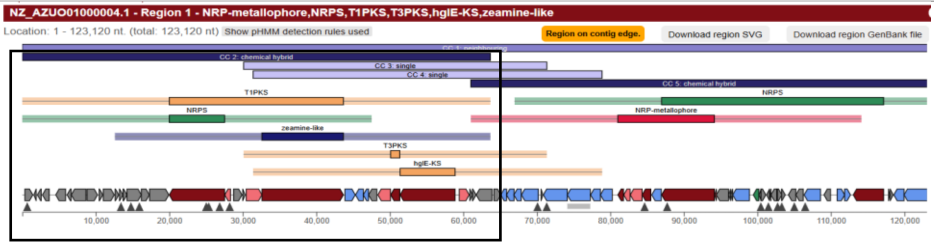


| | | | | |
|---|---|---|---|---|
| JACHHY010000001.1 | *Chitinivorax tropicus* DSM 27165 | Betaproteobacteria | No | / |

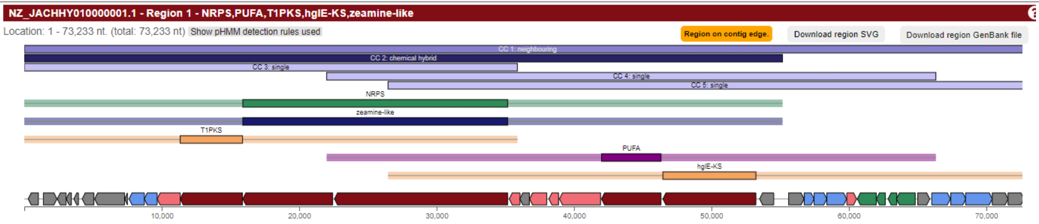


| | | | | |
|---|---|---|---|---|
| CP069161.1 | *Paludibacterium paludis* BCRC 80514 | Neisseriales | No | / |

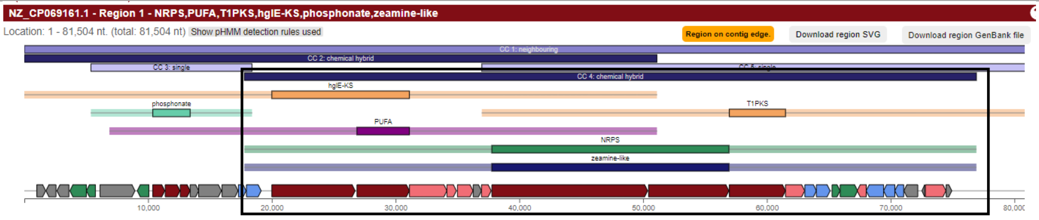


| | | | | |
|---|---|---|---|---|
| PEBU01000004.1 | *Bowmanella denitrificans* JL63 | Alteromonadales | No | / |

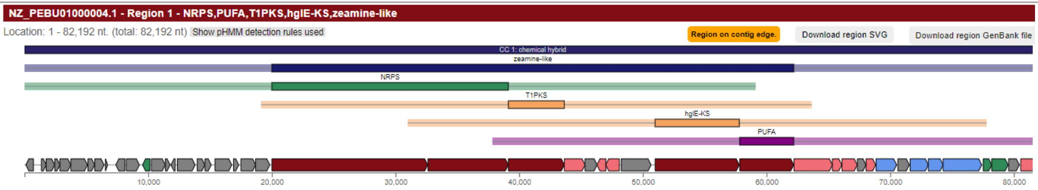


**Gram-positive bacteria**

| | | | | |
|---|---|---|---|---|
| CP001700.1 | *Catenulispora acidiphila* DSM 44928 | Catenulisporales | Yes | PfaBC homolog misannotated as T1PKS. The co-localization and synteny of the three types of biosynthetic genes are not conserved among closely-related strains |

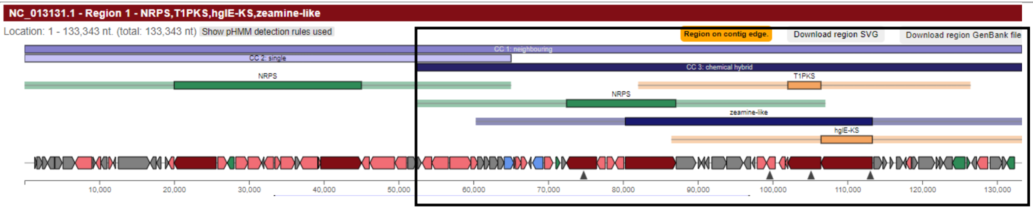


| | | | | |
|---|---|---|---|---|
| CP002047.1 | *Streptomyces bingchenggensis* BCW-1 | Kitasatosporales | Yes | The co-localization and synteny of the three types of biosynthetic genes are not conserved among closely-related strains. The *pfa*-like and the PKS-NRPS genes are separated by considerable distance with many intervening genes and no clear operon-like organization. |

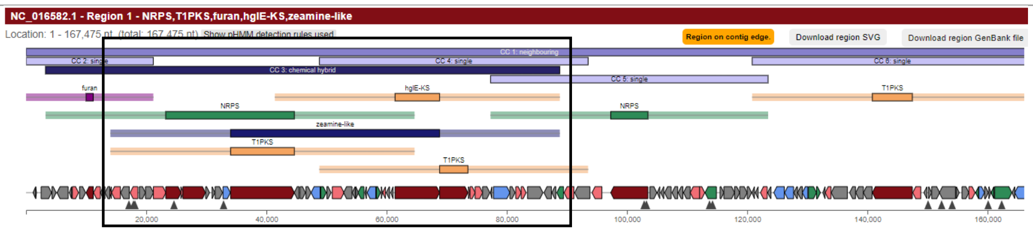


| | | | | |
|---|---|---|---|---|
| BMQK01000010.1 | *Streptomyces ruber* JCM 3131 | Kitasatosporales | Yes | PfaBC homolog misannotated as T1PKS. The co-localization and synteny of the three types of biosynthetic genes are not conserved among closely-related strains. |

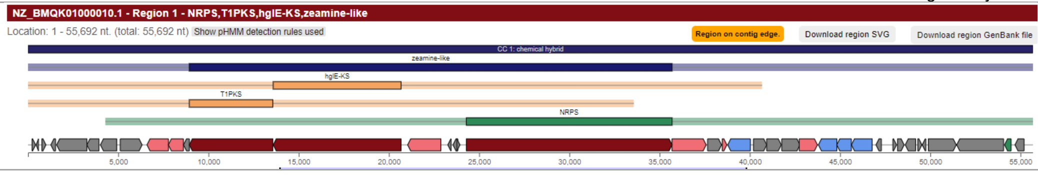


| | | | | |
|---|---|---|---|---|
| BNBJ01000010.1 | *Streptomyces griseus* JCM 4516 | Kitasatosporales | No | / |

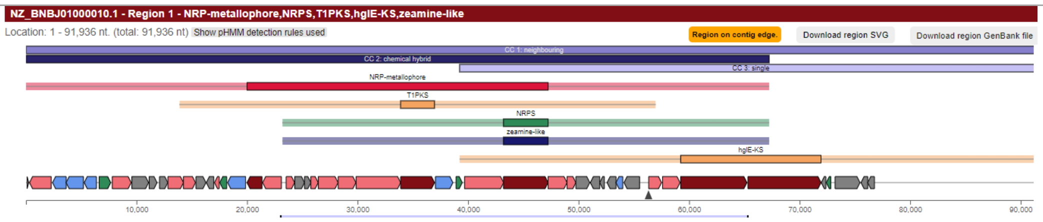


| | | | | |
|---|---|---|---|---|
| CP015849.1 | *Streptomyces* sp. SAT1 | Kitasatosporales | Yes | 100% of genes from the bacillibactin BGC share at least 30% identity to the NRPS operon |

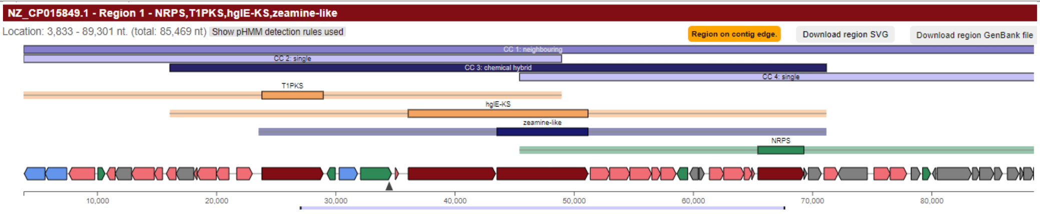


| | | | | |
|---|---|---|---|---|
| CP106840.1 | *Streptomyces* sp. Je 1-4 | Kitasatosporales | Yes | PfaBC homolog misannotated as T1PKS. The co-localization and synteny of the three types of biosynthetic genes are not conserved among closely-related strains. |

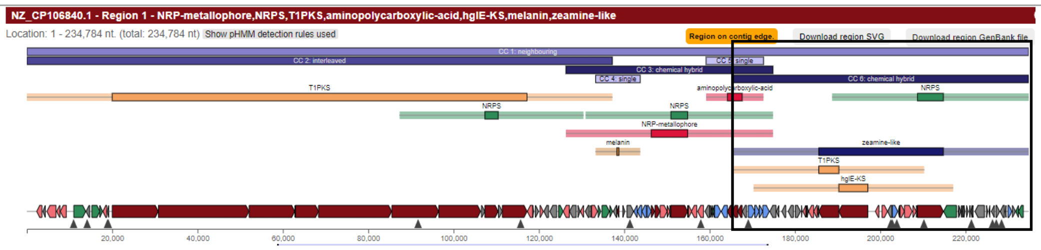


| | | | | |
|---|---|---|---|---|
| JAMFLF010000009.1 | *Streptomyces* sp. 43Y-GA-1 | Kitasatosporales | No | / |

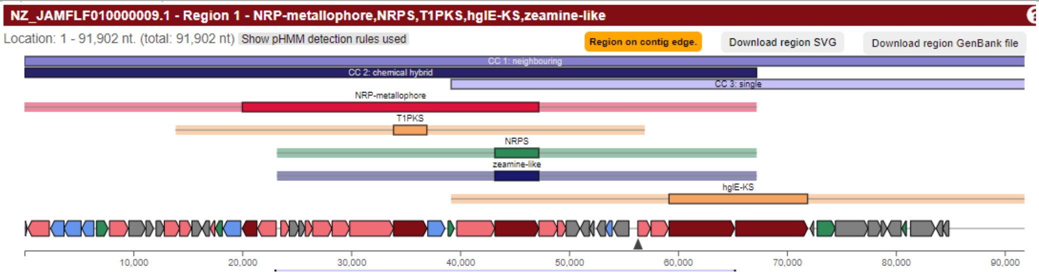


| | | | | |
|---|---|---|---|---|
| KB892157.1 | *Streptomyces* sp. PsTaAH-124 | Kitasatosporales | Yes | 100% of genes from the bacillibactin BGC share at least 30% identity to the NRPS operon. |

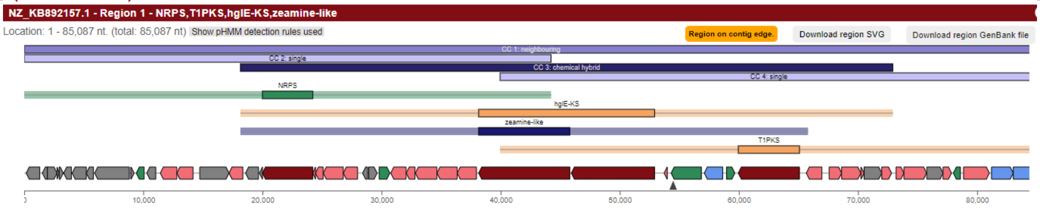


| | | | | |
|---|---|---|---|---|
| KL573545.1 | *Streptomyces baarnensis* NRRL B-2842 | Kitasatosporales | No | / |

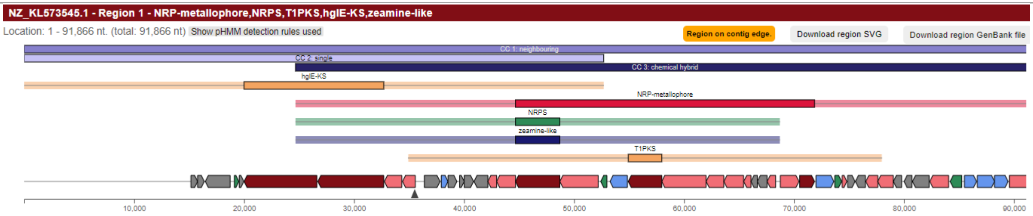

| | | | | |
|---|---|---|---|---|
| ML123034.1 | *Streptomyces* sp. ADI96-02 | Kitasatosporales | Yes | PfaBC homolog misannotated as T1PKS. The co-localization and synteny of the three types of biosynthetic genes are not conserved among closely-related strains. |

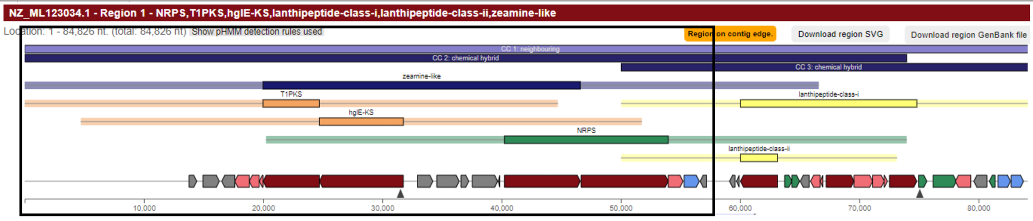


| | | | | |
|---|---|---|---|---|
| CP049782.1 | *Streptomyces* sp. ID38640 | Kitasatosporales | Yes | PfaBC homolog misannotated as T1PKS. The co-localization and synteny of the three types of biosynthetic genes are not conserved among closely-related strains. |

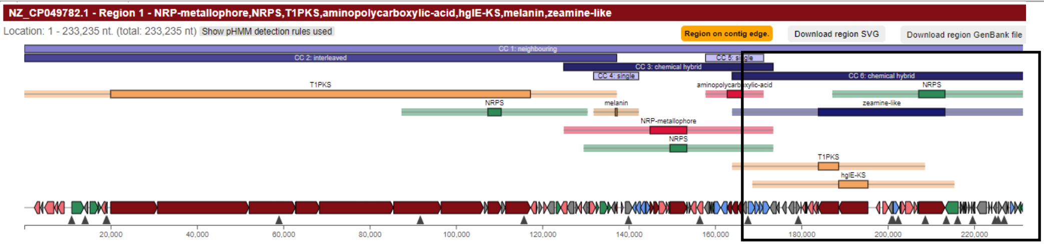


| | | | | |
|---|---|---|---|---|
| CP051486.1 | *Streptomyces pratensis* S10 | Kitasatosporales | Yes | PfaBC homolog misannotated as T1PKS. The co-localization and synteny of the three types of biosynthetic genes are not conserved among closely-related strains. |

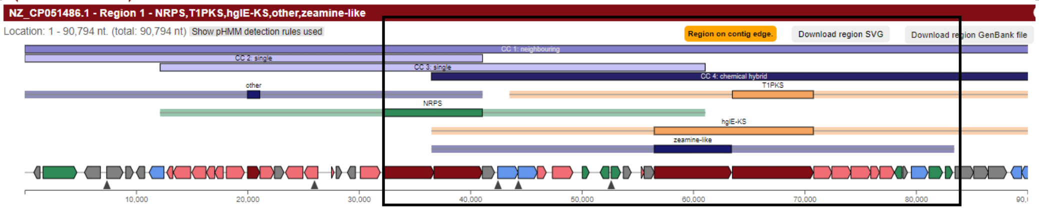


| | | | | |
|---|---|---|---|---|
| CP079114.1 | *Streptomyces* sp. WY228 | Kitasatosporales | No | / |

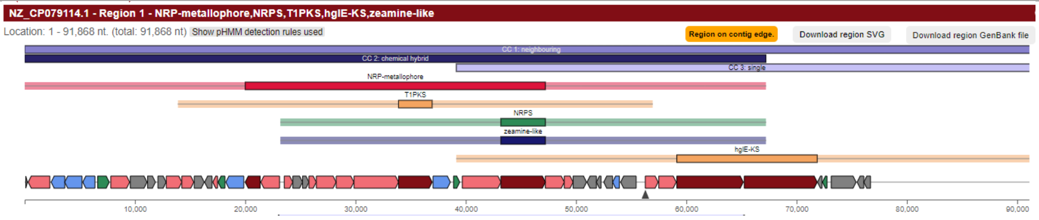


| | | | | |
|---|---|---|---|---|
| CP081496.1 | *Streptomyces* sp. BHT-5-2 | Kitasatosporales | Yes | The co-localization and synteny of the three types of biosynthetic genes are not conserved among closely-related strains. No clear operon-like organization. |

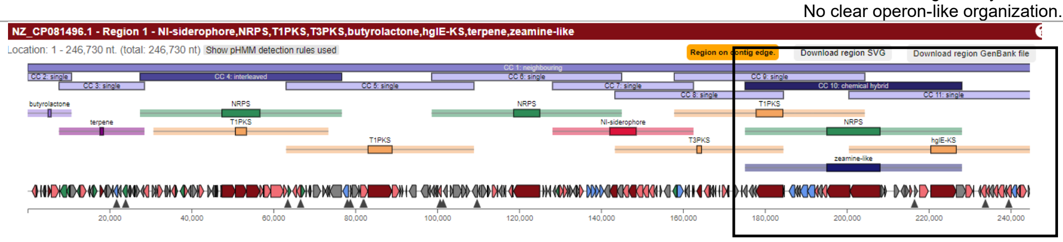


| | | | | |
|---|---|---|---|---|
| FNIX01000003.1 | *Lentzea jiangxiensis* CGMCC 4.6609 | Pseudonocardiales | Yes | The co-localization and synteny of the three types of biosynthetic genes are not conserved among closely-related strains. |

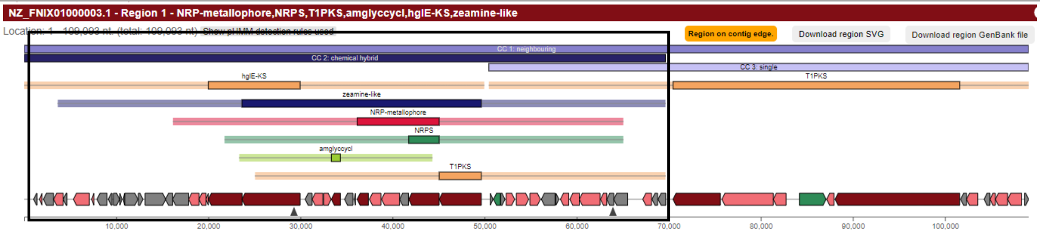


| | | | | |
|---|---|---|---|---|
| JAJCXE010000056.1 | *Lentzea* sp. CC55 | Pseudonocardiales | Yes | The co-localization and synteny of the three types of biosynthetic genes are not conserved among closely-related strains. |

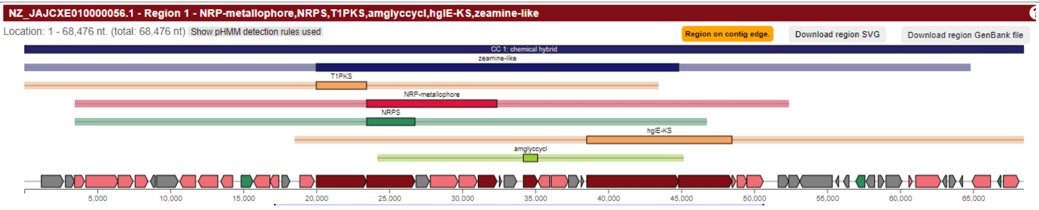


| | | | | |
|---|---|---|---|---|
| CP031142.1 | *Saccharopolyspora pogona* NRRL30141 | Pseudonocardiales | Yes | 82% of genes from the A83543A BGC share at least 30% identity to the T1PKS region |

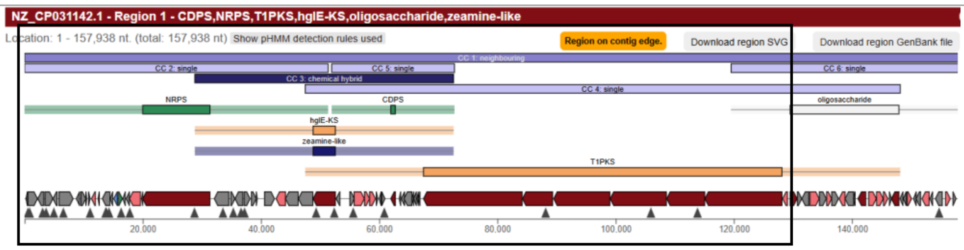


| | | | | |
|---|---|---|---|---|
| JADG01000010.1 | *Actinomadura oligospora* ATCC 43269 | Streptosporangiales | Yes | PfaBC homolog misannotated as T1PKS. |

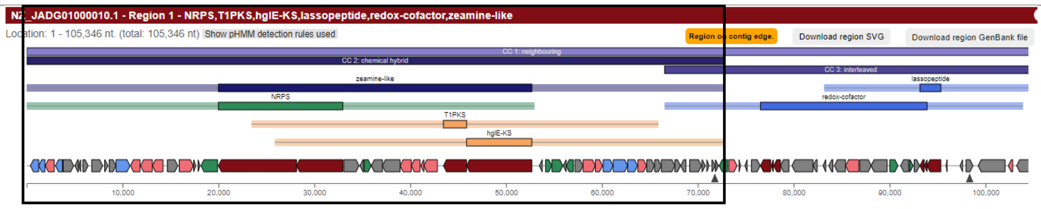


| | | | | |
|---|---|---|---|---|
| MPKW01000003.1 | *Mycolicibacterium* sp. CBMA 234 | Mycobacteriales | No | / |

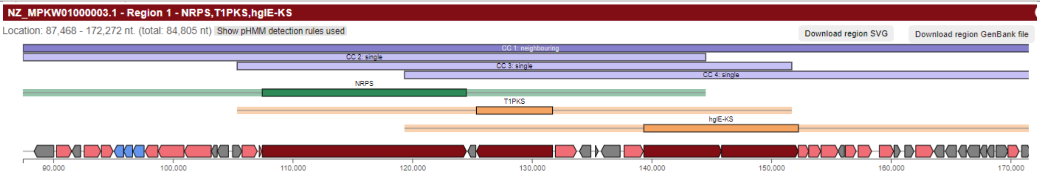


| | | | | |
|---|---|---|---|---|
| VOMB01000005.1 | *Mycobacterium fortunisiensis* TNTM28 | Mycobacteriales | No | / |

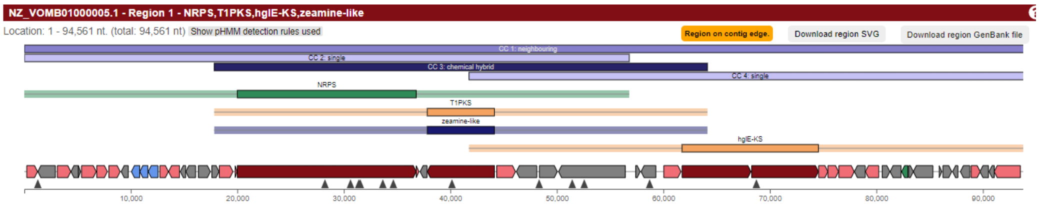


| | | | | |
|---|---|---|---|---|
| OCTY01000002.1 | *Mycobacterium simulans* FB-527 | Mycobacteriales | Yes | PfaBC homolog misannotated as T1PKS. |

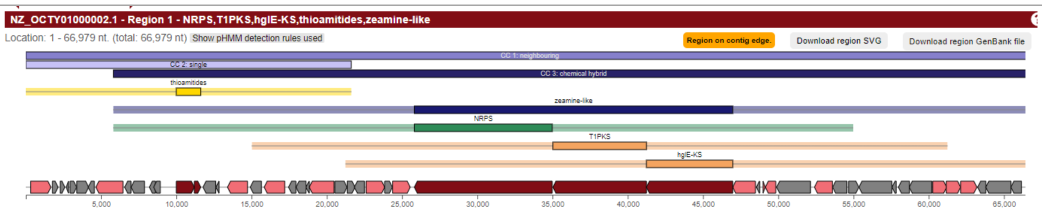
NZ_OCTY01000002.1 - Region 1 - NRPS,T1PKS,hglE-KS,thioamitides,zeamine-like
Location: 1 - 66,979 nt. (total: 66,979 nt)
Show pHMM detection rules used
Region on contig edge.
Download region SVG
Download region GenBank file
CC 1: neighbouring
CC 2: single
CC 3: chemical hybrid
thioamitides
zeamine-like
NRPS
T1PKS
hglE-KS
5,000
10,000
15,000
20,000
25,000
30,000
35,000
40,000
45,000
50,000
55,000
60,000
65,000

**Table S2. Predicted amino acid substrate specificities of the adenylation (A) domains in the zeamine/fabclavine BGCs from *Serratia plymuthica* RVH1, *Xenorhabdus hominickii* ANU1, *Bowmanella denitrificans* JL63, *Paludibacterium paludis* BCRC 80514 and *Chitinivorax tropicus* DSM 27165.** Overview of the predicted substrate binding residues in each A domain, along with specificity predictions obtained using antiSMASH and PARAS (Predictive Algorithm for Resolving Adenylation domain Selectivity).[44] The second amino acid incorporated (underlined) is distinct in zeamine and fabclavine compounds, and therefore, is used as a parameter to predict which out of the two metabolites *B. denitrificans, P. paludis* and *C. tropicus* are expected to produce.[63,64]

| | | |
|---|---|---|
| ***Serratia plymuthica* RVH1 (zeamines)** | | |
| **Zmn16_A1** | Predicted binding residues | DPRHLALLAK |
| | AntiSMASH prediction | Asp |
| | PARAS prediction | Asp (0.637) |
| | Actual amino acid incorporated | Asp |
| **Zmn16_A2** | Predicted binding residues | DTWTIASVSK |
| | AntiSMASH prediction | His |
| | PARAS prediction | His (0.674) |
| | Actual amino acid incorporated | His |
| **Zmn16_A3** | Predicted binding residues | DATKVGEVGK |
| | AntiSMASH prediction | Asn |
| | PARAS prediction | Asn (0.968) |
| | Actual amino acid incorporated | Asn |
| **Zmn16_A4** | Predicted binding residues | DATKVGEVGK |
| | AntiSMASH prediction | Asn |
| | PARAS prediction | Asn (0.960) |
| | Actual amino acids incorporated | Asn |
| **Zmn17_A5** | Predicted binding residues | DFWNIGMVHK |
| | AntiSMASH prediction | Thr |
| | PARAS prediction | Thr (0.939) |
| | Actual amino acids incorporated | Thr |
| **Zmn17_A6** | Predicted binding residues | DALFIGGTFK |
| | AntiSMASH prediction | Val |
| | PARAS prediction | Val (0.782) |
| | Actual amino acids incorporated | Val |
| ***Xenorhabdus hominickii* ANU1 (fabclavines)** | | |
| **Xbud_02640_A1** | Predicted binding residues | DPRHLSLLAK |
| | AntiSMASH prediction | X |
| | PARAS prediction | Asp (0.249) |
| | Actual amino acid incorporated | Asp |
| **Xbud_02640_A2** | Predicted binding residues | DTWTIASVGK |
| | AntiSMASH prediction | Phe |
| | PARAS prediction | Phe (0.636) |
| | Actual amino acid incorporated | Phe (Other variants might include His or Ala) |
| **Xbud_02640_A3** | Predicted binding residues | DATKVGEVGK |
| | AntiSMASH prediction | Asn |
| | PARAS prediction | Asn (0.813) |
| | Actual amino acid incorporated | |
| **Xbud_02640_A4** | Predicted binding residues | DATKVGEVGK |
| | AntiSMASH prediction | Asn |
| | PARAS prediction | Asn (0.965) |
| | Actual amino acids incorporated | |
| **Xbud_02641_A5** | Predicted binding residues | DFWNIGMVHK |
| | AntiSMASH prediction | Thr |
| | PARAS prediction | Thr (1.000) |
| | Actual amino acids incorporated | |
| **Xbud_02641_A6** | Predicted binding residues | DALFIGGTFK |
| | AntiSMASH prediction | Val |
| | PARAS prediction | Val (0.476) |
| | Actual amino acids incorporated | Val (Other variants might include Pro or Thr) |
| ***Bowmanella denitrificans* JL63** | | |
| **CSR04_RS09940_A1** | Predicted binding residues | DPRHLALLAK |
| | AntiSMASH prediction | Asp |
| | PARAS prediction | Asp (0.449) |
| **CSR04_RS09940_A2** | Predicted binding residues | DTWTIASVSK |

| | | |
|---|---|---|
| | AntiSMASH prediction | His |
| | PARAS prediction | His (0.417) |
| **CSR04_RS09940_A3** | Predicted binding residues | DATKVGEVGK |
| | AntiSMASH prediction | Asn |
| | PARAS prediction | Asn (0.672) |
| **CSR04_RS09940_A4** | Predicted binding residues | DATKVGEVGK |
| | AntiSMASH prediction | Asn |
| | PARAS prediction | Asn (0.870) |
| **CSR04_RS09945_A5** | Predicted binding residues | DFWNVGMVHK |
| | AntiSMASH prediction | Thr |
| | PARAS prediction | Thr (1.000) |
| **CSR04_RS09945_A6** | Predicted binding residues | DAFFLGATFK |
| | AntiSMASH prediction | X |
| | PARAS prediction | Val (0.374) |
| ***Paludibacterium paludis* BCRC 80514** | | |
| **JNO50_RS03090_A1** | Predicted binding residues | DPRHAALLAK |
| | AntiSMASH prediction | X |
| | PARAS prediction | Asp (0.247) |
| **JNO50_RS03090_A2** | Predicted binding residues | DSWTIASVSK |
| | AntiSMASH prediction | X |
| | PARAS prediction | His (0.379) |
| **JNO50_RS03090_A3** | Predicted binding residues | DATKVGEVGK |
| | AntiSMASH prediction | Asn |
| | PARAS prediction | Asn (0.941) |
| **JNO50_RS03090_A4** | Predicted binding residues | DATKVGEVGK |
| | AntiSMASH prediction | Asn |
| | PARAS prediction | Asn (0.854) |
| **JNO50_RS03095_A5** | Predicted binding residues | DFWNIGMVHK |
| | AntiSMASH prediction | Thr |
| | PARAS prediction | Thr (0.995) |
| **JNO50_RS03095_A6** | Predicted binding residues | DAMFIGGTFK |
| | AntiSMASH prediction | Val |
| | PARAS prediction | Val (0.577) |
| ***Chitinivorax tropicus* DSM 27165** | | |
| **HNQ59_RS00070_A1** | Predicted binding residues | DPRHAALLAK |
| | AntiSMASH prediction | X |
| | PARAS prediction | Asp (0.262) |
| **HNQ59_RS00070_A2** | Predicted binding residues | DTWTIASVSK |
| | AntiSMASH prediction | His |
| | PARAS prediction | His (0.448) |
| **HNQ59_RS00070_A3** | Predicted binding residues | DATKVGEVGK |
| | AntiSMASH prediction | Asn |
| | PARAS prediction | Asn (0.977) |
| **HNQ59_RS00070_A4** | Predicted binding residues | DATKVGEVGK |
| | AntiSMASH prediction | Asn |
| | PARAS prediction | Asn (0.854) |
| **HNQ59_RS00070_A5** | Predicted binding residues | DFWNVGMVHK |
| | AntiSMASH prediction | Thr |
| | PARAS prediction | Thr (1.000) |
| **HNQ59_RS00070_A6** | Predicted binding residues | DAFFFGGTFK |
| | AntiSMASH prediction | Val |
| | PARAS prediction | Val (0.533) |

**Table S3. Putative functions and closest homologs of the genes within and surrounding the chitinimine BGC.** Overview of genes, the putative function and predicted size of the proteins they encode, alongside the most similar known protein (protein ID, species and identity percentage), as determined by BlastP.

| Gene identifier | Putative function of gene product | Size (aa) | Top BlastP Hit | % Identity |
|---|---|---|---|---|
| *F559_RS0116810* | Type II toxin-antitoxin system VapB family antitoxin | 65 | MDO9386543.1 (*Thiobacillus* sp.) | 77.78 |
| *F559_RS0116815* | Type II toxin-antitoxin system VapC family toxin | 124 | WP_099407028.1 (*Chitinimonas* sp. BJB300) | 67.74 |
| *F559_RS26255* | Exonuclease domain-containing protein | 482 | GLR12994.1 (*Chitinimonas prasina*) | 66.95 |
| *F559_RS26260* | Lytic murein transglycosylase B | 396 | WP_331985645.1 (*Chitinimonas* sp.) | 73.80 |
| *F559_RS0116830* | Heat-inducible transcriptional repressor | 338 | WP_290334435.1 (*Chitinimonas viridis*) | 89.35 |
| *F559_RS0116835* | Transporter substrate-binding domain-containing protein | 259 | WP_290334434.1 (*Chitinimonas viridis*) | 79.10 |
| *F559_RS0116840* | Transporter substrate-binding domain-containing protein | 259 | WP_099405814.1 (*Chitinimonas sp. BJB300*) | 75.68 |
| *F559_RS26265* | SGNH/GDSL hydrolase family protein (carbohydrate modifying enzyme) | 471 | WP_335709779.1 (*Chitinimonas* sp. JJ19) | 89 |
| *F559_RS26270* | TOBE domain-containing protein (ATP-binding) | 259 | WP_331989049.1 (*Chitinimonas* sp.) | 67.57 |
| *F559_RS0116855* | Molybdate ABC transporter substrate-binding protein | 246 | WP_367788558.1 (Chi*tinivorax* sp. PXF-14) | 75.83 |
| *F559_RS0116860* | Molybdate ABC transporter permease subunit | 225 | WP_367788557.1 (Chitinivorax sp. PXF-14) | 77.52 |
| *F559_RS0116865* | Molybdenum ABC transporter ATP-binding protein | 357 | WP_331989043.1 (*Chitinimonas sp.*) | 69.86 |
| *F559_RS0116870* | AMP nucleosidase | 497 | WP_367788685.1 (*Chitinivorax* sp. PXF-14) | 82.49 |
| *F559_RS0116875* | ABC transporter substrate-binding protein | 254 | WP_035055253.1 (*Andreprevotia chitinilytica*) | 69.88 |
| *F559_RS0116880* | LysR family transcriptional regulator | 295 | WP_128683155.1 (*Pseudomonas aeruginosa*) | 70.34 |
| *F559_RS0116885* | CTP synthase | 251 | WP_199693560.1 (*Sorangium cellulosum*) | 66.38 |
| *F559_RS0116890* | Antibiotic biosynthesis monooxygenase | 121 | WP_263150353.1 (*Pseudomonas tohonis*) | 82.37 |
| *F559_RS26275 = chtnA* | Hybrid NRPS-type I PKS | 2715 | WP_099405553.1 (*Chitinimonas* sp. BJB300) | 65.99 |
| *F559_RS28020 = chtnB* | Hybrid NRPS-type I PKS | 3568 | WP_099405554.1 (*Chitinimonas* sp. BJB300) | 64.02 |
| *F559_RS0116905 = chtnC* | NRPS | 2379 | WP_099405555.1 (*Chitinimonas* sp. BJB300) | 62.48 |
| *F559_RS0116910 = chtnD* | Type I PKS | 2176 | WP_158228936.1 (*Chitinimonas* sp. BJB300) | 60.38 |
| *F559_RS0116915 = chtnE* | β-ketoacyl synthase N-terminal-like domain-containing protein | 1837 | WP_099405557.1 (*Chitinimonas* sp. BJB300) | 76.58 |
| *F559_RS26285 = chtnF* | PfaD family polyunsaturated fatty acid/polyketide biosynthesis protein | 602 | WP_099405558.1 (*Chitinimonas* sp. BJB300) | 72.67 |
| *F559_RS0116930 = chtnG* | Thioesterase II family protein | 265 | WP_325666666.1 (*Collimonas* sp.) | 63.95 |
| *F559_RS0116935 = chtnH* | Polysaccharide pyruvyl transferase family protein | 477 | WP_047243952.1 (*Chromobacterium subtsugae*) | 68.55 |
| *F559_RS26290* | Hypothetical protein | 220 | MBV8657729.1 (*Burkholderiales* bacterium) | 62.11 |
| *F559_RS26295* | EAL domain-containing protein, partial | 1190 | WP_331987312.1 (*Chitinimonas* sp.) | 72.51 |
| *F559_RS29640* | Hypothetical protein | 291 | WP_296888885.1 (*Thiobacillus* sp.) | 58.94 |
| *F559_RS0116950* | HEPN domain-containing protein | 172 | HST20945.1 (*Blastocatellia bacterium)* | 45.78 |
| *F559_RS0116955* | Nucleotidyltransferase domain-containing protein | 93 | MCB2263725.1 (Candidatus Thiosymbion ectosymbiont of *Robbea hypermnestra*) | 53.76 |
| *F559_RS29645* | Hypothetical protein | 170 | WP_084091439.1 (*Andreprevotia lacus*) | 59.17 |
| *F559_RS0116960* | Hypothetical protein | 134 | - | - |
| *F559_RS26300* | GNAT family N-acetyltransferase | 160 | OWQ46416.1 (*Roseateles noduli*) | 66.67 |
| *F559_RS0116970* | Hypothetical protein | 145 | WP_285234984.1 (*Paucibacter sediminis*) | 67.20 |
| *F559_RS29650* | AbrB/MazE/SpoVT family DNA-binding domain-containing protein | 91 | WP_251972849.1 (*Sphaerotilus microaerophilus*) | 39.78 |
| *F559_RS0116980* | Type II toxin-antitoxin system VapC family toxin | 128 | MDX2043469.1 (*Acidobacteriota* bacterium) | 46.51 |
| *F559_RS0116985* | VOC (vicinal oxygen chelate) family protein Glyoxalase | 125 | HTE15913.1 (*Burkholderiales* bacterium) | 74.40 |
| *F559_RS0116990* | DUF421 domain-containing protein | 171 | HEX5128416.1 (*Usitatibacter* sp.) | 57.89 |
| *F559_RS0116995* | AsmA family protein | 671 | WP_378161007.1 (*Chitinimonas lacunae*) | 54.02 |
| *F559_RS30135* | Hypothetical protein | 111 | - | - |
| *F559_RS30140* | Glycine zipper 2TM domain-containing protein | 145 | WP_300111117.1 (*Rhodoferax* sp.) | 40.58 |
| *F559_RS26310* | Hypothetical protein | 91 | WP_378161013.1 (*Chitinimonas lacunae*) | 62.34 |

**Table S4**. **Overview of NMR signals observed for chitinimine I isolated from *C. koreensis* DSM 17726 (DMSO-d6, $^{1}H$ 600 MHz, $^{13}C$ 151 MHz).** Multiplicity was reported with the following notation, or a combination thereof: s = singlet, d = doublet, t = triplet, q = quartet, quin = quintet, sex = sextet, sept = septet, m = multiplet and br. = broad. nd = not determinable

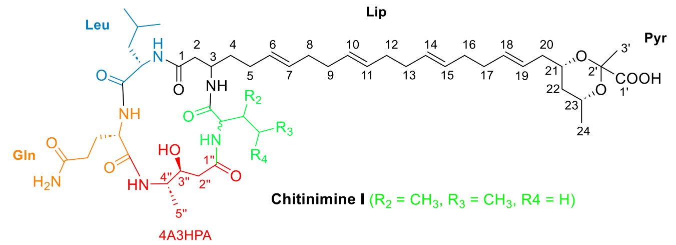


| Position | $^{1}H$ (ppm) | $J_{H\text{-}H}$ (Hz) | $^{13}C$ (ppm) |
|---|---|---|---|
| **(*allo*-)Ile** | | | |
| C=O | - | - | 170.5 |
| α-CH | 4.01 | dd, 6.9, 4.6 | 57.9 |
| β-CH | 1.78 | dt, 4.6, 6.6 | 35.0 |
| β-$CH_3$ | 0.86 | d, 6.6 | 14.8 |
| γ-$CH_2$ | 1.32,1.16 | m | 25.6 |
| δ-$CH_3$ | 0.82 | t, 6.6 | 11.4 |
| NH | 8.11 | d, 6.9 | - |
| **4-amino-3-hydroxypentanoic acid** | | | |
| 1''-CO | - | - | 171.8 |
| 2''-$CH_2$ | 2.50, 2.08 | dd, 10, 6 | 39.9 |
| 3''-CH | 3.92 | m, 7.1, 6, 6 | 69.6 |
| 3''-OH | 4.83 | d, 6 | - |
| 4''-CH | 3.41 | dq, 7.1, 6.2 | 49.4 |
| 4''-NH | 7.75 | d, 6.2, br. | - |
| 5''-$CH_3$ | 1.01 | d, 6.2 | 14.8 |
| **Gln** | | | |
| C=O | - | - | 171.1 |
| α-CH | 4.03 | dt, 12, 7.6 | 54.6 |
| β-$CH_2$ | 1.84 | m | 27.6 |
| γ-$CH_2$ | 2.15, 1.95 | dt, 13.8, 6.3 | 32.0 |
| δ-CO | - | - | 173.7 |
| δ-$NH_2$ | 7.67, 6.63 | - | - |
| NH | 8.58 | d, 12 | - |
| **Leu** | | | |
| C=O | - | - | 172.2 |
| α-CH | 4.01 | dq, br. | 52.8 |
| β-$CH_2$ | 1.59, 1.47 | m | 39.4 |
| γ-CH | 1.73 | m | 24.2 |
| δ1-$CH_3$ | 0.80 | d, 6.6 | 20.4 |
| δ2-$CH_3$ | 0.90 | d, 6.6 | 23.0 |
| NH | 8.89 | d, br. | - |
| **Specialized lipid** | | | |
| 1-CO | - | | 172.9 |
| 2-$CH_2$ | 2.50, 2.42 | | 36.4 |
| 3-CH | 3.80 | | 45.7 |
| 3-NH | 8.17 | d, 7.11 | - |
| 4-$CH_2$ | 1.44 | | 32.7 |
| 5-$CH_2$ | 1.92 | | 29.0 |
| 6-CH | 5.36 | | 129.7-129.5 |
| 7-CH | 5.36 | | 129.7-129.5 |
| 8-$CH_2$ | 2.01 | | 31.8-31.2 |
| 9-$CH_2$ | 2.01 | | 31.8-31.2 |
| 10-CH | 5.36 | | 129.7-129.5 |
| 11-CH | 5.36 | | 129.7-129.5 |
| 12-$CH_2$ | 2.01 | | 31.8-31.2 |
| 13-$CH_2$ | 2.01 | | 31.8-31.2 |

| | | | |
|---|---|---|---|
| 14-CH | 5.36 | | 129.7-129.5 |
| 15-CH | 5.36 | | 129.7-129.5 |
| 16-$CH_2$ | 2.01 | | 31.8-31.2 |
| 17-$CH_2$ | 2.01 | | 31.8-31.2 |
| 18-CH | 5.39 | | 126.1 |
| 19-CH | 5.42 | | 131.6 |
| 20-$CH_2$ | 2.17, 1.98 | | 39.1 |
| 21-CH | 3.82 | m, 17.43 with Hax and 6 with Heq, 14 | 68.2 |
| 21-OH | nd | | - |
| 22-$CH_2$ | 1.46 ($H_{eq}$), 1.00 ($H_{ax}$) | m | 37.9 |
| 23-CH | 3.95 | Dq,17.9 with Hax, 6.5 with 24 and 6 with Heq | 64.4 |
| 24-$CH_3$ | 1.06 | d, 6.5 | 22.0 |
| **Pyruvate** | | | |
| 1'-CO | - | | 172.8 |
| 2'-qC | - | | 97.8 |
| 3'-$CH_3$ | 1.44 | | 17.4 |

**Table S5. Overview of NMR signals observed for chitinimine II isolated from *C. koreensis* DSM 17726 (DMSO-d6, $^1$H 600 MHz, $^{13}$C 151 MHz).** Multiplicity was reported with the following notation, or a combination thereof: s = singlet, d = doublet, t = triplet, q = quartet, quin = quintet, sex = sextet, sept = septet, m = multiplet and br. = broad. nd = not determinable.

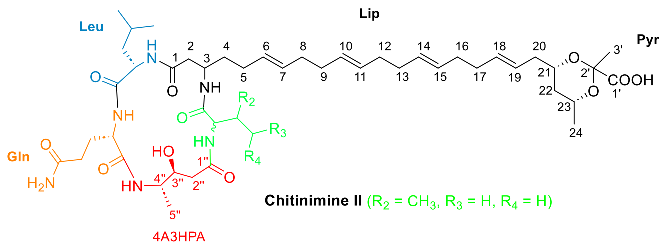


| Position | $^1$H (ppm) | $J_{H\text{-}H}$ (Hz) | $^{13}$C (ppm) |
|---|---|---|---|
| **Val** | | | |
| C=O | - | - | nd |
| α-CH | 3.76 | dd, 7.2, 6.8 | 60.7 |
| β-CH | 1.94 | dsept, 6.8, 7 | 28.7 |
| γ-$CH_3$ | 0.90, 0.88 | d, 7 | 18.6, 19.3 |
| NH | 8.21 | d, 7.2 | - |
| **4-amino-3-hydroxypentanoic acid** | | | |
| 1''-CO | - | - | nd |
| 2''-$CH_2$ | 2.50, 2.03 | dd, 9.2, 6 on 2.03, and d 9.2 on 2.50 | 40.0 |
| 3''-CH | 3.92 | m | 69.6 |
| 3"-OH | 4.03 | d, br. | - |
| 4''-CH | 3.41 | m | 49.4 |
| 4"-NH | 7.93 | d, 8.5, br. | - |
| 5''-$CH_3$ | 1.01 | d, 6.7 | 15.8 |
| **Gln** | | | |
| C=O | - | - | nd |
| α-CH | 4.03 | dd, 12.2, 5.7 | 54.6 |
| β-$CH_2$ | 1.85 | m | 27.9 |
| γ-$CH_2$ | 2.18, 1.93 | m | 32.0 |
| δ-CO | - | - | nd |
| δ-$NH_2$ | 7.81, 6.60 | - | - |
| NH | 8.88 | d, 12.2, br. | - |
| **Leu** | | | |
| C=O | - | - | nd |
| α-CH | 4.03 | dq, 11.8, 6.7 | 52.9 |
| β-$CH_2$ | 1.63, 1.46 | m | 39.5 |

| | | | |
|---|---|---|---|
| γ-CH | 1.73 | m | 24.3 |
| δ1-$CH_3$ | 0.80 | d, 7 | 20.4 |
| δ2-$CH_3$ | 0.90 | d, 7 | 23.0 |
| NH | 9.20 | d, 11.8, br. | - |
| **Specialized lipid** | | | |
| 1-CO | - | - | nd |
| 2-$CH_2$ | 2.52, 2.44 | | 36.2 |
| 3-CH | 3.78 | | 45.5 |
| 3-NH | 8.09 | d, 8.6 | - |
| 4-$CH_2$ | 1.44 | | 32.7 |
| 5-$CH_2$ | 1.92 | | 29.0 |
| 6-CH | 5.36 | | 129.7-129.5 |
| 7-CH | 5.36 | | 129.7-129.5 |
| 8-$CH_2$ | 2.01 | | 31.8-31.2 |
| 9-$CH_2$ | 2.01 | | 31.8-31.2 |
| 10-CH | 5.36 | | 129.7-129.5 |
| 11-CH | 5.36 | | 129.7-129.5 |
| 12-$CH_2$ | 2.01 | | 31.8-31.2 |
| 13-$CH_2$ | 2.01 | | 31.8-31.2 |
| 14-CH | 5.36 | | 129.7-129.5 |
| 15-CH | 5.36 | | 129.7-129.5 |
| 16-$CH_2$ | 2.01 | | 31.8-31.2 |
| 17-$CH_2$ | 2.01 | | 31.8-31.2 |
| 18-CH | 5.39 | | 126.1 |
| 19-CH | 5.42 | | 131.6 |
| 20-$CH_2$ | 2.17, 1.98 | | 39.1 |
| 21-CH | 3.82 | m, 17.43 with Hax and 6 with Heq, 14 | 68.2 |
| 21-OH | nd | | - |
| 22-$CH_2$ | 1.46 ($H_{eq}$), 1.00 ($H_{ax}$) | m | 37.9 |
| 23-CH | 3.95 | Dq,17.9 with Hax, 6.5 with 24 and 6 with Heq | 64.4 |
| 24-$CH_3$ | 1.06 | d, 6.5 | 22.0 |
| **Pyruvate** | | | |
| 1'-CO | - | | 172.3 |
| 2'-qC | - | | 97.8 |
| 3'-$CH_3$ | 1.43 | | 17.5 |

**Table S6. Overview of NMR signals observed for chitinimine III isolated from *C. koreensis* DSM 17726 (DMSO-d6, $^1H$ 600 MHz, $^{13}C$ 151 MHz).** Multiplicity was reported with the following notation, or a combination thereof: s = singlet, d = doublet, t = triplet, q = quartet, quin = quintet, sex = sextet, sept = septet, m = multiplet and br. = broad. nd = not determinable.

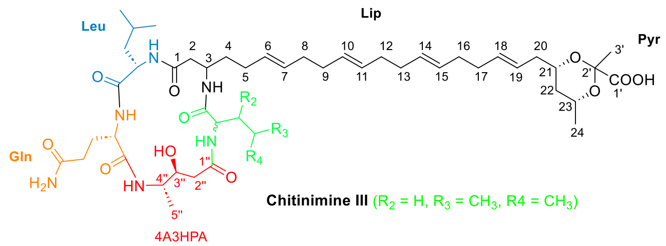


| Position | $^1H$ (ppm) | $J_{H\text{-}H}$ (Hz) | $^{13}C$ (ppm) |
|---|---|---|---|
| **Leu** | | | |
| C=O | - | - | nd |
| α-CH | 3.96 | dq 9.7, 6.2 | 53.2 |
| β-$CH_2$ | 1.44, 1.39 | m | 39.8 |
| γ-CH | 1.66 | m | 24.2 |
| δ1-$CH_3$ | 0.82 | d, 6.6 | 21.2 |

| | | | |
|---|---|---|---|
| δ2-$CH_3$ | 0.88 | d, 6.6 | 22.8 |
| NH | 8.34 | d, 6.2 | - |
| **4-amino-3-hydroxypentanoic acid** | | | |
| 1''-CO | - | | 171.5 |
| 2''-$CH_2$ | 2.45, 2.00 | dd, 10, 6 | 40.0 |
| 3''-CH | 3.90 | m, 7.1, 6, 6 | 69.6 |
| 3''-OH | 4.82 | d, 6 | - |
| 4''-CH | 3.41 | Dq, 7.1, 6.2, br. | 49.4 |
| 4''-NH | 7.74 | d, br. | - |
| 5''-$CH_3$ | 1.01 | d, 6.2 | 14.8 |
| **Gln** | | | |
| C=O | - | - | 171.1 |
| α-CH | 4.03 | dt, 12, 7.6 | 54.6 |
| β-$CH_2$ | 1.84 | m | 27.6 |
| γ-$CH_2$ | 2.15, 1.95 | dt, 13.8, 6.3 | 32.0 |
| δ-CO | - | - | 173.7 |
| δ-$NH_2$ | 7.67, 6.63 | - | - |
| NH | 8.58 | d, 12 | - |
| **Leu** | | | |
| C=O | - | - | 172.2 |
| α-CH | 4.01 | dq, 11.8, 6.7 | 52.8 |
| β-$CH_2$ | 1.59, 1.47 | m | 39.4 |
| γ-CH | 1.73 | m | 24.2 |
| δ1-$CH_3$ | 0.80 | d, 7 | 20.4 |
| δ2-$CH_3$ | 0.90 | d, 7 | 23.0 |
| NH | 8.89 | d, 11.8, br. | - |
| **Specialized lipid** | | | |
| 1-CO | - | | 172.9 |
| 2-$CH_2$ | 2.50, 2.40 | | 36.6 |
| 3-CH | 3.79 | | 45.5 |
| 3-NH | 8.16 | d, 8.8 | - |
| 4-$CH_2$ | 1.44 | | 32.8 |
| 5-$CH_2$ | 1.92 | | 29.0 |
| 6-CH | 5.36 | | 129.7-129.5 |
| 7-CH | 5.36 | | 129.7-129.5 |
| 8-$CH_2$ | 2.01 | | 31.8-31.2 |
| 9-$CH_2$ | 2.01 | | 31.8-31.2 |
| 10-CH | 5.36 | | 129.7-129.5 |
| 11-CH | 5.36 | | 129.7-129.5 |
| 12-$CH_2$ | 2.01 | | 31.8-31.2 |
| 13-$CH_2$ | 2.01 | | 31.8-31.2 |
| 14-CH | 5.36 | | 129.7-129.5 |
| 15-CH | 5.36 | | 129.7-129.5 |
| 16-$CH_2$ | 2.01 | | 31.8-31.2 |
| 17-$CH_2$ | 2.01 | | 31.8-31.2 |
| 18-CH | 5.39 | | 126.1 |
| 19-CH | 5.42 | | 131.6 |
| 20-$CH_2$ | 2.17, 1.98 | | 39.1 |
| 21-CH | 3.82 | m, 17.43 with Hax and 6 with Heq, 14 | 68.2 |
| 21-OH | nd | | - |
| 22-$CH_2$ | 1.46 ($H_{eq}$), 1.00 ($H_{ax}$) | m | 37.9 |
| 23-CH | 3.95 | Dq,17.9 with Hax, 6.5 with 24 and 6 with Heq | 64.4 |
| 24-$CH_3$ | 1.06 | d, 6.5 | 22.0 |
| **Pyruvate** | | | |
| 1'-CO | - | | 172.8 |
| 2'-qC | - | | 97.8 |
| 3'-$CH_3$ | 1.44 | | 17.4 |

**Table S7. Molecular formula, calculated *m/z*, observed *m/z* and error values (in Da and ppm) of fragment ions of chitinimine I/III and chitinimine II.** The corresponding spectrum is shown in Figure S26. nd = not determinable.

| | Molecular formula | Calculated *m/z* | Observed *m/z* | Error (Da) | Error (ppm) |
|---|---|---|---|---|---|
| **Chitinimine I/III** | | | | | |
| | $C_{49}H_{78}N_{6}O_{10}$ | 911.58518 | 911.5805 | 0.00468 | 5.1339 |
| | $C_{46}H_{76}N_{6}O_{8}$ | 841.57970 | 841.5752 | 0.00450 | 5.3471 |
| | $C_{46}H_{74}N_{6}O_{7}$ | 823.56914 | 823.5647 | 0.00444 | 5.3391 |
| | $C_{43}H_{69}N_{5}O_{10}$ (y1) | 816.51168 | 816.5118 | 0.00012 | 0.1470 |
| | $C_{38}H_{61}N_{3}O_{8}$ (y2) | 688.45311 | 688.4499 | 0.00321 | 4.6626 |
| | $C_{33}H_{52}N_{2}O_{6}$ (y3) | 573.38979 | 573.3859 | 0.00389 | 6.7842 |
| | $C_{27}H_{41}N_{1}O_{5}$ (y4) | 460.30573 | 460.3027 | 0.00303 | 6.5826 |
| | $C_{22}H_{39}N_{5}O_{6}$ (b4) | 470.29729 | 470.2936 | 0.00369 | 7.8461 |
| | $C_{16}H_{28}N_{4}O_{5}$ (b3) | 357.21323 | 357.2103 | 0.00293 | 8.2024 |
| | $C_{11}H_{19}N_{3}O_{3}$ (b2) | 242.14991 | 242.1471 | 0.00281 | 11.6044 |
| | $C_{6}H_{11}N_{1}O_{1}$ (b1) | 114.09134 | nd | - | - |
| **Chitinimine II** | | | | | |
| | $C4_{8}H_{76}N_{6}O_{10}$ | 897.56953 | 897.5654 | 0.00413 | 4.6013 |
| | $C_{45}H_{74}N_{6}O_{8}$ | 827.56405 | 827.5598 | 0.00425 | 5.1356 |
| | $C_{45}H_{72}N_{6}O_{7}$ | 809.55349 | 809.5492 | 0.00429 | 5.2992 |
| | $C_{42}H_{67}N_{5}O_{10}$ (y1) | 802.49603 | 802.4920 | 0.00403 | 5.0218 |
| | $C_{37}H_{59}N_{3}O_{8}$ (y2) | 674.43746 | n.d. | - | - |
| | $C_{32}H_{50}N_{2}O_{6}$ (y3) | 559.37414 | 559.3705 | 0.00364 | 6.0573 |
| | $C_{27}H_{41}N_{1}O_{5}$ (y4) | 460.30573 | 460.3020 | 0.00373 | 8.1033 |
| | $C_{21}H_{37}N_{5}O_{6}$ (b4) | 456.28164 | 456.2773 | 0.00434 | 9.557 |
| | $C_{16}H_{28}N_{4}O_{5}$ (b3) | 357.21323 | 357.2108 | 0.00243 | 6.8027 |
| | $C_{11}H_{19}N_{3}O_{3}$ (b2) | 242.14991 | 242.1476 | 0.00231 | 9.5395 |
| | $C_{6}H_{11}N_{1}O_{1}$ (b1) | 114.09134 | nd | - | - |

**Table S8. Experimental chemical shift values for the C3 position of chitinimine I, with the DFT-calculated values of each conformer.** expt=experimental, wavg=weighted average.

| expt. | *SS* | | | *RS* | *SR* | | | *RR* | avg *S* | avg *R* |
|---|---|---|---|---|---|---|---|---|---|---|
| [ppm] | CONF4 | CONF5 | wavg(*SS*) | | CONF24 | CONF26 | wavg(*SR*) | | *SS*+*SR* | *RS*+*RR* |
| 45.70 | 43.84 | 45.41 | 44.14 | 51.68 | 45.86 | 45.23 | 45.80 | 45.29 | 44.97 | 48.49 |
| 3.80 | 4.26 | 4.09 | 4.23 | 3.97 | 4.13 | 4.04 | 4.12 | 4.74 | 4.18 | 4.36 |

**Table S9. Predicted amino acid substrate specificities of the adenylation (A) domains in the chitinimine NRPS modules.** Overview of the predicted substrate binding residues in each A domain, along with specificity predictions obtained using antiSMASH and PARAS.[44] Where multiple predictions were given, the actual amino acid incorporated into the chitinimines is highlighted in bold.

| | | |
|---|---|---|
| **ChtnA_A1** | Predicted binding residues | DIWQFGLILK |
| | AntiSMASH prediction | X |
| | PARAS prediction | 1. Ala (0.131) **2. Leu (0.130)** |
| | Actual amino acid incorporated | Leu |
| **ChtnB_A2** | Predicted binding residues | DAQDLGVVDK |
| | AntiSMASH prediction | Gln |
| | PARAS prediction | **1. Gln (0.494)** |
| | Actual amino acid incorporated | Gln |
| **ChtnB_A3** | Predicted binding residues | DVWHFSLIEK |
| | AntiSMASH prediction | Ser |
| | PARAS prediction | **1. Ala (0.478)** |
| | Actual amino acid incorporated | Ala |
| **ChtnC_A4** | Predicted binding residues | DALFMGVVLK |
| | AntiSMASH prediction | Ile |
| | PARAS prediction | **1. Leu (0.338) 2. Valine (0.183) 3. Isoleucine (0.156)** |
| | Actual amino acids incorporated | Leu, Val and (*allo*-)Ile |

**Table S10. Condensation (C) domains used for phylogenetic analysis of the C domains within ChtnA-C.** Compilation of 172 C domains from the NaPDoS database, including the name and class of each C domain, their associated protein, MiBiG and PubMed IDs, the metabolic product and type of their cognate BGC, species name and strain, and gene product annotation.[45] na = not annotated.

| BGC product | BGC type | Domain name | Domain class | Genbank protein ID | MIBiG ID | PubMed ID | Species name | Strain | Gene product name |
|---|---|---|---|---|---|---|---|---|---|
| actinomycin | NRPS | actinomycin_C04_DCL | DCL | ADG27359 | BGC0000296 | 20304989 | *Streptomyces anulatus* | ATCC 11523 | *AcmC* |
| actinomycin | NRPS | actinomycin_C03_epimerization | epimerization | ADG27358 | BGC0000296 | 20304989 | *Streptomyces anulatus* | ATCC 11523 | *AcmB* |
| actinomycin | NRPS | actinomycin_C02_LCL | LCL | ADG27358 | BGC0000296 | 20304989 | *Streptomyces anulatus* | ATCC 11523 | *AcmB* |
| actinomycin | NRPS | actinomycin_C05_LCL | LCL | ADG27359 | BGC0000296 | 20304989 | *Streptomyces anulatus* | ATCC 11523 | *AcmC* |
| actinomycin | NRPS | actinomycin_C06_LCL | LCL | ADG27359 | BGC0000296 | 20304989 | *Streptomyces anulatus* | ATCC 11523 | *AcmC* |
| actinomycin | NRPS | actinomycin_C01_starter | starter | ADG27358 | BGC0000296 | 20304989 | *Streptomyces anulatus* | ATCC 11523 | *AcmB* |
| anabaenopeptilide | NRPS | anabaenopeptilide_C01_LCL | LCL | CAC01603 | na | 10931313 | *Anabaena sp.* | str 90 | *AdpA* |
| anabaenopeptilide | NRPS | anabaenopeptilide_C02_LCL | LCL | CAC01604 | na | 10931313 | *Anabaena sp.* | str 90 | *AdpB* |
| anabaenopeptilide | NRPS | anabaenopeptilide_C03_LCL | LCL | CAC01604 | na | 10931313 | *Anabaena sp.* | str 90 | *AdpB* |
| anabaenopeptilide | NRPS | anabaenopeptilide_C04_LCL | LCL | CAC01604 | na | 10931313 | *Anabaena sp.* | str 90 | *AdpB* |
| anabaenopeptilide | NRPS | anabaenopeptilide_C05_LCL | LCL | CAC01604 | na | 10931313 | *Anabaena sp.* | str 90 | *AdpB* |
| anabaenopeptilide | NRPS | anabaenopeptilide_C06_LCL | LCL | CAC01606 | na | 10931313 | *Anabaena sp.* | str 90 | *AdpD* |
| arthrofactin | NRPS | arthrofactin_C02_DCL | DCL | BAC67534 | BGC0000305 | 14522057 | *Pseudomonas sp.* | MIS38 | *ArfA* |
| arthrofactin | NRPS | arthrofactin_C03_DCL | DCL | BAC67535 | BGC0000305 | 14522057 | *Pseudomonas sp.* | MIS38 | *ArfB* |
| arthrofactin | NRPS | arthrofactin_C04_DCL | DCL | BAC67535 | BGC0000305 | 14522057 | *Pseudomonas sp.* | MIS38 | *ArfB* |
| arthrofactin | NRPS | arthrofactin_C05_DCL | DCL | BAC67535 | BGC0000305 | 14522057 | *Pseudomonas sp.* | MIS38 | *ArfB* |
| arthrofactin | NRPS | arthrofactin_C06_DCL | DCL | BAC67535 | BGC0000305 | 14522057 | *Pseudomonas sp.* | MIS38 | *ArfB* |
| arthrofactin | NRPS | arthrofactin_C07_DCL | DCL | BAC67536 | BGC0000305 | 14522057 | *Pseudomonas sp.* | MIS38 | *ArfC* |
| arthrofactin | NRPS | arthrofactin_C09_DCL | DCL | BAC67536 | BGC0000305 | 14522057 | *Pseudomonas sp.* | MIS38 | *ArfC* |
| arthrofactin | NRPS | arthrofactin_C08_LCL | LCL | BAC67536 | BGC0000305 | 14522057 | *Pseudomonas sp.* | MIS38 | *ArfC* |
| arthrofactin | NRPS | arthrofactin_C10_LCL | LCL | BAC67536 | BGC0000305 | 14522057 | *Pseudomonas sp.* | MIS38 | *ArfC* |
| arthrofactin | NRPS | arthrofactin_C11_LCL | LCL | BAC67536 | BGC0000305 | 14522057 | *Pseudomonas sp.* | MIS38 | *ArfC* |
| arthrofactin | NRPS | arthrofactin_C01_starter | starter | BAC67534 | BGC0000305 | 14522057 | *Pseudomonas sp.* | MIS38 | *ArfA* |
| bacillibactin | NRPS | bacillibactin_C02_LCL | LCL | CAB15186 | BGC0000309 | 11112781 | *Bacillus subtilis subsp. subtilis* | str 168 | *DhbF* |
| bacillibactin | NRPS | bacillibactin_C01_starter | starter | CAB15186 | BGC0000309 | 11112781 | *Bacillus subtilis subsp. subtilis* | str 168 | *DhbF* |
| bacitracin | NRPS | bacitracin_C01_cyclization | cyclization | AAC06346 | BGC0000310 | 9427658 | *Bacillus licheniformis* | ATCC 10716 | *BacA* |
| bacitracin | NRPS | bacitracin_C05_DCL | DCL | AAC06346 | BGC0000310 | 9427658 | *Bacillus licheniformis* | ATCC 10716 | *BacA* |
| bacitracin | NRPS | bacitracin_C09_DCL | DCL | AAC06348 | BGC0000310 | 9427658 | *Bacillus licheniformis* | ATCC 10716 | *BacC* |
| bacitracin | NRPS | bacitracin_C12_DCL | DCL | AAC06348 | BGC0000310 | 9427658 | *Bacillus licheniformis* | ATCC 10716 | *BacC* |
| bacitracin | NRPS | bacitracin_C15_DCL | DCL | AAC06348 | BGC0000310 | 9427658 | *Bacillus licheniformis* | ATCC 10716 | *BacC* |
| bacitracin | NRPS | bacitracin_C04_epimerization | epimerization | AAC06346 | BGC0000310 | 9427658 | *Bacillus licheniformis* | ATCC 10716 | *BacA* |
| bacitracin | NRPS | bacitracin_C08_epimerization | epimerization | AAC06347 | BGC0000310 | 9427658 | *Bacillus licheniformis* | ATCC 10716 | *BacB* |
| bacitracin | NRPS | bacitracin_C11_epimerization | epimerization | AAC06348 | BGC0000310 | 9427658 | *Bacillus licheniformis* | ATCC 10716 | *BacC* |
| bacitracin | NRPS | bacitracin_C14_epimerization | epimerization | AAC06348 | BGC0000310 | 9427658 | *Bacillus licheniformis* | ATCC 10716 | *BacC* |
| bacitracin | NRPS | bacitracin_C02_LCL | LCL | AAC06346 | BGC0000310 | 9427658 | *Bacillus licheniformis* | ATCC 10716 | *BacA* |
| bacitracin | NRPS | bacitracin_C03_LCL | LCL | AAC06346 | BGC0000310 | 9427658 | *Bacillus licheniformis* | ATCC 10716 | *BacA* |
| bacitracin | NRPS | bacitracin_C06_LCL | LCL | AAC06347 | BGC0000310 | 9427658 | *Bacillus licheniformis* | ATCC 10716 | *BacB* |
| bacitracin | NRPS | bacitracin_C07_LCL | LCL | AAC06347 | BGC0000310 | 9427658 | *Bacillus licheniformis* | ATCC 10716 | *BacB* |
| bacitracin | NRPS | bacitracin_C10_LCL | LCL | AAC06348 | BGC0000310 | 9427658 | *Bacillus licheniformis* | ATCC 10716 | BacC |
| balhimycin | NRPS | balhimycin_C01_DCL | DCL | CAC48360 | BGC0000311 | 11932455 | *Amycolatopsis balhimycina* | DSM 5908 | BpsA |
| balhimycin | NRPS | balhimycin_C03_DCL | DCL | CAC48360 | BGC0000311 | 11932455 | *Amycolatopsis balhimycina* | DSM 5908 | BpsA |
| balhimycin | NRPS | balhimycin_C04_DCL | DCL | CAC48361 | BGC0000311 | 11932455 | *Amycolatopsis balhimycina* | DSM 5908 | BpsB |

| | | | | | | | | | |
|---|---|---|---|---|---|---|---|---|---|
| balhimycin | NRPS | balhimycin_C06_DCL | DCL | CAC48361 | BGC0000311 | 11932455 | *Amycolatopsis balhimycina* | DSM 5908 | BpsB |
| balhimycin | NRPS | balhimycin_C08_DCL | DCL | CAC48361 | BGC0000311 | 11932455 | *Amycolatopsis balhimycina* | DSM 5908 | BpsB |
| balhimycin | NRPS | balhimycin_C09_DCL | DCL | CAC48362 | BGC0000311 | 11932455 | *Amycolatopsis balhimycina* | DSM 5908 | BpsC |
| balhimycin | NRPS | balhimycin_C02_epimerization | epimerization | CAC48360 | BGC0000311 | 11932455 | *Amycolatopsis balhimycina* | DSM 5908 | BpsA |
| balhimycin | NRPS | balhimycin_C05_epimerization | epimerization | CAC48361 | BGC0000311 | 11932455 | *Amycolatopsis balhimycina* | DSM 5908 | BpsB |
| balhimycin | NRPS | balhimycin_C07_epimerization | epimerization | CAC48361 | BGC0000311 | 11932455 | *Amycolatopsis balhimycina* | DSM 5908 | BpsB |
| balhimycin | NRPS | balhimycin_C10_LCL | LCL | CAC48362 | BGC0000311 | 11932455 | *Amycolatopsis balhimycina* | DSM 5908 | BpsC |
| bleomycin | PKS-NRPS | bleomycin_C01_condensation | condensation | AAG02355 | BGC0000963 | 11048953 | *Streptomyces verticillus* | ATCC 15003 | BlmX |
| bleomycin | PKS-NRPS | bleomycin_C03_condensation | condensation | AAG02356 | BGC0000963 | 11048953 | *Streptomyces verticillus* | ATCC 15003 | BlmIX |
| bleomycin | PKS-NRPS | bleomycin_C04_condensation | condensation | AAG02358 | BGC0000963 | 11048953 | *Streptomyces verticillus* | ATCC 15003 | BlmVII |
| bleomycin | PKS-NRPS | bleomycin_C05_condensation | condensation | AAG02359 | BGC0000963 | 11048953 | *Streptomyces verticillus* | ATCC 15003 | BlmVI |
| bleomycin | PKS-NRPS | bleomycin_C08_condensation | condensation | AAG02364 | BGC0000963 | 11048953 | *Streptomyces verticillus* | ATCC 15003 | BlmIV |
| bleomycin | PKS-NRPS | bleomycin_C09_cyclization | cyclization | AAG02364 | BGC0000963 | 11048953 | *Streptomyces verticillus* | ATCC 15003 | BlmIV |
| bleomycin | PKS-NRPS | bleomycin_C10_cyclization | cyclization | AAG02364 | BGC0000963 | 11048953 | *Streptomyces verticillus* | ATCC 15003 | BlmIV |
| bleomycin | PKS-NRPS | bleomycin_C07_DCL | DCL | AAG02360 | BGC0000963 | 11048953 | *Streptomyces verticillus* | ATCC 15003 | BlmV |
| bleomycin | PKS-NRPS | bleomycin_C02_modifiedAA | modified amino acid | AAG02355 | BGC0000963 | 11048953 | *Streptomyces verticillus* | ATCC 15003 | BlmX |
| bleomycin | PKS-NRPS | bleomycin_C06_modifiedAA | modified amino acid | AAG02359 | BGC0000963 | 11048953 | *Streptomyces verticillus* | ATCC 15003 | BlmVI |
| C-1027 | PKS-NRPS | C1027_C02_LCL | LCL | AAL06678 | BGC0000965 | 12183628 | *Streptomyces globisporus* | C-1027 | SgcC5 |
| calcium-dependent antibiotic | NRPS | calciumdependentantibiotic_C05_DCL | DCL | CAB38518 | BGC0000315 | 12445768 | *Streptomyces coelicolor* | A3(2) | CdaPS1 |
| calcium-dependent antibiotic | NRPS | calciumdependentantibiotic_C08_DCL | DCL | CAB38517 | BGC0000315 | 12445768 | *Streptomyces coelicolor* | A3(2) | CdaPS2 |
| calcium-dependent antibiotic | NRPS | calciumdependentantibiotic_C12_DCL | DCL | CAD55498 | BGC0000315 | 12445768 | *Streptomyces coelicolor* | A3(2) | CdaPS3 |
| calcium-dependent antibiotic | NRPS | calciumdependentantibiotic_C04_epimerization | epimerization | CAB38518 | BGC0000315 | 12445768 | *Streptomyces coelicolor* | A3(2) | CdaPS1 |
| calcium-dependent antibiotic | NRPS | calciumdependentantibiotic_C11_epimerization | epimerization | CAB38517 | BGC0000315 | 12445768 | *Streptomyces coelicolor* | A3(2) | CdaPS2 |
| calcium-dependent antibiotic | NRPS | calciumdependentantibiotic_C02_LCL | LCL | CAB38518 | BGC0000315 | 12445768 | *Streptomyces coelicolor* | A3(2) | CdaPS1 |
| calcium-dependent antibiotic | NRPS | calciumdependentantibiotic_C03_LCL | LCL | CAB38518 | BGC0000315 | 12445768 | *Streptomyces coelicolor* | A3(2) | CdaPS1 |
| calcium-dependent antibiotic | NRPS | calciumdependentantibiotic_C06_LCL | LCL | CAB38518 | BGC0000315 | 12445768 | *Streptomyces coelicolor* | A3(2) | CdaPS1 |
| calcium-dependent antibiotic | NRPS | calciumdependentantibiotic_C07_LCL | LCL | CAB38518 | BGC0000315 | 12445768 | *Streptomyces coelicolor* | A3(2) | CdaPS1 |
| calcium-dependent antibiotic | NRPS | calciumdependentantibiotic_C09_LCL | LCL | CAB38517 | BGC0000315 | 12445768 | *Streptomyces coelicolor* | A3(2) | CdaPS2 |
| calcium-dependent antibiotic | NRPS | calciumdependentantibiotic_C10_LCL | LCL | CAB38517 | BGC0000315 | 12445768 | *Streptomyces coelicolor* | A3(2) | CdaPS2 |
| calcium-dependent antibiotic | NRPS | calciumdependentantibiotic_C13_LCL | LCL | CAD55498 | BGC0000315 | 12445768 | *Streptomyces coelicolor* | A3(2) | CdaPS3 |
| calcium-dependent antibiotic | NRPS | calciumdependentantibiotic_C01_starter | starter | CAB38518 | BGC0000315 | 12445768 | *Streptomyces coelicolor* | A3(2) | CdaPS1 |
| chloroeremomycin | NRPS | chloroeremomycin_C01_DCL | DCL | CAA11794 | na | 10716695 | *Amycolotopsis orientali* | PCZA363 | PCZA363-3 |
| chloroeremomycin | NRPS | chloroeremomycin_C02_DCL | DCL | CAA11794 | na | 10716695 | *Amycolotopsis orientali* | PCZA363 | PCZA363-3 |
| chloroeremomycin | NRPS | chloroeremomycin_C03_DCL | DCL | CAA11795 | na | 10716695 | *Amycolotopsis orientali* | PCZA363 | PCZA363-4 |
| chloroeremomycin | NRPS | chloroeremomycin_C04_DCL | DCL | CAA11795 | na | 10716695 | *Amycolotopsis orientali* | PCZA363 | PCZA363-4 |
| chloroeremomycin | NRPS | chloroeremomycin_C05_DCL | DCL | CAA11795 | na | 10716695 | *Amycolotopsis orientali* | PCZA363 | PCZA363-4 |
| chloroeremomycin | NRPS | chloroeremomycin_C06_DCL | DCL | CAA11796 | na | 10716695 | *Amycolotopsis orientali* | PCZA363 | PCZA363-5 |
| cinnabaramide | PKS-NRPS | cinnabaramide_C01_LCL | LCL | CBW54671 | BGC0000971 | 21387511 | *Streptomyces cinnabarigriseus* | JS360 | CinA |
| complestatin | NRPS | complestatin_C01_DCL | DCL | AAK81824 | BGC0000326 | 11447274 | *Streptomyces lavendulae* | | ComA |

| | | | | | | | | | |
|---|---|---|---|---|---|---|---|---|---|
| complestatin | NRPS | complestatin_C02_DCL | DCL | AAK81825 | BGC0000326 | 11447274 | *Streptomyces lavendulae* | | ComB |
| complestatin | NRPS | complestatin_C03_DCL | DCL | AAK81826 | BGC0000326 | 11447274 | *Streptomyces lavendulae* | | ComC |
| complestatin | NRPS | complestatin_C05_DCL | DCL | AAK81826 | BGC0000326 | 11447274 | *Streptomyces lavendulae* | | ComC |
| complestatin | NRPS | complestatin_C07_DCL | DCL | AAK81826 | BGC0000326 | 11447274 | *Streptomyces lavendulae* | | ComC |
| complestatin | NRPS | complestatin_C09_DCL | DCL | AAK81827 | BGC0000326 | 11447274 | *Streptomyces lavendulae* | | ComD |
| complestatin | NRPS | complestatin_C04_epimerization | epimerization | AAK81826 | BGC0000326 | 11447274 | *Streptomyces lavendulae* | | ComC |
| complestatin | NRPS | complestatin_C06_epimerization | epimerization | AAK81826 | BGC0000326 | 11447274 | *Streptomyces lavendulae* | | ComC |
| complestatin | NRPS | complestatin_C08_epimerization | epimerization | AAK81826 | BGC0000326 | 11447274 | *Streptomyces lavendulae* | | ComC |
| complestatin | NRPS | complestatin_C10_LCL | LCL | AAK81827 | BGC0000326 | 11447274 | *Streptomyces lavendulae* | | ComD |
| cyclomarin | NRPS | cyclomarin_C01_LCL | LCL | ABW00331 | BGC0000333 | 18331040 | *Salinispora arenicola* | CNS-205 | CymA |
| cyclomarin | NRPS | cyclomarin_C02_LCL | LCL | ABW00331 | BGC0000333 | 18331040 | *Salinispora arenicola* | CNS-205 | CymA |
| cyclomarin | NRPS | cyclomarin_C03_LCL | LCL | ABW00331 | BGC0000333 | 18331040 | *Salinispora arenicola* | CNS-205 | CymA |
| cyclomarin | NRPS | cyclomarin_C04_LCL | LCL | ABW00331 | BGC0000333 | 18331040 | *Salinispora arenicola* | CNS-205 | CymA |
| cyclomarin | NRPS | cyclomarin_C05_LCL | LCL | ABW00331 | BGC0000333 | 18331040 | *Salinispora arenicola* | CNS-205 | CymA |
| cyclomarin | NRPS | cyclomarin_C06_LCL | LCL | ABW00331 | BGC0000333 | 18331040 | *Salinispora arenicola* | CNS-205 | CymA |
| cyclomarin | NRPS | cyclomarin_C01_LCL | LCL | ABW00331 | BGC0000333 | 18331040 | *Salinispora arenicola* | CNS-205 | CymA |
| cyclosporin | NRPS | cyclosporin_C12_dual | dual | NA1 | BGC0000334 | 8376400 | *Tolypocladium inflatum* | NRRL8044 | na |
| cyclosporin | NRPS | cyclosporin_C01_LCL | LCL | NA1 | BGC0000334 | 8376400 | *Tolypocladium inflatum* | NRRL8044 | na |
| cyclosporin | NRPS | cyclosporin_C02_LCL | LCL | NA1 | BGC0000334 | 8376400 | *Tolypocladium inflatum* | NRRL8044 | na |
| cyclosporin | NRPS | cyclosporin_C03_LCL | LCL | NA1 | BGC0000334 | 8376400 | *Tolypocladium inflatum* | NRRL8044 | na |
| cyclosporin | NRPS | cyclosporin_C04_LCL | LCL | NA1 | BGC0000334 | 8376400 | *Tolypocladium inflatum* | NRRL8044 | na |
| cyclosporin | NRPS | cyclosporin_C05_LCL | LCL | NA1 | BGC0000334 | 8376400 | *Tolypocladium inflatum* | NRRL8044 | na |
| cyclosporin | NRPS | cyclosporin_C06_LCL | LCL | NA1 | BGC0000334 | 8376400 | *Tolypocladium inflatum* | NRRL8044 | na |
| cyclosporin | NRPS | cyclosporin_C07_LCL | LCL | NA1 | BGC0000334 | 8376400 | *Tolypocladium inflatum* | NRRL8044 | na |
| cyclosporin | NRPS | cyclosporin_C08_LCL | LCL | NA1 | BGC0000334 | 8376400 | *Tolypocladium inflatum* | NRRL8044 | na |
| cyclosporin | NRPS | cyclosporin_C09_LCL | LCL | NA1 | BGC0000334 | 8376400 | *Tolypocladium inflatum* | NRRL8044 | na |
| cyclosporin | NRPS | cyclosporin_C10_LCL | LCL | NA1 | BGC0000334 | 8376400 | *Tolypocladium inflatum* | NRRL8044 | na |
| cyclosporin | NRPS | cyclosporin_C11_LCL | LCL | NA1 | BGC0000334 | 8376400 | *Tolypocladium inflatum* | NRRL8044 | na |
| enniatin | NRPS | enniatin_C02_dual | dual | CAA79245 | BGC0000342 | 8483420 | *Fusarium scirpi* | ETH1536J5 | Esyn1 |
| enniatin | NRPS | enniatin_C01_LCL | LCL | CAA79245 | BGC0000342 | 8483420 | *Fusarium scirpi* | ETH1536J5 | Esyn1 |
| enterobactin | NRPS | enterobactin_C01_starter | starter | ADB98044 | BGC0000343 | 10688898 | *Escherichia coli* | chi7122 | EntF |
| exochelin | NRPS | exochelin_C01_DCL | DCL | AAC82549 | BGC0000351 | 9720878 | *Mycobacterium smegmatis* | MC2 155 | FxbB |
| exochelin | NRPS | exochelin_C02_DCL | DCL | AAC82549 | BGC0000351 | 9720878 | *Mycobacterium smegmatis* | MC2 155 | FxbB |
| exochelin | NRPS | exochelin_C03_DCL | DCL | AAC82550 | BGC0000351 | 9720878 | *Mycobacterium smegmatis* | MC2 155 | FxbC |
| exochelin | NRPS | exochelin_C04_DCL | DCL | AAC82550 | BGC0000351 | 9720878 | *Mycobacterium smegmatis* | MC2 155 | FxbC |
| exochelin | NRPS | exochelin_C05_LCL | LCL | AAC82550 | BGC0000351 | 9720878 | *Mycobacterium smegmatis* | MC2 155 | FxbC |
| fengycin | NRPS | fengycin_C01_DCL | DCL | NA1 | BGC0001095 | 10438779 | *Bacillus subtilis* | | na1 |
| fengycin | NRPS | fengycin_C05_DCL | DCL | NA2 | BGC0001095 | 10438779 | *Bacillus subtilis* | | na2 |
| fengycin | NRPS | fengycin_C09_DCL | DCL | NA4 | BGC0001095 | 10438779 | *Bacillus subtilis* | | na4 |
| fengycin | NRPS | fengycin_C12_DCL | DCL | NA5 | BGC0001095 | 10438779 | *Bacillus subtilis* | | na5 |
| fengycin | NRPS | fengycin_C04_epimerization | epimerization | NA1 | BGC0001095 | 10438779 | *Bacillus subtilis* | | na1 |
| fengycin | NRPS | fengycin_C08_epimerization | epimerization | NA3 | BGC0001095 | 10438779 | *Bacillus subtilis* | | na3 |
| fengycin | NRPS | fengycin_C11_epimerization | epimerization | NA4 | BGC0001095 | 10438779 | *Bacillus subtilis* | | na4 |
| fengycin | NRPS | fengycin_C14_epimerization | epimerization | NA5 | BGC0001095 | 10438779 | *Bacillus subtilis* | | na5 |
| fengycin | NRPS | fengycin_C02_LCL | LCL | NA1 | BGC0001095 | 10438779 | *Bacillus subtilis* | | na1 |
| fengycin | NRPS | fengycin_C03_LCL | LCL | NA1 | BGC0001095 | 10438779 | *Bacillus subtilis* | | na1 |

| | | | | | | | | | |
|---|---|---|---|---|---|---|---|---|---|
| fengycin | NRPS | fengycin_C07_LCL | LCL | NA3 | BGC0001095 | 10438779 | *Bacillus subtilis* | | na3 |
| fengycin | NRPS | fengycin_C10_LCL | LCL | NA4 | BGC0001095 | 10438779 | *Bacillus subtilis* | | na4 |
| fengycin | NRPS | fengycin_C13_LCL | LCL | NA5 | BGC0001095 | 10438779 | *Bacillus subtilis* | | na5 |
| fengycin | NRPS | fengycin_C06_starter | starter | NA3 | BGC0001095 | 10438779 | *Bacillus subtilis* | | na3 |
| fengycin | NRPS | fengycin_C01_DCL | DCL | NA1 | BGC0001095 | 10438779 | *Bacillus subtilis* | | na1 |
| gramicidin | NRPS | gramicidin_C02_DCL | DCL | CAA43838 | BGC0000367 | 1560782 | *Brevibacillus brevis* | ATCC 9999 | GrsB |
| gramicidin | NRPS | gramicidin_C01_epimerization | epimerization | CAA33603 | BGC0000367 | 1560782 | *Brevibacillus brevis* | ATCC 9999 | GrsA |
| gramicidin | NRPS | gramicidin_C03_LCL | LCL | CAA43838 | BGC0000367 | 1560782 | *Brevibacillus brevis* | ATCC 9999 | GrsB |
| gramicidin | NRPS | gramicidin_C04_LCL | LCL | CAA43838 | BGC0000367 | 1560782 | *Brevibacillus brevis* | ATCC 9999 | GrsB |
| gramicidin | NRPS | gramicidin_C05_LCL | LCL | CAA43838 | BGC0000367 | 1560782 | *Brevibacillus brevis* | ATCC 9999 | GrsB |
| HC-toxin | NRPS | HCtoxin_C02_dual | dual | AAA33023 | na | 1281482 | *Bipolaris zeicola* | SB111 | HTS1 |
| HC-toxin | NRPS | HCtoxin_C03_dual | dual | AAA33023 | na | 1281482 | *Bipolaris zeicola* | SB111 | HTS1 |
| HC-toxin | NRPS | HCtoxin_C04_dual | dual | AAA33023 | na | 1281482 | *Bipolaris zeicola* | SB111 | HTS1 |
| HC-toxin | NRPS | HCtoxin_C05_dual | dual | AAA33023 | na | 1281482 | *Bipolaris zeicola* | SB111 | HTS1 |
| HC-toxin | NRPS | HCtoxin_C01_epimerization | epimerization | AAA33023 | na | 1281482 | *Bipolaris zeicola* | SB111 | HTS1 |
| iturin | PKS-NRPS | iturin_C05_DCL | DCL | BAB69699 | BGC0001098 | 11591669 | *Bacillus subtilis* | RB14 | ItuB |
| iturin | PKS-NRPS | iturin_C07_DCL | DCL | BAB69699 | BGC0001098 | 11591669 | *Bacillus subtilis* | RB14 | ItuB |
| iturin | PKS-NRPS | iturin_C11_DCL | DCL | BAB69700 | BGC0001098 | 11591669 | *Bacillus subtilis* | RB14 | ItuC |
| iturin | PKS-NRPS | iturin_C04_epimerization | epimerization | BAB69699 | BGC0001098 | 11591669 | *Bacillus subtilis* | RB14 | ItuB |
| iturin | PKS-NRPS | iturin_C06_epimerization | epimerization | BAB69699 | BGC0001098 | 11591669 | *Bacillus subtilis* | RB14 | ItuB |
| iturin | PKS-NRPS | iturin_C10_epimerization | epimerization | BAB69700 | BGC0001098 | 11591669 | *Bacillus subtilis* | RB14 | ItuC |
| iturin | PKS-NRPS | iturin_C01_hybridC | hybrid C | BAB69698 | BGC0001098 | 11591669 | *Bacillus subtilis* | RB14 | ItuA |
| iturin | PKS-NRPS | iturin_C02_LCL | LCL | BAB69698 | BGC0001098 | 11591669 | *Bacillus subtilis* | RB14 | ItuA |
| iturin | PKS-NRPS | iturin_C03_LCL | LCL | BAB69698 | BGC0001098 | 11591669 | *Bacillus subtilis* | RB14 | ItuA |
| iturin | PKS-NRPS | iturin_C08_LCL | LCL | BAB69699 | BGC0001098 | 11591669 | *Bacillus subtilis* | RB14 | ItuB |
| iturin | PKS-NRPS | iturin_C09_LCL | LCL | BAB69699 | BGC0001098 | 11591669 | *Bacillus subtilis* | RB14 | ItuB |
| lichenysin | NRPS | lichenysin_C05_DCL | DCL | AAU39360 | BGC0000381 | 9864322 | *Bacillus licheniformis* | DSM 13 | LchAB |
| lichenysin | NRPS | lichenysin_C09_DCL | DCL | AAU39361 | BGC0000381 | 9864322 | *Bacillus licheniformis* | DSM 13 | LchAC |
| lichenysin | NRPS | lichenysin_C04_epimerization | epimerization | AAU39359 | BGC0000381 | 9864322 | *Bacillus licheniformis* | DSM 13 | LchAA |
| lichenysin | NRPS | lichenysin_C08_epimerization | epimerization | AAU39360 | BGC0000381 | 9864322 | *Bacillus licheniformis* | DSM 13 | LchAB |
| lichenysin | NRPS | lichenysin_C02_LCL | LCL | AAU39359 | BGC0000381 | 9864322 | *Bacillus licheniformis* | DSM 13 | LchAA |
| lichenysin | NRPS | lichenysin_C03_LCL | LCL | AAU39359 | BGC0000381 | 9864322 | *Bacillus licheniformis* | DSM 13 | LchAA |
| lichenysin | NRPS | lichenysin_C06_LCL | LCL | AAU39360 | BGC0000381 | 9864322 | *Bacillus licheniformis* | DSM 13 | LchAB |
| lichenysin | NRPS | lichenysin_C07_LCL | LCL | AAU39360 | BGC0000381 | 9864322 | *Bacillus licheniformis* | DSM 13 | LchAB |
| lichenysin | NRPS | lichenysin_C01_starter | starter | AAU39359 | BGC0000381 | 9864322 | *Bacillus licheniformis* | DSM 13 | LchAA |
| microcystin | PKS-NRPS | microcystin_C05_DCL | DCL | AAF00961 | BGC0001017 | 10788786 | *Microcystis aeruginosa* | PCC7806 | McyB |
| microcystin | PKS-NRPS | microcystin_C04_epimerization | epimerization | AAF00960 | BGC0001017 | 10788786 | *Microcystis aeruginosa* | PCC7806 | McyA |
| microcystin | PKS-NRPS | microcystin_C01_hybridC | hybrid C | AAF00958 | BGC0001017 | 10788786 | *Microcystis aeruginosa* | PCC7806 | McyE |
| microcystin | PKS-NRPS | microcystin_C02_LCL | LCL | AAF00958 | BGC0001017 | 10788786 | *Microcystis aeruginosa* | PCC7806 | McyE |
| microcystin | PKS-NRPS | microcystin_C06_LCL | LCL | AAF00961 | BGC0001017 | 10788786 | *Microcystis aeruginosa* | PCC7806 | McyB |
| microcystin | PKS-NRPS | microcystin_C07_LCL | LCL | AAF00962 | BGC0001017 | 10788786 | *Microcystis aeruginosa* | PCC7806 | McyC |
| microcystin | PKS-NRPS | microcystin_C03_modifiedAA | modified amino acid | AAF00960 | BGC0001017 | 10788786 | *Microcystis aeruginosa* | PCC7806 | McyA |
| mycosubtilin | PKS-NRPS | mycosubtilin_C05_DCL | DCL | AAF08796 | BGC0001103 | 10557314 | *Bacillus subtilis* | ATCC 6633 | MycB |
| mycosubtilin | PKS-NRPS | mycosubtilin_C07_DCL | DCL | AAF08796 | BGC0001103 | 10557314 | *Bacillus subtilis* | ATCC 6633 | MycB |
| mycosubtilin | PKS-NRPS | mycosubtilin_C11_DCL | DCL | AAF08797 | BGC0001103 | 10557314 | *Bacillus subtilis* | ATCC 6633 | MycC |
| mycosubtilin | PKS-NRPS | mycosubtilin_C04_epimerization | epimerization | AAF08796 | BGC0001103 | 10557314 | *Bacillus subtilis* | ATCC 6633 | MycB |

| | | | | | | | | | |
|---|---|---|---|---|---|---|---|---|---|
| mycosubtilin | PKS-NRPS | mycosubtilin_C06_epimerization | epimerization | AAF08796 | BGC0001103 | 10557314 | *Bacillus subtilis* | ATCC 6633 | MycB |
| mycosubtilin | PKS-NRPS | mycosubtilin_C10_epimerization | epimerization | AAF08797 | BGC0001103 | 10557314 | *Bacillus subtilis* | ATCC 6633 | MycC |
| mycosubtilin | PKS-NRPS | mycosubtilin_C01_hybridC | hybrid C | AAF08795 | BGC0001103 | 10557314 | *Bacillus subtilis* | ATCC 6633 | MycA |
| mycosubtilin | PKS-NRPS | mycosubtilin_C02_LCL | LCL | AAF08795 | BGC0001103 | 10557314 | *Bacillus subtilis* | ATCC 6633 | MycA |
| mycosubtilin | PKS-NRPS | mycosubtilin_C03_LCL | LCL | AAF08795 | BGC0001103 | 10557314 | *Bacillus subtilis* | ATCC 6633 | MycA |
| mycosubtilin | PKS-NRPS | mycosubtilin_C08_LCL | LCL | AAF08796 | BGC0001103 | 10557314 | *Bacillus subtilis* | ATCC 6633 | MycB |
| mycosubtilin | PKS-NRPS | mycosubtilin_C09_LCL | LCL | AAF08796 | BGC0001103 | 10557314 | *Bacillus subtilis* | ATCC 6633 | MycB |
| mycosubtilin | PKS-NRPS | mycosubtilin_C05_DCL | DCL | AAF08796 | BGC0001103 | 10557314 | *Bacillus subtilis* | ATCC 6633 | MycB |
| nodularin | PKS-NRPS | nodularin_C01_hybridC | hybrid C | AAO64407 | na | 15528492 | *Nodularia spumigena* | NSOR10 | NdaF |
| nodularin | PKS-NRPS | nodularin_C02_LCL | LCL | AAO64407 | na | 15528492 | *Nodularia spumigena* | NSOR10 | NdaF |
| nodularin | PKS-NRPS | nodularin_C04_LCL | LCL | AAO64402 | na | 15528492 | *Nodularia spumigena* | NSOR10 | NdaB |
| nodularin | PKS-NRPS | nodularin_C03_modifiedAA | modified amino acid | AAO64403 | na | 15528492 | *Nodularia spumigena* | NSOR10 | NdaA |
| nostopeptolide | PKS-NRPS | nostopeptolide_C01_LCL | LCL | AAF15891 | BGC0001028 | 12853152 | *Nostoc sp.* | GSV224 | NosA |
| nostopeptolide | PKS-NRPS | nostopeptolide_C02_LCL | LCL | AAF15891 | BGC0001028 | 12853152 | *Nostoc sp.* | GSV224 | NosA |
| nostopeptolide | PKS-NRPS | nostopeptolide_C03_LCL | LCL | AAF15891 | BGC0001028 | 12853152 | *Nostoc sp.* | GSV224 | NosA |
| nostopeptolide | PKS-NRPS | nostopeptolide_C04_LCL | LCL | AAF15891 | BGC0001028 | 12853152 | *Nostoc sp.* | GSV224 | NosA |
| nostopeptolide | PKS-NRPS | nostopeptolide_C05_LCL | LCL | AAF17280 | BGC0001028 | 12853152 | *Nostoc sp.* | GSV224 | NosC |
| nostopeptolide | PKS-NRPS | nostopeptolide_C06_LCL | LCL | AAF17280 | BGC0001028 | 12853152 | *Nostoc sp.* | GSV224 | NosC |
| nostopeptolide | PKS-NRPS | nostopeptolide_C07_LCL | LCL | AAF17280 | BGC0001028 | 12853152 | *Nostoc sp.* | GSV224 | NosC |
| nostopeptolide | PKS-NRPS | nostopeptolide_C08_LCL | LCL | AAF17281 | BGC0001028 | 12853152 | *Nostoc sp.* | GSV224 | NosD |
| nostopeptolide | PKS-NRPS | nostopeptolide_C09_LCL | LCL | AAF17281 | BGC0001028 | 12853152 | *Nostoc sp.* | GSV224 | NosD |
| penicillin | NRPS | penicillin_C01_DCL | DCL | ABA70582 | BGC0000404 | 16713314 | *Penicillium chrysogenum* | AS-P-78 | PcbAB |
| penicillin | NRPS | penicillin_C02_LCL | LCL | ABA70582 | BGC0000404 | 16713314 | *Penicillium chrysogenum* | AS-P-78 | PcbAB |
| pristinamycin | NRPS | pristinamycin_C04_DCL | DCL | CBW45647 | na | 10449311 | *Streptomyces pristinaespiralis* | Pr11 | SnbDE |
| pristinamycin | NRPS | pristinamycin_C03_epimerization | epimerization | CBW45637 | na | 10449311 | *Streptomyces pristinaespiralis* | Pr11 | SnbC |
| pristinamycin | NRPS | pristinamycin_C02_LCL | LCL | CBW45637 | na | 10449311 | *Streptomyces pristinaespiralis* | Pr11 | SnbC |
| pristinamycin | NRPS | pristinamycin_C05_LCL | LCL | CBW45647 | na | 10449311 | *Streptomyces pristinaespiralis* | Pr11 | SnbDE |
| pristinamycin | NRPS | pristinamycin_C06_LCL | LCL | CBW45647 | na | 10449311 | *Streptomyces pristinaespiralis* | Pr11 | SnbDE |
| pristinamycin | NRPS | pristinamycin_C07_LCL | LCL | CBW45647 | na | 10449311 | *Streptomyces pristinaespiralis* | Pr11 | SnbDE |
| pristinamycin | NRPS | pristinamycin_C01_starter | starter | CBW45637 | na | 10449311 | *Streptomyces pristinaespiralis* | Pr11 | SnbC |
| pyochelin | NRPS | pyochelin_C01_cyclization | cyclization | AAC83656 | BGC0000412 | 10555976 | *Pseudomonas aeruginosa* | PAO1 | PchE |
| pyochelin | NRPS | pyochelin_C02_cyclization | cyclization | AAC83657 | BGC0000412 | 10555976 | *Pseudomonas aeruginosa* | PAO1 | PchF |
| pyoverdine | NRPS | pyoverdine_C02_DCL | DCL | AAX16297 | BGC0000413 | 15743962 | *Pseudomonas aeruginosa* | str 10-15 | PvdI |
| pyoverdine | NRPS | pyoverdine_C04_DCL | DCL | AAX16297 | BGC0000413 | 15743962 | *Pseudomonas aeruginosa* | str 10-15 | PvdI |
| pyoverdine | NRPS | pyoverdine_C01_LCL | LCL | AAX16297 | BGC0000413 | 15743962 | *Pseudomonas aeruginosa* | str 10-15 | PvdI |
| pyoverdine | NRPS | pyoverdine_C03_LCL | LCL | AAX16297 | BGC0000413 | 15743962 | *Pseudomonas aeruginosa* | str 10-15 | PvdI |
| pyoverdine | NRPS | pyoverdine_C05_LCL | LCL | AAX16296 | BGC0000413 | 15743962 | *Pseudomonas aeruginosa* | str 10-15 | PvdJ |
| pyoverdine | NRPS | pyoverdine_C06_LCL | LCL | AAX16296 | BGC0000413 | 15743962 | *Pseudomonas aeruginosa* | str 10-15 | PvdJ |
| pyoverdine | NRPS | pyoverdine_C07_LCL | LCL | AAX16295 | BGC0000413 | 15743962 | *Pseudomonas aeruginosa* | str 10-15 | PvdD |
| pyoverdine | NRPS | pyoverdine_C08_LCL | LCL | AAX16295 | BGC0000413 | 15743962 | *Pseudomonas aeruginosa* | str 10-15 | PvdD |
| pyoverdine | NRPS | pyoverdine_C02_DCL | DCL | AAX16297 | BGC0000413 | 15743962 | *Pseudomonas aeruginosa* | str 10-15 | PvdI |
| pyridomycin | PKS-NRPS | pyridomycin_C02_LCL | LCL | AEF33078 | BGC0001039 | 21454714 | *Streptomyces pyridomyceticus* | NRRL B-2517 | PyrE |
| pyridomycin | PKS-NRPS | pyridomycin_C03_LCL | LCL | AEF33080 | BGC0001039 | 21454714 | *Streptomyces pyridomyceticus* | NRRL B-2517 | PyrG |
| pyridomycin | PKS-NRPS | pyridomycin_C01_starter | starter | AEF33078 | BGC0001039 | 21454714 | *Streptomyces pyridomyceticus* | NRRL B-2517 | PyrE |
| salinosporamide | PKS-NRPS | salinosporamide_C01_LCL | LCL | ABP53498 | BGC0001041 | 19590008 | *Salinispora tropica* | CNB-440 | SalA |

| | | | | | | | | | |
|---|---|---|---|---|---|---|---|---|---|
| sporolide | PKS-NRPS | sporolide_C01_LCL | LCL | ABP55165 | BGC0000150 | 18232689 | *Salinispora tropica* | CNB-440 | SpoT10 |
| streptolydigin | PKS-NRPS | streptolydigin_C01_LCL | LCL | CBA11557 | BGC0001046 | 19875077 | *Streptomyces lydicus* | NRRL 2433 | SlgN2 |
| surfactin | NRPS | surfactin_C05_DCL | DCL | CAA49817 | BGC0000433 | 8355609 | *Bacillus subtilis* | W168 | SrfAB |
| surfactin | NRPS | surfactin_C09_DCL | DCL | CAA49818 | BGC0000433 | 8355609 | *Bacillus subtilis* | W168 | SrfAC |
| surfactin | NRPS | surfactin_C04_epimerization | epimerization | CAA49816 | BGC0000433 | 8355609 | *Bacillus subtilis* | W168 | SrfA1 |
| surfactin | NRPS | surfactin_C08_epimerization | epimerization | CAA49817 | BGC0000433 | 8355609 | *Bacillus subtilis* | W168 | SrfAB |
| surfactin | NRPS | surfactin_C02_LCL | LCL | CAA49816 | BGC0000433 | 8355609 | *Bacillus subtilis* | W168 | SrfA1 |
| surfactin | NRPS | surfactin_C03_LCL | LCL | CAA49816 | BGC0000433 | 8355609 | *Bacillus subtilis* | W168 | SrfA1 |
| surfactin | NRPS | surfactin_C06_LCL | LCL | CAA49817 | BGC0000433 | 8355609 | *Bacillus subtilis* | W168 | SrfAB |
| surfactin | NRPS | surfactin_C07_LCL | LCL | CAA49817 | BGC0000433 | 8355609 | *Bacillus subtilis* | W168 | SrfAB |
| surfactin | NRPS | surfactin_C01_starter | starter | CAA49816 | BGC0000433 | 8355609 | *Bacillus subtilis* | W168 | SrfA1 |
| syringomycin | NRPS | syringomycin_C02_dual | dual | AAC80285 | BGC0000437 | 9830033 | *Pseudomonas syringae pv. syringae* | | SyrE |
| syringomycin | NRPS | syringomycin_C03_dual | dual | AAC80285 | BGC0000437 | 9830033 | *Pseudomonas syringae pv. syringae* | | SyrE |
| syringomycin | NRPS | syringomycin_C04_dual | dual | AAC80285 | BGC0000437 | 9830033 | *Pseudomonas syringae pv. syringae* | | SyrE |
| syringomycin | NRPS | syringomycin_C08_dual | dual | AAC80285 | BGC0000437 | 9830033 | *Pseudomonas syringae pv. syringae* | | SyrE |
| syringomycin | NRPS | syringomycin_C05_LCL | LCL | AAC80285 | BGC0000437 | 9830033 | *Pseudomonas syringae pv. syringae* | | SyrE |
| syringomycin | NRPS | syringomycin_C06_LCL | LCL | AAC80285 | BGC0000437 | 9830033 | *Pseudomonas syringae pv. syringae* | | SyrE |
| syringomycin | NRPS | syringomycin_C07_LCL | LCL | AAC80285 | BGC0000437 | 9830033 | *Pseudomonas syringae pv. syringae* | | SyrE |
| syringomycin | NRPS | syringomycin_C09_LCL | LCL | AAC80285 | BGC0000437 | 9830033 | *Pseudomonas syringae pv. syringae* | | SyrE |
| syringomycin | NRPS | syringomycin_C01_starter | starter | AAC80285 | BGC0000437 | 9830033 | *Pseudomonas syringae pv. syringae* | | SyrE |
| teicoplanin | NRPS | teicoplanin_C01_DCL | DCL | CAG15009 | BGC0000441 | 15113000 | *Actinoplanes teichomyceticus* | | TeiA |
| teicoplanin | NRPS | teicoplanin_C02_DCL | DCL | CAG15010 | BGC0000441 | 15113000 | *Actinoplanes teichomyceticus* | | TeiB |
| teicoplanin | NRPS | teicoplanin_C03_DCL | DCL | CAG15011 | BGC0000441 | 15113000 | *Actinoplanes teichomyceticus* | | TeiC |
| teicoplanin | NRPS | teicoplanin_C04_DCL | DCL | CAG15011 | BGC0000441 | 15113000 | *Actinoplanes teichomyceticus* | | TeiC |
| teicoplanin | NRPS | teicoplanin_C05_DCL | DCL | CAG15011 | BGC0000441 | 15113000 | *Actinoplanes teichomyceticus* | | TeiC |
| teicoplanin | NRPS | teicoplanin_C06_DCL | DCL | CAG15012 | BGC0000441 | 15113000 | *Actinoplanes teichomyceticus* | | TeiD |
| thaxtomin | NRPS | thaxtomin_C01_LCL | LCL | AAG27087 | BGC0000444 | 11115114 | *Streptomyces acidiscabies* | | TxtA |
| thaxtomin | NRPS | thaxtomin_C02_LCL | LCL | AAG27088 | BGC0000444 | 11115114 | *Streptomyces acidiscabies* | | TxtB |
| thiocoraline | NRPS | thiocoraline_C03_DCL | DCL | CAJ34374 | BGC0000445 | 16408310 | *Micromonospora sp.* | ML-1 | TioR |
| thiocoraline | NRPS | thiocoraline_C02_epimerization | epimerization | CAJ34374 | BGC0000445 | 16408310 | *Micromonospora sp.* | ML-1 | TioR |
| thiocoraline | NRPS | thiocoraline_C04_LCL | LCL | CAJ34375 | BGC0000445 | 16408310 | *Micromonospora sp.* | ML-1 | TioS |
| thiocoraline | NRPS | thiocoraline_C05_LCL | LCL | CAJ34375 | BGC0000445 | 16408310 | *Micromonospora sp.* | ML-1 | TioS |
| thiocoraline | NRPS | thiocoraline_C01_starter | starter | CAJ34374 | BGC0000445 | 16408310 | *Micromonospora sp.* | ML-1 | TioR |
| tubulysin | PKS-NRPS | tubulysin_C04_cyclization | cyclization | CAF05649 | BGC0001053 | 15324808 | *Angiococcus disciformis* | And18 | TubD |
| tubulysin | PKS-NRPS | tubulysin_C01_LCL | LCL | CAF05647 | BGC0001053 | 15324808 | *Angiococcus disciformis* | And18 | TubB |
| tubulysin | PKS-NRPS | tubulysin_C02_LCL | LCL | CAF05648 | BGC0001053 | 15324808 | *Angiococcus disciformis* | And18 | TubC |
| tubulysin | PKS-NRPS | tubulysin_C03_LCL | LCL | CAF05648 | BGC0001053 | 15324808 | *Angiococcus disciformis* | And18 | TubC |
| tubulysin | PKS-NRPS | tubulysin_C05_LCL | LCL | CAF05650 | BGC0001053 | 15324808 | *Angiococcus disciformis* | And18 | TubE |
| tyrocidine | NRPS | tyrocidine_C02_DCL | DCL | AAC45929 | BGC0000452 | 9352938 | *Brevibacillus brevis* | ATCC 8185 | TycB |

| | | | | | | | | | |
|---|---|---|---|---|---|---|---|---|---|
| tyrocidine | NRPS | tyrocidine_C06_DCL | DCL | AAC45930 | BGC0000452 | 9352938 | *Brevibacillus brevis* | ATCC 8185 | TycC |
| tyrocidine | NRPS | tyrocidine_C01_epimerization | epimerization | AAC45928 | BGC0000452 | 9352938 | *Brevibacillus brevis* | ATCC 8185 | TycA |
| tyrocidine | NRPS | tyrocidine_C05_epimerization | epimerization | AAC45929 | BGC0000452 | 9352938 | *Brevibacillus brevis* | ATCC 8185 | TycB |
| tyrocidine | NRPS | tyrocidine_C03_LCL | LCL | AAC45929 | BGC0000452 | 9352938 | *Brevibacillus brevis* | ATCC 8185 | TycB |
| tyrocidine | NRPS | tyrocidine_C04_LCL | LCL | AAC45929 | BGC0000452 | 9352938 | *Brevibacillus brevis* | ATCC 8185 | TycB |
| tyrocidine | NRPS | tyrocidine_C07_LCL | LCL | AAC45930 | BGC0000452 | 9352938 | *Brevibacillus brevis* | ATCC 8185 | TycC |
| tyrocidine | NRPS | tyrocidine_C08_LCL | LCL | AAC45930 | BGC0000452 | 9352938 | *Brevibacillus brevis* | ATCC 8185 | TycC |
| tyrocidine | NRPS | tyrocidine_C09_LCL | LCL | AAC45930 | BGC0000452 | 9352938 | *Brevibacillus brevis* | ATCC 8185 | TycC |
| tyrocidine | NRPS | tyrocidine_C10_LCL | LCL | AAC45930 | BGC0000452 | 9352938 | *Brevibacillus brevis* | ATCC 8185 | TycC |
| tyrocidine | NRPS | tyrocidine_C11_LCL | LCL | AAC45930 | BGC0000452 | 9352938 | *Brevibacillus brevis* | ATCC 8185 | TycC |
| vibriobactin | NRPS | vibriobactin_C01_starter | starter | AAF93940 | na | 12040125 | *Vibrio cholerae* | N16961 | VC0775 |
| viomycin | NRPS | viomycin_C01_DCL | DCL | AAP92496 | BGC0000458 | 12936980 | *Streptomyces vinaceus* | ATCC 11861 | VioF |
| viomycin | NRPS | viomycin_C02_LCL | LCL | AAP92491 | BGC0000458 | 12936980 | *Streptomyces vinaceus* | ATCC 11861 | VioA |
| viomycin | NRPS | viomycin_C03_LCL | LCL | AAP92491 | BGC0000458 | 12936980 | *Streptomyces vinaceus* | ATCC 11861 | VioA |
| viomycin | NRPS | viomycin_C04_LCL | LCL | AAP92499 | BGC0000458 | 12936980 | *Streptomyces vinaceus* | ATCC 11861 | VioI |
| viomycin | NRPS | viomycin_C05_LCL | LCL | AAP92503 | BGC0000458 | 12936980 | *Streptomyces vinaceus* | ATCC 11861 | VioM |
| yersiniabactin | PKS-NRPS | yersiniabactin_C01_cyclization | cyclization | AAC69587 | BGC0000467 | 9818149 | *Yersinia pestis* | KIM6 | Irp1 |
| yersiniabactin | PKS-NRPS | yersiniabactin_C02_cyclization | cyclization | AAC69587 | BGC0000467 | 9818149 | *Yersinia pestis* | KIM6 | Irp1 |
| yersiniabactin | PKS-NRPS | yersiniabactin_C03_cyclization | cyclization | AAC69588 | BGC0000467 | 9818149 | *Yersinia pestis* | KIM6 | Irp2 |

**Table S11.** Minimal inhibitory concentration (MIC) values for chitinimines I/III and II against a range of Gram-positive bacteria.

| Strain | Minimal Inhibitory Concentration (µg/mL) | |
|---|---|---|
| | **Chitinimine I/III** | **Chitinimine II** |
| *Enterococcus faecium* DSM 25390 | 64 | 256 |
| *Bacillus cereus* DSM 31 | 32 | 64 |
| *Bacillus subtilis* ATCC 9799 | 64 | 128 |
| *Staphylococcus aureus* DSM 21979 | 128 | 256 |
| *Staphylococcus aureus* RN4220 | >256 | 128 |
| *Staphylococcus aureus* ATCC 6538 | 256 | 256 |
| *Staphylococcus aureus* StaAu068 | 256 | >256 |
| *Staphylococcus aureus* Sa9 | 256 | 128 |
| *Staphylococcus capitis* StaCa010 | 128 | 256 |
| *Staphylococcus haemolyticus* StaHa024 | 128 | 256 |
| *Staphylococcus hominis* StaHo017 | 256 | 256 |
| *Staphylococcus lugdunensis* StaLu018 | 128 | 256 |
| *Mycobacterium smegmatis* MC2-155 | 128 | >256 |

**Table S12.** KS proteins encoded in *pfaA* homologs used for phylogenetic analysis of the genome mining results.

| Species | Strain | Protein ID (NCBI) |
|---|---|---|
| *Shewanella pealeana* | ATCC 700345 | WP_012156130.1 |
| *Microcystis aeruginosa* | NIES-843 | WP_012265837.1 |
| *Microcystis aeruginosa* | str. Chao 1910 | WP_190357376.1 |
| *Nostoc* sp. | PCC 7524 / ATCC 29411 | WP_015137690.1 |
| *Trichormus variabilis* | NIES-23 | WP_096637125.1 |
| *Nostoc commune* | NIES-4072 | WP_109009590.1 |
| *Nostoc flagelliforme* | CCNUN1 | WP_100903912.1 |
| *Nostoc sphaeroides* | CCNUC1 | WP_152591243.1 |
| *Anabaena* sp. | YBS01 | WP_011321408.1 |
| *Scytonema hofmannii* | PCC 7110 | WP_051077101.1 |
| *Collimonas arenae* | Cal35 | WP_038484514.1 |
| *Chromobacterium sp.* | ATCC 53434 | WP_158300868.1 |
| *Paraburkholderia megapolitana* | LMG 23650 | WP_091015040.1 |
| *Chitinimonas koreensis* | DSM 17726 | WP_084300472.1 |
| *Ottowia thiooxydans* | DSM 14619 | WP_051237177.1 |
| *Tistlia consotensis* | USBA 355 | WP_085121617.1 |
| *Tahibacter aquaticus* | DSM 21667 | WP_133819585.1 |
| *Aquimarina* sp. | TRL1 | WP_176027148.1 |
| *Flavobacterium* sp. | N502540 | WP_264530534.1 |
| *Flavobacterium* sp. | F-323 | WP_230002366.1 |
| *Chitinivorax tropics* | DSM 27165 | WP_184033482.1 |
| *Paludibacterium paludis* | BCRC 80514 | WP_189533434.1 |
| *Bowmanella denitrificans* | JL63 | WP_102796139.1 |
| *Streptomyces griseus* | JCM 4516 | WP_193463672.1 |
| *Streptomyces* sp. | 43Y-GA-1 | WP_249627416.1 |
| *Streptomyces baarnensis* | NRRL B-2842 | WP_030081506.1 |
| *Streptomyces* sp. | WY228 | WP_218784432.1 |
| *Mycolicibacterium* sp. | CBMA 234 | WP_155924955.1 |
| *Mycobacterium fortunisiensis* | TNTM28 | WP_246584842.1 |
| *Mycobacterium simulans* | FB-527 | WP_260860972.1 |
| *Serratia* sp. | AS12 | WP_013814526.1 |
| *Serratia plymuthica* | S13 | WP_020439798.1 |
| | RVH1 | WP_006328030.1 |
| | Isolate 68f6912a-a76c-11e8-a962-3c4a9275d6c8 | WP_166728837.1 |
| | V4 | WP_208904410.1 |
| | 3Rp8 | WP_064800005.1 |
| | 3Re4-18 | WP_006328030.1 |
| | IV-11-34 | WP_166728837.1 |
| | MBSA-MJ1 | WP_202291812.1 |
| | A294 | WP_166728837.1 |
| | C-1 | WP_252978576.1 |
| | FDAARGOS_907 | WP_232246046.1 |
| | FDAARGOS_889 | WP_197912311.1 |
| | FDAARGOS_896 | WP_232246922.1 |
| | B37/06 | WP_241922251.1 |
| *Dickeya solani* | GBBC 2040 | WP_223849466.1 |
| | RNS 05.1.2A | WP_057083446.1 |
| *Dickeya* sp. | NCPPB 3274 | WP_238556095.1 |
| | Secpp 1600 | WP_255412271.1 |
| *Dickeya zeae* | EC1 | WP_237712637.1 |

| | | |
|---|---|---|
| *Dickeya fangzhongdai* | ND14b | WP_240476029.1 |
| | PA1 | WP_236883942.1 |
| | DSM 101947 | WP_225623144.1 |
| | ZXC1 | WP_276196108.1 |
| | 908C | WP_245167343.1 |
| | S1 | WP_242449541.1 |
| | B16 | WP_231348849.1 |
| | 643b | WP_239788825.1 |
| | AP6 | WP_241043386.1 |
| *Dickeya dadantii* | A622-S1-A17 | WP_226055486.1 |
| | S3-1 | WP_245000879.1 |
| | FZ06 | WP_263065003.1 |
| *Dickeya oryzae* | ZYY5 | WP_268906959.1 |
| *Xenorhabdus hominickii* | ANU1 | WP_084022942.1 |
| *Xenorhabdus budapestensis* | C-7-2 | WP_209028459.1 |
| *Xenorhabdus innexi* | HGB1681 | WP_086953270.1 |
| *Xenorhabdus szentirmaii* | DSM 16338 | WP_084616193.1 |

**Table S13.** KS-CLF heterodimers encoded in *pfaC* homologs used for phylogenetic analyses of the genome mining results.

| Species | Strain | Protein ID (NCBI) |
|---|---|---|
| *Shewanella pealeana* | ATCC 700345 | WP_012156132.1 |
| *Nostoc* sp. | PCC 7524 / ATCC 29411 | WP_015137694.1 |
| *Collimonas arenae* | Cal35 | WP_038484511.1 |
| *Chromobacterium sp.* | ATCC 53434 | WP_158300869.1 |
| *Paraburkholderia megapolitana* | LMG 23650 | WP_091015038.1 |
| *Chitinimonas koreensis* | DSM 17726 | WP_028447101.1 |
| *Tistlia consotensis* | USBA 355 | WP_085121616.1 |
| *Tahibacter aquaticus* | DSM 21667 | WP_133819586.1 |
| *Chitinivorax tropics* | DSM 27165 | WP_184033479.1 |
| *Paludibacterium paludis* | BCRC 80514 | WP_189533432.1 |
| *Bowmanella denitrificans* | JL63 | WP_155924956.1 |
| *Streptomyces griseus* | JCM 4516 | WP_193463673.1 |
| *Streptomyces* sp. | 43Y-GA-1 | WP_249627417.1 |
| *Streptomyces baarnensis* | NRRL B-2842 | WP_030081504.1 |
| *Streptomyces* sp. | WY228 | WP_218784434.1 |
| *Mycolicibacterium* sp. | CBMA 234 | WP_155924956.1 |
| *Mycobacterium fortunisiensis* | TNTM28 | WP_246584843.1 |
| *Mycobacterium simulans* | FB-527 | WP_186241947.1 |
| *Serratia* sp. | AS12 | WP_013814525.1 |
| *Serratia plymuthica* | S13 | WP_020439797.1 |
| | RVH1 | WP_006328028.1 |
| | Isolate 68f6912a-a76c-11e8-a962-3c4a9275d6c8 | WP_006328028.1 |
| | V4 | WP_208904409.1 |
| | 3Rp8 | WP_006328028.1 |
| | 3Re4-18 | WP_064799185.1 |
| | IV-11-34 | WP_006328028.1 |
| | MBSA-MJ1 | WP_197912312.1 |
| | A294 | WP_006328028.1 |

| | | |
|---|---|---|
| | C-1 | WP_252978577.1 |
| | FDAARGOS_907 | WP_197913209.1 |
| | FDAARGOS_889 | WP_197912312.1 |
| | FDAARGOS_896 | WP_197929578.1 |
| | B37/06 | WP_241921682.1 |
| *Dickeya solani* | GBBC 2040 | WP_022632850.1 |
| | RNS 05.1.2A | WP_057083445.1 |
| *Dickeya* sp. | NCPPB 3274 | WP_042859297.1 |
| | Secpp 1600 | WP_107758962.1 |
| *Dickeya zeae* | EC1 | WP_016943533.1 |
| *Dickeya fangzhongdai* | ND14b | WP_038660417.1 |
| | PA1 | WP_121479904.1 |
| | DSM 101947 | WP_100849226.1 |
| | ZXC1 | WP_276196109.1 |
| | 908C | WP_209126573.1 |
| | S1 | WP_049854996.1 |
| | B16 | WP_038918429.1 |
| | 643b | WP_239788826.1 |
| | AP6 | WP_161131253.1 |
| *Dickeya dadantii* | A622-S1-A17 | WP_226055485.1 |
| | S3-1 | WP_216282268.1 |
| | FZ06 | WP_263065004.1 |
| *Dickeya oryzae* | ZYY5 | WP_016943533.1 |
| *Xenorhabdus hominickii* | ANU1 | WP_069315170.1 |
| *Xenorhabdus budapestensis* | C-7-2 | WP_209028460.1 |
| *Xenorhabdus innexi* | HGB1681 | WP_086953269.1 |
| *Xenorhabdus szentirmaii* | DSM 16338 | WP_038236910.1 |

**Table S14. Bacterial and fungal strains tested for susceptibility to the chitinimines and their culture conditions.** LB = lysogeny broth, BHI = brain heart infusion broth, R2A = Reasoner's 2A medium, NA = nutrient agar, YPD = Yeast extract Peptone Dextrose.

| Species | Strain | Biosafety level | Growth medium | Temperature |
|---|---|---|---|---|
| **Gram-positive bacteria** | | | | |
| *Enterococcus faecium* | DSM 25390 | 2 | LB | 37°C |
| *Staphylococcus aureus* | DSM 21979 | 2 | LB | 37°C |
| *Staphylococcus aureus* | RN4220 | 2 | LB | 37°C |
| *Staphylococcus aureus* | ATCC 6538 | 2 | LB | 37°C |
| *Staphylococcus aureus* | StaAu068 | 2 | LB | 37°C |
| *Staphylococcus aureus* | Sa9 | 2 | LB | 37°C |
| *Staphylococcus capitis* | StaCa010 | 2 | LB | 37°C |
| *Staphylococcus epidermidis* | StaEp012 | 2 | LB | 37°C |
| *Staphylococcus haemolyticus* | StaHa024 | 2 | LB | 37°C |
| *Staphylococcus hominis* | StaHo017 | 2 | LB | 37°C |
| *Staphylococcus lugdunensis* | StaLu018 | 2 | LB | 37°C |
| *Bacillus cereus* | DSM 31/ATCC 14579 | 2 | LB | 30°C |
| *Bacillus subtilis* | ATCC 9799 | 1 | LB | 30°C |
| *Listeria monocytogenes* | LMH7738 | 2 | BHI | 30°C |
| *Gordonia bronchialis* | DSM 43247 | 2 | R2A | 28°C |
| *Mycobacterium smegmatis* | MC2-155 | 1 | LB | 37°C |
| **Gram-negative bacteria** | | | | |
| *Acinetobacter baumannii* | DSM 25645 | 2 | LB | 37°C |
| *Enterobacter roggenkampii* | DSM 16690 | 2 | LB | 37°C |
| *Klebsiella pneumoniae* | DSM 103517 | 2 | LB | 28°C |
| *Burkholderia multivorans* | 11/0583 | 2 | LB | 30°C |
| *Burkholderia singularis* | LMG 28154 | 2 | LB | 28°C |
| *Salmonella enterica* serovar Newport | C487 | 2 | LB | 37°C |
| *Salmonella enterica* serovar Typhimurium | ATCC 14028 (+GFP) | 2 | LB | 37°C |
| *Salmonella enterica* serovar Typhimurium | LT2 | 2 | LB | 37°C |
| *Salmonella enterica* serovar Enteritidis | ATCC 13046 | 2 | LB | 37°C |
| *Salmonella enterica* serovar Heidelberg | #10 | 2 | LB | 37°C |
| *Caballeronia udeis* | LMG 27134 | 1 | R2A | 28°C |
| *Massilia sp. Root* 335 | DSM 102448 | 1 | R2A | 28°C |
| *Massilia flava* | DSM 26639 | 1 | R2A | 28°C |
| *Paraburkholderia megapolitana* | LMG 23650 | 1 | R2A | 28°C |
| *Trinickia dinghuensis* | LMG30259 | 1 | R2A | 28°C |
| *Robbsia andropogonis* | DSM 9511 | 1 | NA | 28°C |
| **Fungi** | | | | |
| *Candida albicans* | SC5314 | 2 | YPD | 30°C |
| *Candida albicans* | DPL1007 | 2 | YPD | 30°C |
| *Candida albicans* | DSY296 | 2 | YPD | 30°C |
| *Candida auris Clade I* | MDR OS299 | 2 | YPD | 37°C |
| *Candida auris Clade I* | *reference strain* B8441 | 2 | YPD | 37°C |
| *Candida auris Clade II* | *reference strain* B11220 | 2 | YPD | 37°C |
| *Candida. glabrata* | ATCC 2001 | 2 | YPD | 37°C |

## References


[1] Kim, B.-Y.; Weon, H.-Y.; Yoo, S.-H.; Chen, W.-M.; Kwon, S.-W.; Go, S.-J.; Stackebrandt, E. Chitinimonas koreensis sp. nov., Isolated from Greenhouse Soil in Korea. *Int. J. Syst. Evol. Microbiol.* **2006**, *56* (Pt 8), 1761–1764.

[2] Miller, V. L.; Mekalanos, J. J. A Novel Suicide Vector and Its Use in Construction of Insertion Mutations: Osmoregulation of Outer Membrane Proteins and Virulence Determinants in *Vibrio cholerae* Requires toxR. *J. Bacteriol.* **1988**, *170* (6), 2575–2583.

[3] López, C. M.; Rholl, D. A.; Trunck, L. A.; Schweizer, H. P. Versatile Dual-Technology System for Markerless Allele Replacement in *Burkholderia pseudomallei*. *Appl. Environ. Microbiol.* **2009**, *75* (20), 6496–6503.

[4] Rubirés, X.; Saigi, F.; Piqué, N.; Climent, N.; Merino, S.; Albertí, S.; Tomás, J. M.; Regué, M. A Gene (*wbbL*) from *Serratia marcescens* N28b (O4) Complements the rfb-50 Mutation of *Escherichia coli* K-12 Derivatives. *J. Bacteriol.* **1997**, *179* (23), 7581–7586.

[5] Blin, K.; Shaw, S.; Augustijn, H. E.; Reitz, Z. L.; Biermann, F.; Alanjary, M.; Fetter, A.; Terlouw, B. R.; Metcalf, W. W.; Helfrich, E. J. N.; van Wezel, G. P.; Medema, M. H.; Weber, T. antiSMASH 7.0: New and Improved Predictions for Detection, Regulation, Chemical Structures, and Visualization. *Nucleic Acids Res.* **2023**, *51* (W1), W46–W50.

[6] Blin, K.; Shaw, S.; Medema, M. H.; Weber, T. The antiSMASH Database Version 4: Additional Genomes and BGCs, New Sequence-Based Searches and More. *Nucleic Acids Res.* **2024**, *52 (*D1), D586–D589.

[7] Zdouc, M. M.; Blin, K.; Louwen, N. L. L.; Navarro, J.; Loureiro, C.; Bader, C. D.; Bailey, C. B.; Barra, L.; Booth, T. J.; Bozhüyük, K. A. J.; Cediel-Becerra, J. D. D.; Charlop-Powers, Z.; Chevrette, M. G.; Chooi, Y. H.; D'Agostino, P. M.; de Rond, T.; Del Pup, E.; Duncan, K. R.; Gu, W.; Hanif, N.; Helfrich, E. J. N.; Jenner, M.; Katsuyama, Y.; Korenskaia, A.; Krug, D.; Libis, V.; Lund, G. A.; Mantri, S.; Morgan, K. D.; Owen, C.; Phan, C.-S.; Philmus, B.; Reitz, Z. L.; Robinson, S. L.; Singh, K. S.; Teufel, R.; Tong, Y.; Tugizimana, F.; Ulanova, D.; Winter, J. M.; Aguilar, C.; Akiyama, D. Y.; Al-Salihi, S. A. A.; Alanjary, M.; Alberti, F.; Aleti, G.; Alharthi, S. A.; Rojo, M. Y. A.; Arishi, A. A.; Augustijn, H. E.; Avalon, N. E.; Avelar-Rivas, J. A.; Axt, K. K.; Barbieri, H. B.; Barbosa, J. C. J.; Barboza Segato, L. G.; Barrett, S. E.; Baunach, M.; Beemelmanns, C.; Beqaj, D.; Berger, T.; Bernaldo-Agüero, J.; Bettenbühl, S. M.; Bielinski, V. A.; Biermann, F.; Borges, R. M.; Borriss, R.; Breitenbach, M.; Bretscher, K. M.; Brigham, M. W.; Buedenbender, L.; Bulcock, B. W.; Cano-Prieto, J.; Capela, J.; Carrion, V. J.; Carter, R. S.; Castelo-Branco, R.; Castro-Falcón, G.; Chagas, F. O.; Charria-Girón, E.; Chaudhri, A. A.; Chaudhry, V.; Choi, H.; Choi, Y.; Choupannejad, R.; Chromy, J.; Donahey, M. S. C.; Collemare, J.; Connolly, J. A.; Creamer, K. E.; Crüsemann, M.; Cruz, A. A.; Cumsille, A.; Dallery, J.-F.; Damas-Ramos, L. C.; Damiani, T.; de Kruijff, M.; Martín, B. D.; Sala, G. D.; Dillen, J.; Doering, D. T.; Dommaraju, S. R.; Durusu, S.; Egbert, S.; Ellerhorst, M.; Faussurier, B.; Fetter, A.; Feuermann, M.; Fewer, D. P.; Foldi, J.; Frediansyah, A.; Garza, E. A.; Gavriilidou, A.; Gentile, A.; Gerke, J.; Gerstmans, H.; Gomez-Escribano, J. P.; González-Salazar, L. A.; Grayson, N. E.; Greco, C.; Gomez, J. E. G.; Guerra, S.; Flores, S. G.; Gurevich, A.; Gutiérrez-García, K.; Hart, L.; Haslinger, K.; He, B.; Hebra, T.; Hemmann, J. L.; Hindra; Höing, L.; Holland, D. C.; Holme, J. E.; Horch, T.; Hrab, P.; Hu, J.; Huynh, T.-H.; Hwang, J.-Y.; Iacovelli, R.; Iftime, D.; Iorio, M.; Jayachandran, S.; Jeong, E.; Jing, J.; Jung, J. J.; Kakumu, Y.; Kalkreuter, E.; Kang, K. B.; Kang, S.; Kim, W.; Kim, G. J.; Kim, H.; Kim, H. U.; Klapper, M.; Koetsier, R. A.; Kollten, C.; Kovács, Á. T.; Kriukova, Y.; Kubach, N.; Kunjapur, A. M.; Kushnareva, A. K.; Kust, A.; Lamber, J.; Larralde, M.; Larsen, N. J.; Launay, A. P.; Le, N.-T.-H.; Lebeer, S.; Lee, B. T.; Lee, K.; Lev, K. L.; Li, S.-M.; Li, Y.-X.; Licona-Cassani, C.; Lien, A.; Liu, J.; Lopez, J. A. V.; Machushynets, N. V.; Macias, M. I.; Mahmud, T.; Maleckis, M.; Martinez-Martinez, A. M.; Mast, Y.; Maximo, M. F.; McBride, C. M.; McLellan, R. M.; Bhatt, K. M.; Melkonian, C.; Merrild, A.; Metsä-Ketelä, M.; Mitchell, D. A.; Müller, A. V.; Nguyen, G.-S.; Nguyen, H. T.; Niedermeyer, T. H. J.; O'Hare, J. H.; Ossowicki, A.; Ostash, B. O.; Otani, H.; Padva, L.; Paliyal, S.; Pan, X.; Panghal, M.; Parade, D. S.; Park, J.; Parra, J.; Rubio, M. P.; Pham, H. T.; Pidot, S. J.; Piel, J.; Pourmohsenin, B.; Rakhmanov, M.; Ramesh, S.; Rasmussen, M. H.; Rego, A.; Reher, R.; Rice, A. J.; Rigolet, A.; Romero-Otero, A.; Rosas-Becerra, L. R.; Rosiles, P. Y.; Rutz, A.; Ryu, B.; Sahadeo, L.-A.; Saldanha, M.; Salvi, L.; Sánchez-Carvajal, E.; Santos-Medellin, C.; Sbaraini, N.; Schoellhorn, S. M.; Schumm, C.; Sehnal, L.; Selem, N.; Shah, A. D.; Shishido, T. K.; Sieber, S.; Silviani, V.; Singh, G.; Singh, H.; Sokolova, N.; Sonnenschein, E. C.; Sosio, M.; Sowa, S. T.; Steffen, K.; Stegmann, E.; Streiff, A. B.; Strüder, A.; Surup, F.; Svenningsen, T.; Sweeney, D.; Szenei, J.; Tagirdzhanov, A.; Tan, B.; Tarnowski, M. J.; Terlouw, B. R.; Rey, T.; Thome, N. U.; Torres Ortega, L. R.; Tørring, T.; Trindade, M.; Truman, A. W.; Tvilum, M.; Udwary, D. W.; Ulbricht, C.; Vader, L.; van Wezel, G. P.; Walmsley, M.; Warnasinghe, R.; Weddeling, H. G.; Weir, A. N. M.; Williams, K.; Williams, S. E.; Witte, T. E.; Rocca, S. M. W.; Yamada, K.; Yang, D.; Yang, D.; Yu, J.; Zhou, Z.; Ziemert, N.; Zimmer, L.; Zimmermann, A.; Zimmermann, C.; van der Hooft, J. J. J.; Linington, R. G.; Weber, T.; Medema, M. H. MIBiG 4.0: Advancing Biosynthetic Gene Cluster Curation through Global Collaboration. *Nucleic Acids Res.* **2025**, *53* (D1), D678–D690.

[8] Trifinopoulos, J.; Nguyen, L.-T.; von Haeseler, A.; Minh, B. Q. W-IQ-TREE: A Fast Online Phylogenetic Tool for Maximum Likelihood Analysis. *Nucleic Acids Res.* **2016**, *44* (W1), W232–W235.

[9] Letunic, I.; Bork, P. Interactive Tree of Life (iTOL) v6: Recent Updates to the Phylogenetic Tree Display and Annotation Tool. *Nucleic Acids Res.* **2024**, *52* (W1), W78–W82.
[10] Navarro-Muñoz, J. C.; Selem-Mojica, N.; Mullowney, M. W.; Kautsar, S. A.; Tryon, J. H.; Parkinson, E. I.; De Los Santos, E. L. C.; Yeong, M.; Cruz-Morales, P.; Abubucker, S.; Roeters, A.; Lokhorst, W.; Fernandez-Guerra, A.; Cappelini, L. T. D.; Goering, A. W.; Thomson, R. J.; Metcalf, W. W.; Kelleher, N. L.; Barona-Gomez, F.; Medema, M. H. A Computational Framework to Explore Large-Scale Biosynthetic Diversity. *Nat. Chem. Biol.* **2020**, *16* (1), 60–68.
[11] Gilchrist, C. L. M.; Chooi, Y.-H. clinker & clustermap.js: Automatic Generation of Gene Cluster Comparison Figures. *Bioinformatics* **2021**, *37* (16), 2473–2475.
[12] Garcia, E. C. *Burkholderia thailandensis*: Genetic Manipulation. *Curr. Protoc. Microbiol.* **2017**, *45*, 4C.2.1–4C.2.15.
[13] Hareland, W. A.; Crawford, R. L.; Chapman, P. J.; Dagley, S. Metabolic Function and Properties of 4-Hydroxyphenylacetic Acid 1-Hydroxylase from *Pseudomonas acidovorans*. *J. Bacteriol.* **1975**, *121* (1), 272–285.
[14] Thrippleton, M. J.; Keeler, J. Elimination of Zero-Quantum Interference in Two-Dimensional NMR Spectra. *Angew. Chem. Int. Ed.* **2003**, *42* (33), 3938–3941.
[15] Shaka, A. J.; Lee, C. J.; Pines, A. Iterative Schemes for Bilinear Operators: Application to Spin Decoupling. *J. Magn. Reson.* **1988**, *77* (2), 274–293.
[16] Jeener, J.; Meier, B. H.; Bachmann, P.; Ernst, R. R. Investigation of Exchange Processes by Two-Dimensional NMR Spectroscopy. *J. Chem. Phys.* **1979**, *71*, 4546–4553.
[17] Schleucher, J.; Schwendinger, M.; Sattler, M.; Schmidt, P.; Schedletzky, O.; Glaser, S. J.; Sørensen, O. W.; Griesinger, C. A General Enhancement Scheme in Heteronuclear Multidimensional NMR Employing Pulsed Field Gradients. *J. Biomol. NMR* **1994**, *4* (2), 301–306.
[18] Griesinger, C.; Otting, G.; Wüthrich, K.; Ernst, R. R. Clean TOCSY for Proton Spin System Identification in Macromolecules. *J. Am. Chem. Soc.* **1988**, *110* (23), 7870–7872.
[19] Tanino, T.; Ichikawa, S.; Shiro, M.; Matsuda, A. Total Synthesis of (−)-Muraymycin D2 and Its Epimer. *J. Org. Chem.* **2010**, *75* (5), 1366–1377.
[20] Neese, F. Software Update: The ORCA Program System—Version 6.0. *WIREs Comput. Mol. Sci.* **2025**, *15* (2), e70019.
[21] Neese, F.; Wennmohs, F.; Becker, U.; Riplinger, C. The ORCA Quantum Chemistry Program Package. *J. Chem. Phys.* **2020**, *152* (22), 224108.
[22] Wittmann, L.; Gordiy, I.; Friede, M.; Helmich-Paris, B.; Grimme, S.; Hansen, A.; Bursch, M. Extension of the D3 and D4 London Dispersion Corrections to the Full Actinide Series. *Phys. Chem. Chem. Phys.* **2024**, *26* (32), 21379–21394.
[23] Neese, F. Software Update: The ORCA Program System—Version 5.0. *WIREs Comput. Mol. Sci.* **2022**, *12* (5), e1606.
[24] Neese, F. The SHARK Integral Generation and Digestion System. *J. Comput. Chem.* **2023**, *44* (3), 381–396.
[25] Helmich-Paris, B.; de Souza, B.; Neese, F.; Izsák, R. An Improved Chain-of-Spheres for Exchange Algorithm. *J. Chem. Phys.* **2021**, *155* (10), 104109.
[26] Caldeweyher, E.; Mewes, J.-M.; Ehlert, S.; Grimme, S. Extension and Evaluation of the D4 London Dispersion Model for Periodic Systems. *Phys. Chem. Chem. Phys.* **2020**, *22* (16), 8499–8512.
[27] Caldeweyher, E.; Bannwarth, C.; Grimme, S. Extension of the D3 Dispersion Coefficient Model. *J. Chem. Phys.* **2017**, *147* (3), 034112.
[28] Bykov, D.; Petrenko, T.; Izsák, R.; Kossmann, S.; Becker, U.; Valeev, E.; Neese, F. Efficient Implementation of the Analytic Second Derivatives of Hartree–Fock and Hybrid DFT Energies. *Mol. Phys.* **2015**, *113* (13-14), 1961–1977.
[29] Izsák, R.; Neese, F.; Klopper, W. Robust Fitting Techniques in the Chain-of-Spheres Approximation to the Fock Exchange. *J. Chem. Phys.* **2013**, *139* (9), 094111.
[30] Neese, F. The ORCA Program System. *WIREs Comput. Mol. Sci.* **2012**, *2* (1), 73–78.
[31] Izsák, R.; Neese, F. An Overlap-Fitted Chain-of-Spheres Exchange Method. *J. Chem. Phys.* **2011**, *135* (14), 144105.
[32] Neese, F.; Wennmohs, F.; Hansen, A.; Becker, U. Efficient, Approximate and Parallel Hartree–Fock and Hybrid DFT Calculations. *Chem. Phys.* **2009**, *356* (1-3), 98–109.
[33] Pracht, P.; Bohle, F.; Grimme, S. Automated Exploration of the Low-Energy Chemical Space with Fast Quantum Chemical Methods. *Phys. Chem. Chem. Phys.* **2020**, *22* (14), 7169–7192.
[34] Grimme, S.; Bohle, F.; Hansen, A.; Pracht, P.; Spicher, S.; Stahn, M. Efficient Quantum Chemical Calculation of Structure Ensembles and Free Energies for Nonrigid Molecules. *J. Phys. Chem. A* **2021**, *125* (19), 4039–4054.
[35] Bannwarth, C.; Caldeweyher, E.; Ehlert, S.; Hansen, A.; Pracht, P.; Seibert, J.; Spicher, S.; Grimme, S. Extended Tight-Binding Quantum Chemistry Methods. *WIREs Comput. Mol. Sci.* **2021**, *11* (2), e1493.
[36] Bannwarth, C.; Ehlert, S.; Grimme, S. GFN2-xTB-An Accurate and Broadly Parametrized Self-Consistent Tight-Binding Quantum Chemical Method. *J. Chem. Theory Comput.* **2019**, *15* (3), 1652–1671.
[37] Ehlert, S.; Stahn, M.; Spicher, S.; Grimme, S. Robust and Efficient Implicit Solvation Model for Fast Semiempirical Methods. *J. Chem. Theory Comput.* **2021**, *17* (7), 4250–4261.

[38] Caldeweyher, E.; Ehlert, S.; Hansen, A.; Neugebauer, H.; Spicher, S.; Bannwarth, C.; Grimme, S. A Generally Applicable Atomic-Charge-Dependent London Dispersion Correction. *J. Chem. Phys.* **2019**, *150* (15), 154122.

[39] Garcia-Ratés, M.; Neese, F. Effect of the Solute Cavity on the Solvation Energy and Its Derivatives within the Framework of the Gaussian Charge Scheme. *J. Comput. Chem.* **2020**, *41* (9), 922–939.

[40] Chemcraft - Graphical Software for Visualization of Quantum Chemistry Computations, Version 1.8, Build 682; https://www.chemcraftprog.com.

[41] Wiitala, K. W.; Hoye, T. R.; Cramer, C. J. Hybrid Density Functional Methods Empirically Optimized for the Computation of (13)C and (1)H Chemical Shifts in Chloroform Solution. *J. Chem. Theory Comput.* **2006**, *2* (4), 1085–1092.

[42] Hehre, W. J.; Ditchfield, R.; Pople, J. A. Self-Consistent Molecular Orbital Methods. XII. Further Extensions of Gaussian-Type Basis Sets for Use in Molecular Orbital Studies of Organic Molecules. *J. Chem. Phys.* **1972**, *56*, 2257–2261.

[43] Cohen, R. D.; Wood, J. S.; Lam, Y.-H.; Buevich, A. V.; Sherer, E. C.; Reibarkh, M.; Williamson, R. T.; Martin, G. E. DELTA50: A Highly Accurate Database of Experimental $^{1}$H and $^{13}$C NMR Chemical Shifts Applied to DFT Benchmarking. *Molecules* **2023**, *28* (6), 2449.

[44] Terlouw, B. R.; Huang, C.; Meijer, D.; Cediel-Becerra, J. D. D.; Rothe, M. L.; Jenner, M.; Zhou, S.; Zhang, Y.; Fage, C. D.; Tsunematsu, Y.; van Wezel, G. P.; Robinson, S. L.; Alberti, F.; Alkhalaf, L. M.; Chevrette, M. G.; Challis, G. L.; Medema, M. H. bioRxiv **2025**, DOI: 10.1101/2025.01.08.631717.

[45] Klau, L. J.; Podell, S.; Creamer, K. E.; Demko, A. M.; Singh, H. W.; Allen, E. E.; Moore, B. S.; Ziemert, N.; Letzel, A. C.; Jensen, P. R. The Natural Product Domain Seeker Version 2 (NaPDoS2) Webtool Relates Ketosynthase Phylogeny to Biosynthetic Function. *J. Biol. Chem.* **2022**, *298* (10), 102480.

[46] Wirth, N. T.; Funk, J.; Donati, S.; Nikel, P. I. QurvE: User-Friendly Software for the Analysis of Biological Growth and Fluorescence Data. *Nat. Protoc.* **2023**, *18* (8), 2401–2403.

[47] Clinical and Laboratory Standards Institute (CLSI). *Methods for Dilution Antimicrobial Susceptibility Tests for Bacteria That Grow Aerobically*; 12th ed.; CLSI: Wayne, PA, 2024.

[48] Vaux, D. J. *Method and Apparatus for Measuring Surface Configuration*. WO 2007/039729 A1, 2007.

[49] Walter, V.; Syldatk, C.; Hausmann, R. Screening Concepts for the Isolation of Biosurfactant-Producing Microorganisms. *Adv. Exp. Med. Biol.* **2010**, *672*, 1–13.

[50] Dose, B.; Ross, C.; Niehs, S. P.; Scherlach, K.; Bauer, J. P.; Hertweck, C. Food-Poisoning Bacteria Employ a Citrate Synthase and a Type II NRPS To Synthesize Bolaamphiphilic Lipopeptide Antibiotics. *Angew. Chem. Int. Ed.* **2020**, *59* (48), 21535–21540.

[51] Jain, D. K.; Collins-Thompson, D. L.; Lee, H.; Trevors, J. T. A Drop-Collapsing Test for Screening Surfactant-Producing Microorganisms. *J. Microbiol. Methods* **1991**, *13* (4), 271–279.

[52] Liew, C. W.; Nilsson, M.; Chen, M. W.; Sun, H.; Cornvik, T.; Liang, Z.-X.; Lescar, J. Crystal Structure of the Acyltransferase Domain of the Iterative Polyketide Synthase in Enediyne Biosynthesis. *J. Biol. Chem.* **2012**, *287* (27), 23203–23215.

[53] Haydock, S. F.; Aparicio, J. F.; Molnár, I.; Schwecke, T.; Khaw, L. E.; König, A.; Marsden, A. F.; Galloway, I. S.; Staunton, J.; Leadlay, P. F. Divergent Sequence Motifs Correlated with the Substrate Specificity of (Methyl)malonyl-CoA:ACP Transacylase Domains in Modular Polyketide Synthases. *FEBS Lett.* **1995**, *374* (2), 246–248.

[54] Minowa, Y.; Araki, M.; Kanehisa, M. Comprehensive Analysis of Distinctive Polyketide and Nonribosomal Peptide Structural Motifs Encoded in Microbial Genomes. *J. Mol. Biol.* **2007**, *368* (5), 1500–1517.

[55] Yadav, G.; Gokhale, R. S.; Mohanty, D. Computational Approach for Prediction of Domain Organization and Substrate Specificity of Modular Polyketide Synthases. *J. Mol. Biol.* **2003**, *328* (2), 335–363.

[56] Caffrey, P. Conserved Amino Acid Residues Correlating with Ketoreductase Stereospecificity in Modular Polyketide Synthases. *ChemBioChem* **2003**, *4* (7), 654–657.

[57] Keatinge-Clay, A. T. A Tylosin Ketoreductase Reveals How Chirality Is Determined in Polyketides. *Chem. Biol.* **2007**, *14* (8), 898–908.

[58] Reid, R.; Piagentini, M.; Rodriguez, E.; Ashley, G.; Viswanathan, N.; Carney, J.; Santi, D. V.; Hutchinson, C. R.; McDaniel, R. A Model of Structure and Catalysis for Ketoreductase Domains in Modular Polyketide Synthases. *Biochemistry* **2003**, *42* (1), 72–79.

[59] Keatinge-Clay, A. Crystal Structure of the Erythromycin Polyketide Synthase Dehydratase. *J. Mol. Biol.* **2008**, *384* (4), 941–953.

[60] Robbins, T.; Kapilivsky, J.; Cane, D. E.; Khosla, C. Roles of Conserved Active Site Residues in the Ketosynthase Domain of an Assembly-Line Polyketide Synthase. *Biochemistry* **2016**, *55* (32), 4476–4484.

[61] Weissman, K. J.; Hong, H.; Popovic, B.; Meersman, F. Evidence for a Protein–Protein Interaction Motif on an Acyl Carrier Protein Domain from a Modular Polyketide Synthase. *Chem. Biol.* **2006**, *13* (6), 625–636.

[62] Stachelhaus, T.; Mootz, H. D.; Bergendahl, V.; Marahiel, M. A. Peptide Bond Formation in Nonribosomal Peptide Biosynthesis: Catalytic Role of the Condensation Domain. *J. Biol. Chem.* **1998**, *273* (35), 22773–22781.

[63] Masschelein, J.; Clauwers, C.; Awodi, U. R.; Stalmans, K.; Vermaelen, W.; Lescrinier, E.; Aertsen, A.; Michiels, C.; Challis, G. L.; Lavigne, R. A Combination of Polyunsaturated Fatty Acid, Nonribosomal Peptide, and Polyketide Biosynthetic Machinery Is Used To Assemble the Zeamine Antibiotics. *Chem. Sci.* **2015**, *6* (2), 923–929.

[64] Wenski, S. L.; Cimen, H.; Berghaus, N.; Fuchs, S. W.; Hazir, S.; Bode, H. B. Fabclavine Diversity in *Xenorhabdus* Bacteria. *Beilstein J. Org. Chem.* **2020**, *16*, 956–965.
